# Origin and dispersal of early domestic pigs in northern China

**DOI:** 10.1038/s41598-017-06056-8

**Published:** 2017-08-10

**Authors:** Hai Xiang, Jianqiang Gao, Dawei Cai, Yunbing Luo, Baoquan Yu, Langqing Liu, Ranran Liu, Hui Zhou, Xiaoyong Chen, Weitao Dun, Xi Wang, Michael Hofreiter, Xingbo Zhao

**Affiliations:** 1 National Engineering Laboratory for Animal Breeding; Ministry of Agricultural Key Laboratory of Animal Genetics, Breeding and Reproduction; and College of Animal Science and Technology, China Agricultural University, Beijing, 100193 China; 2 Institute of Genetics and Developmental Biology, Chinese Academy of Sciences, Beijing, 100101 China; 3 Hebei Provincial Institute of Cultural Relic, Shijiazhuang, 050031 China; 4 Ancient DNA Laboratory, Research Center for Chinese Frontier Archaeology, Jilin University, Changchun, 130023 China; 5 Hubei Provincial Institute of Cultural Relics and Archaeology, Wuhan, 430077 China; 6 Xushui County Office for Preservation of Ancient Monuments, Xushui, 072550 China; 7 Institute of Animal Sciences, Chinese Academy of Agricultural Sciences, Beijing, 100193 China; 8 Institute of Animal Science and Veterinary of Hebei Province, Baoding, 071000 China; 9 Institute of Animal Science and Veterinary Medicine, Shanxi Academy of Agricultural Science, Taiyuan, 030032 China; 10 Faculty of Mathematics and Natural Sciences, Institute for Biochemistry and Biology, University of Potsdam, Karl-Liebknecht-Str. 24-25, Potsdam, 14476 Germany

## Abstract

It is widely accepted that modern pigs were domesticated independently at least twice, and Chinese native pigs are deemed as direct descendants of the first domesticated pigs in the corresponding domestication centers. By analyzing mitochondrial DNA sequences of an extensive sample set spanning 10,000 years, we find that the earliest pigs from the middle Yellow River region already carried the maternal lineages that are dominant in both younger archaeological populations and modern Chinese pigs. Our data set also supports early Neolithic pig utilization and a long-term *in situ* origin for northeastern Chinese pigs during 8,000–3,500 BP, suggesting a possibly independent domestication in northeast China. Additionally, we observe a genetic replacement in ancient northeast Chinese pigs since 3,500 BP. The results not only provide increasing evidence for pig origin in the middle Yellow River region but also depict an outline for the process of early pig domestication in northeast China.

## Introduction

It is widely accepted that pigs were domesticated independently in Near East and East Asia beginning ~10,000 years ago after *Sus sp*. emerged in Southeast Asia during the climatic fluctuations of the early Pliocene 5.3–3.5 My ago^1–3^. So far, at least six phylogeographically distinct wild boar lineages have been found to have contributed to the present domestic pig populations^4^. Over the past decade, regions in China, including the Mekong River basin^5^, the downstream region of the Yangtze River^5^, the upper stream region of the Yangtze River^6^, the Tibetan highlands^7^ and the lower region of the Yellow River^8, 9^ have been suggested as regions from which wild boar have contributed to the domestic pig gene pool and which may have represented independent centers for pig domestication.

Zoo-archaeological studies on the pig remains from the Early Neolithic assemblages in China back this idea of multiregional pig domestication in East Asia. The faunal assemblages of the ~9,000-y-old Zhenpiyan cave site^10^ in Guangxi province and the ~8,000-y-old Kuahuqiao site^11^ in Zhejiang Province both provide evidence for a commensal domestication phase^12^ in the Mekong River basin and the downstream region of Yangtze River. Furthermore, numerous pig remains excavated in the ~10,500-y-old Nanzhuangtou site^13^, the ~9,000-y-old Jiahu site^14^, and the ~7,500-y-old Cishan site^15^ as well as palaeogenetic studies^8^ presented the middle and lower branches of the Yellow River as one of the earliest regions for pig domestication. Extensive archaeological findings in these regions, such as broomcorn millet, foxtail millet, dog and chicken, are evidence for early origins of mixed agriculture^16–18^. Besides the archaeological findings, pigs seem to have had cultural significance for the communities in this region because two pig skeletons were buried with humans at Xinglongwa Site (8,200–7,400 BP), implying either that pigs may already have been domesticated in Northeast China around 8,000 years ago or that wild boar had cultural significance^19, 20^.

It has previously been shown that, at least with regard to maternal lineages, Chinese native pig breeds were direct descendants of the first domesticated pigs in the corresponding geographical regions^8, 9^. However, it is likely that roaming in a semi-managed status during the beginning of domestication allowed recurrent genetic exchange between domesticated pigs and indigenous wild boars. Thus, even during the beginning of domestication, genetic similarity between domestic pigs and wild boar from the same region may indicate introgression of wild boar haplotypes into the domestic gene pool rather than independent domestication events. Moreover, human migrations and cultural expansions repeatedly took place in East Asia during the past 10,000 years. Thus, human-mediated dispersal of and gene flow among domestic pig populations would not have been uncommon. Both for European and Near Eastern pigs, ancient DNA and dental geometric morphometric analyses have revealed nonlinear patterns of early domestication, dispersal of animals, genetic turnover and interaction among various geographic regions^21–23^.

**Figure 1:**
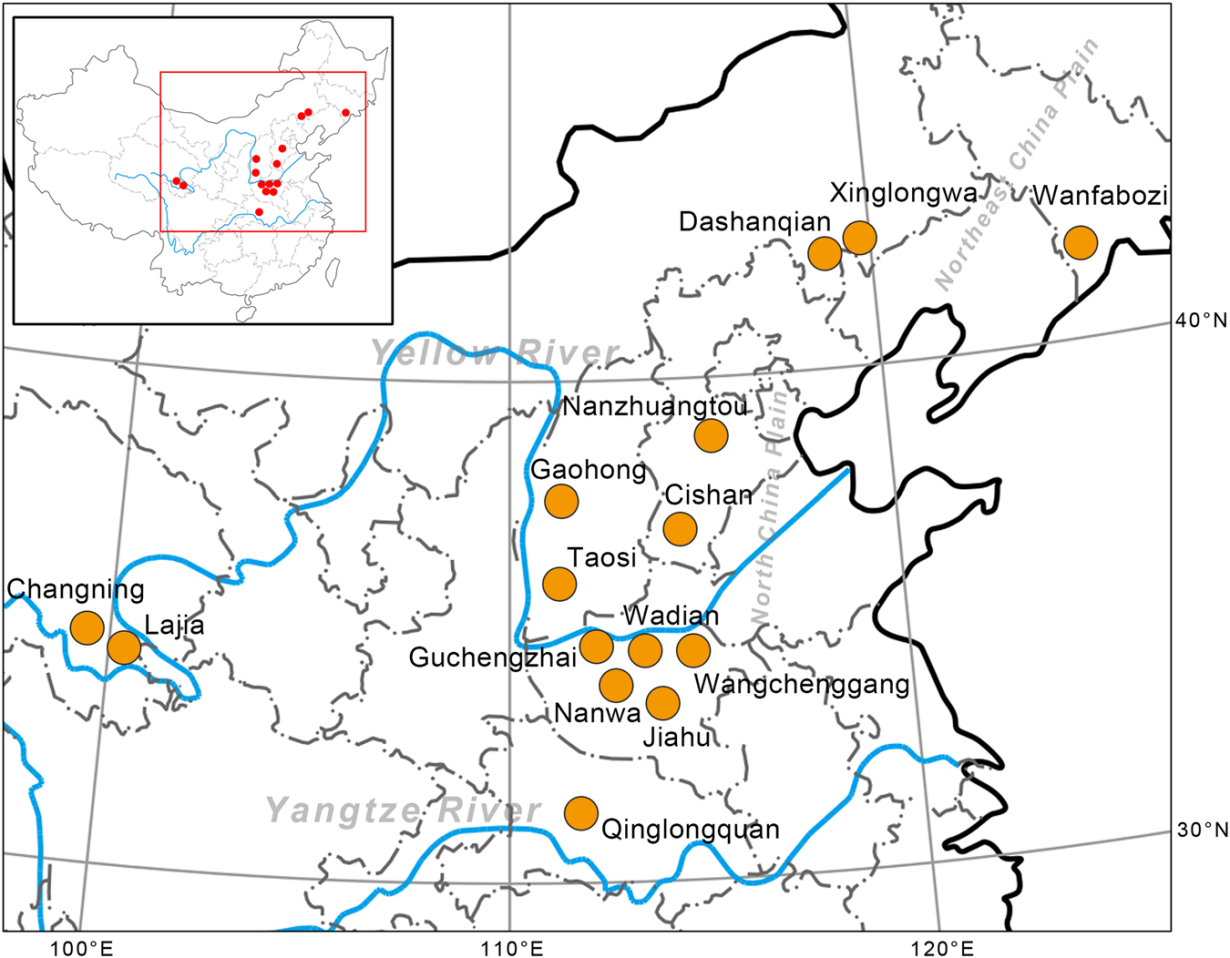
Locations of samples. The orange dots depict all sites from which we retrieved ancient pig sequences. The inset shows the location of the region within China. The maps are modified from free map materials deposited in the public database of National Administration of Surveying, Mapping and Geoinformation (http://219.238.166.215/mcp/index.asp).

We have collected pig remains from 15 archaeological sites in China, including Jiahu and another two of the oldest archaeological sites in the middle Yellow River basin (Nanzhuangtou and Cishan), three sites in Northeast China (Xinglongwa, Wanfabozi and Dashanqian), and one site (Changning) in the upper region of the Yellow River. We investigated partial mitochondrial DNA control region (CR) sequences and a segment of the cytochrome b gene (*Cytb*) from both ancient and modern pigs, aiming to uncover the pattern of early maternal domestication and lineage dispersal in China. Locations and details for all samples can be found in Fig. 1 and Table S1.

## Results

Using loop-mediated PCR (L-PCR) followed by a specific singleplex PCR (for primers used see Table S2), we retrieved 49 *Cytb* fragments (Table S3) and 15 CR fragments (Table S4) of ancient pig samples from 15 archaeological sites. By combining our new results with previously reported sequences, we obtained a data set of 38 ancient pig CR sequences from China (Table S4) and combined *Cytb*/CR sequences from 37 samples, ranging geographically from northeast to northwest China and temporally from 10,000 BP to 2,500 BP (Table S1). We confirmed the Neolithic context of some of the early samples by determining the ages of the archaeological pig bones by direct radiocarbon dating of two bones from Cishan site, one bone from Nanzhuangtou site and one bone from Xinglongwa site. These samples yielded calibrated dates of ∼7,600, ∼7,800, ∼10,500 and ∼7,500 years, respectively (Tables S1 and S5), confirming the archaeologically determined ages.

**Figure 2:**
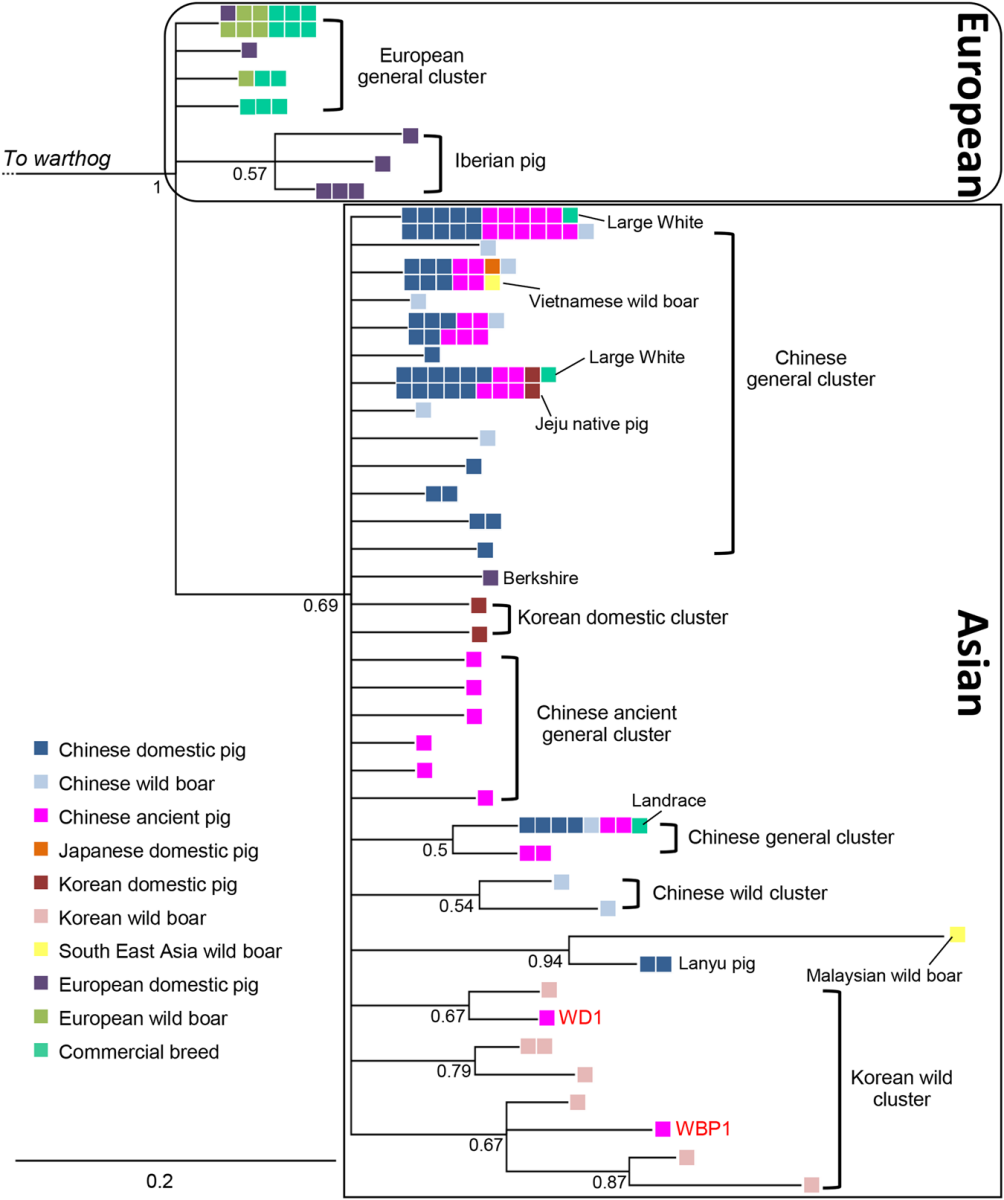
Bayesian consensus phylogenetic tree of the 37 combined (*Cytb* and control region) ancient pig sequences and 98 extant published homologous sequences. Ages (modern vs. ancient), status (wild vs. domestic) and geographical origin are depicted in different colours (see bottom left of the figure). The European and Asian sequence clusters are framed.

We aligned the 37 combined sequences with 97 published homologous sequences of extant wild boar, native pig breeds and commercial pig breeds from all over the world (Table S6). Phylogenetic analyses resulted in two apparent clusters, which represent Asian and European origins, respectively (Fig. 2). All 10,000 BP to 2,500 BP Chinese pigs were found in the Asian clade (Fig. 2).

**Figure 3:**
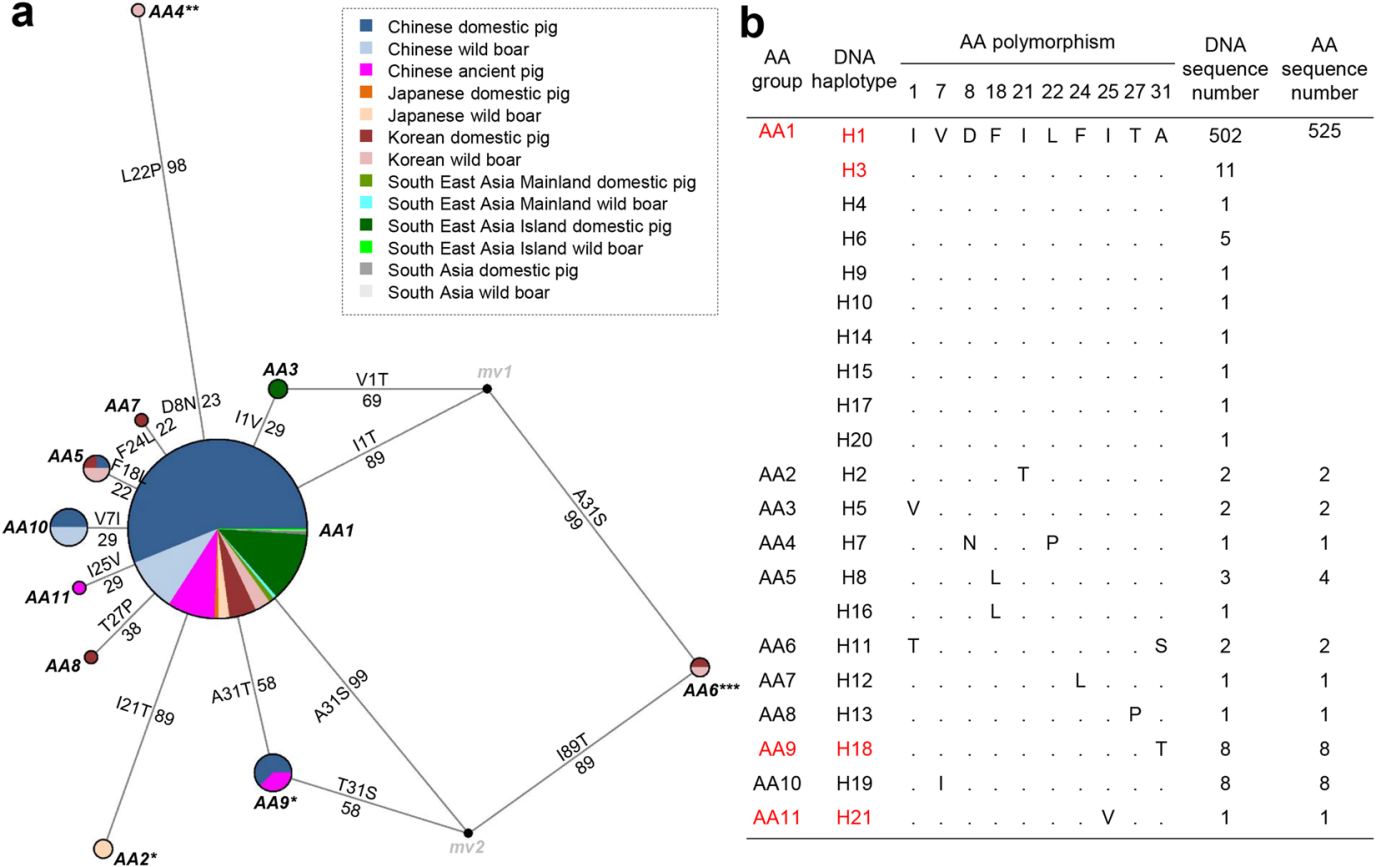
Ancient *Cytb* gene analyses. Relationship of pig *Cytb* amino acid sequences. (**a**) Each haplogroup is represented by a circle, with the area of the circle proportional to the haplogroup’s frequency. Different colors indicate samples originating from different regions. The numbers alongside AA changes are the Grantham scores which help classify the conservation levels of AA changes. The number of asterisks highlights the level: non - considered conservative; one - moderately conservative; two - moderately radical and three - radical, respectively. (**b**) Alignment of AA changes among the 11 AA haplogroups and 21 DNA haplotypes. The AA haplogroups and DNA haplotypes containing ancient pigs are in red font.

To investigate the source of the genetic diversity in Asian pigs in more detail, we compared ancient and modern Asian pigs based on the mitochondrial *Cytb* sequences alone. We aligned our 49 ancient sequences (Table S3) with 506 extant homologous sequences, which represent modern Asian pigs deposited in GenBank (Table S7). We found 20 SNPs among all Asian pig sequences, which constituted 21 haplotypes (Table S7). The 20 SNPs included 10 nonsynonymous substitutions, by which we classified Asian pigs into 11 groups defined by different *Cytb* amino acid sequences. The group AA1 contained 10 haplotypes representing 525 sequences (almost 95% of the sequences) and all geographically defined pig types from Asia (Fig. 3a and b), making this haplogroup the predominant haplogroup among Asian pigs. The groups AA2-AA11 contained the remaining 30 sequences, representing pigs from China, Japan, Korea and South East Asia Island, with one to maximally two AA substitutions to AA1 in this region of *Cytb* (Fig. 3a and b, Table S7) with various Grantham conservative scores^24, 25^.

The 38 ancient CR sequences obtained (Table S4) were aligned with 1,716 published homologous sequences from China, including 1,495 modern native domestic pigs and 221 wild boar (Table S8), representing 73 haplotypes (Table S8). Using the warthog (*P. africanus*) as outgroup, the consensus Bayesian tree resulted in three clades and a large polytomy representing the majority of the sequences (Fig. S1). The largest one (in red) of the three clades consisted of 8 haplotypes representing 12 Tibet Plateau pig samples and 1 haplotype representing 1 wild boar and 1 domestic pig each from southwest China. The other two clades (in green) were formed only by wild boars, in which one contained 2 haplotypes (representing 9 sequences) and the other one contained 3 haplotypes (25 sequences). The polytomy consisted of numerous branches, where three haplotypes were found only in ancient samples, 21 haplotypes were found only in wild boar, and 20 haplotypes only in modern domestic pigs, 6 of which were found only in Plateau pigs, while the remaining nine haplotypes were found in wild boars, ancient samples and modern domestic pigs (Table S8).

The 38 CR sequences obtained from ancient samples constitute 11 haplotypes (Table 1 and Fig. S2). Haplotypes H2 and H3 first appeared in Nanzhuangtou samples from the middle Yellow River valley in northern China at least 10,500 BP (~10,000 yrs for H2 from the indirectly dated dNZT1-3 sample and, ~10,500 yrs for H3 from the directly dated sample dNZT4). We also found haplotype H3 in sample dJH1 from Jiahu dated to ~9,000 BP. Haplotype H4 was first found in sample dJH2 also from Jiahu dating to at least ~8,500 BP. We also found it in sample dCS2 from Cishan (directly dated to ~7,800 yrs), and at Xinglongwa in Northeast China dating to 7,400–8,000 BP (sample dXLW1). The ancient sample dXLW2 from Xinglongwa, directly dated to ~7,500 BP, yielded haplotype H18, which also appeared in sample dWD2 from Wadian in the middle Yellow River area dating to ~4,000 BP. The ancient sample dCS1 from Cishan, directly dated to ~7,600 yrs, yielded haplotype H19 which also emerged in sample dWD3 from Wadian (~4,000 BP). We also found five rare haplotypes amongst our ancient samples. Two younger samples from Northeast China were identified as the rare haplotypes H31 (sample dWBP1 from Wanfabozi, 5,000–4,000 BP) and H70 (sample dDSQ2 from Dashanqian, 4,000–3,500 BP), while three ~4,000-y-old samples from the middle Yellow River region, dWD1, dGCJ1 and dGCJ3, yielded the rare haplotypes H71, H72 and H73, respectively. Except for those samples carrying rare haplotypes, ancient samples younger than 4,500 yrs carried haplotype H10 or haplotypes H2, H3 and H4 that were already found in the oldest samples (Table 1 and Fig. S2).

**Table 1:** Haplotype composition of ancient samples for control region sequences.

Region	Site	Age (BP)	H2	H3	H4	H10	H18	H19	H31	H70	H71	H72	H73	Total
Middle Yellow River Region	Nanzhuangtou	10,500–9,700	3	1										4
	Jiahu	9,000–8,200		1	1									2
	Cishan	7,785–7,575			1			1						2
	Wadian	4,150–3,950					1	1		1				9
	Guchengzhai	4,150–3,950										1	1	
	Taosi	4,350–3,850	1			3								
	Wangchenggang	3,550–3,100	1											7
	Gaohong	3,500–3,200		2		4								
Upper Yellow River Region	Lajia	~4,000				3								4
	Changning	4,200–3,700			1									
Middle Yangtze River Region	Qinglongquan	4,400–4,200	2	1	2									5
Northeast China	Xinglongwa	8,200–7,400			1		1							2
	Wanfabozi	5,000–4,000							1					1
	Dashanqian	4,000–3,500				1					1			2
Ancient total			7	5	6	11	2	2	1	1	1	1	1	38
Modern			416	281	223	427	41	39	2	1	0	0	0	1430

**Figure 4:**
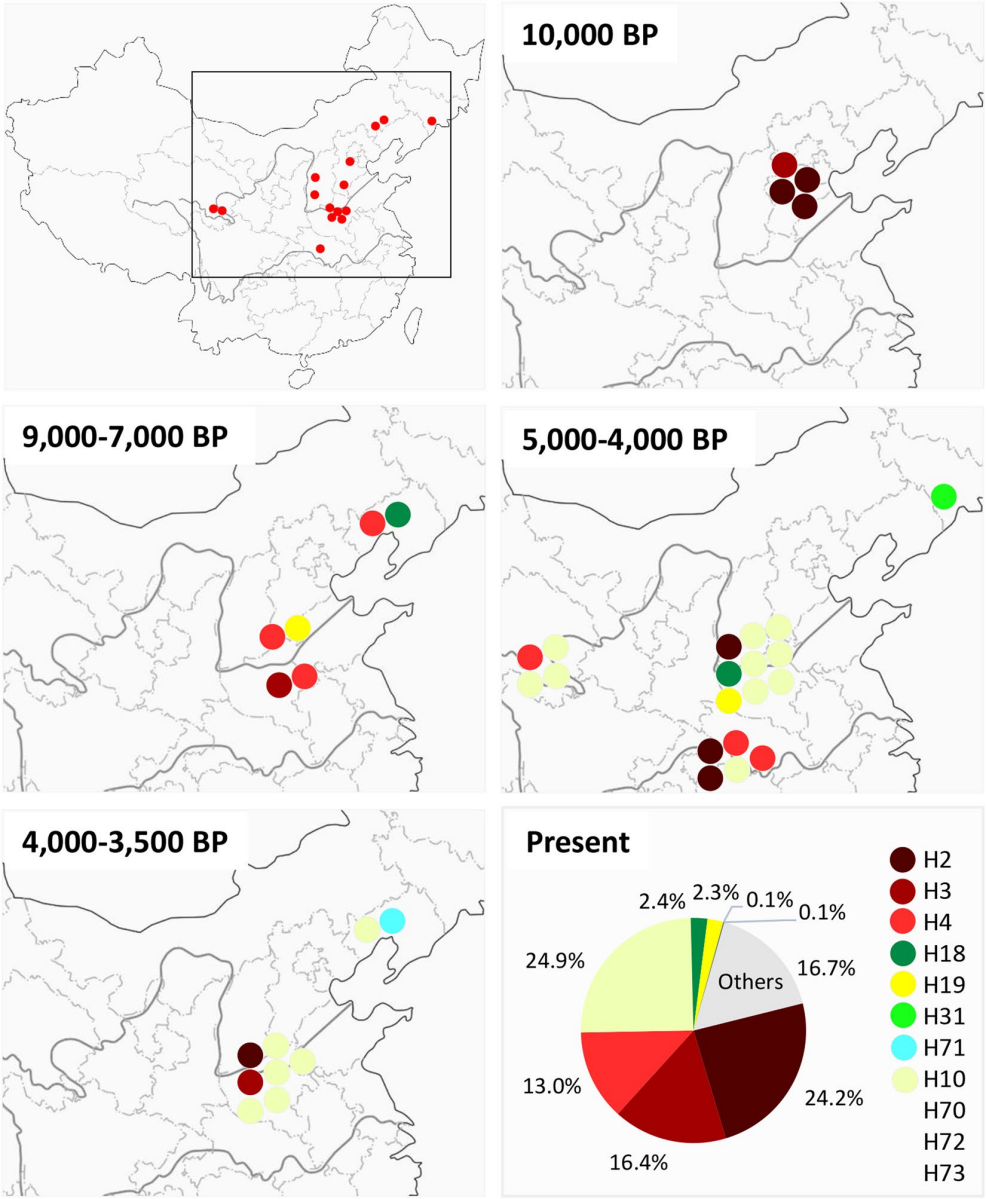
Temporal transition of mtDNA control region haplotypes of ancient Chinese pigs in different regions. The upper left map shows the location of the investigated region within China. From left to right and top to bottom: Time series of maps identifying the locations and haplotypes of ancient pig samples from which DNA sequences were obtained. Each symbol corresponds to a single sample. Different colours indicate different haplotypes (see lower right panel of the figure). The pie chart at the lower right shows the proportions of the different haplotypes in present day Chinese pigs. Maps are modified from free map materials deposited in the public database of National Administration of Surveying, Mapping and Geoinformation (http://219.238.166.215/mcp/index.asp).

Together, the four haplotypes H2, H3, H4 and H10 accounted for 1,376 of the 1,754 investigated sequences (almost 80%), clearly representing the dominant haplotypes found in Chinese pigs (Table 1 and Fig. S3). Moreover, haplotypes that appeared before 7,000 BP in the middle Yellow River valley, including the dominant haplotypes H2, H3 and H4 as well as the less dominant haplotype H19, were repeatedly found in different historical sites, and represent a large part of the haplotypic diversity found in modern Chinese pigs (~55% of modern pigs carry these haplotypes; Fig. 4 and Table 1). In contrast, the fourth dominant haplotype, H10, which is found in ~25% of modern Chinese pigs, was detected only around 4,500 years ago in several sites across China within our data set (Fig. 4 and Table 1). Other contemporaneous haplotypes H70, H72 and H73 were generally detected almost exclusively in ancient pigs, except H70, which we found in one modern pig (Table 1). These three rare haplotypes are genetically close to the dominant haplotypes H2 or H10, separated by only one or two SNPs. A similar picture arises in Northeast China, where the ~4,000 y old haplotypes H31 and H71 were found, which are close to H2 and H10, respectively. While we did not find H71 in modern pigs, H31 interestingly appeared in two modern wild populations (Table S8). Moreover, the 8,000–7,400 years old remains in Northeast China carried not only the dominant haplotype H4 but also the less dominant haplotype H18. Even though H18 was never found in modern samples from Northeast China, it was detected in a younger sample (4,000 BP) from Wadian from the middle Yellow River region and at ~2.4% in modern pig populations in southern China (Fig. 4 and Table 1). Finally, for the ~4,000 year old samples from the upper region of the Yellow River and the middle region of the Yangtze River, we found that they shared similar haplotype compositions with ancient pigs in the middle Yellow River basin (Fig. 4). Overall, after their first appearance in the middle region of the Yellow River and Northeast China, most of the oldest haplotypes were inherited into younger times up to modern Chinese pigs (Fig. 4 and Table 1).

## Discussion

It is generally accepted that pigs were domesticated in at least two separate domestication centers, Europe and Asia^26–28^. In our data set of mitochondrial DNA sequences from ancient Chinese pigs, we found both considerable differences in haplotype composition over time and long-term continuity of some haplotypes.

The middle Yellow River basin has long been discussed as one center for early Chinese pig domestication based on both ancient DNA and morphometric evidence^8, 14, 29^. Already the earliest pig remains from this region revealed the haplotypes that represent the dominant maternal lineages in modern Chinese pigs (Table 1), supporting the hypothesis that the middle Yellow River basin indeed was one of the earliest centers for pig domestication from where domestic pigs spread to other regions in China. In a previous study, we demonstrated maternal lineage continuity in pigs from between ~9,000-y-old remains in the middle and ~4,000-y-old ones in the upper region of the Yellow River^9^. The present results from two samples from Changning in the upper region of the Yellow River further support this interpretation. Moreover, isotope data from Dadiwan in the upper Yellow River region provided evidence for a cultural replacement by central Chinese Neolithic cultures before 4,000 BP^30^, suggesting a possible reason why the ~4,000 BP pigs in the upper Yellow River region could have originated from progenitors from the middle Yellow River region. Similar to our previous study^9^, additional samples from Qinglongquan in the middle Yangtze River region also carry the dominant haplotypes that were already found in ~10,000 BP samples from the middle Yellow River drainage, suggesting a possible southward migration before (or around) ~4,000 year ago.

Due to close relationships between humans and pigs and evidence of early millet farming at Xinglongwa, Northeast China has been proposed as another region of early pig domestication^20^. However, only pig remains younger than 8,000 BP are assumed to represent domesticated ones^19^. In the present study, Xinglongwa specimens carry the dominant modern haplotype H4 and the less common haplotype H18, respectively, demonstrating maternal continuity between the earliest archaeological pig remains in this region and modern Chinese domestic pigs (Fig. 4 and Table 1). The haplotype H4 also appears in Jiahu and Cishan around the same time (Fig. 4), suggesting either early population migrations, trade of early domestic pigs or parallel domestication across the North China Plain and Northeast China. *Suidae* remains in Xinglongwa were found in all three cultural phases (8,200–8,000 BP, 8,000–7,400 BP and 7,400–7,000 BP)^19^, suggesting continuous utilization of pigs during the early Neolithic in this region. The younger haplotypes H31 and H71 discovered in Northeast China (Wanfabozi and Dashanqian) have so far not been found in the middle Yellow River region (Fig. 4 and Table 1), implying also long-term *in situ* continuity after initial pig domestication, or possibly, introgression of wild boar haplotypes into imported, early domestic pig herds, in Northeast China.

In addition to long-term continuity, we also observed genetic turnovers in ancient Northeast Chinese pigs. One example is haplotype H18, which we found in a ~7,500 year old sample from Xinglongwa, but which seems to have disappeared afterwards in Northeast China. However, we found it in younger samples from the middle Yellow River region ~4,000 BP (Fig. 4 and Table 1) and also in modern pigs from southern China. In contrast, the rare haplotype H71, which we found in a single ~3,500 year old sample from Northeast China has not yet been found in modern pigs (Table 1), indicating that genetic drift also played a role in the history of domestic pigs during the last 3,500 BP. This view is further supported by haplotypes H72 and H73, which are both from the ~4,000 BP Guchengzhai site in the middle Yellow River Region and have not been found in any younger sites or modern pigs so far.

The last pattern was found for haplotype H31 (Table S7), which we discovered only in one 4,000–5,000 year old sample from Northeast China and in two wild boar from southern China. In contrast to past pig populations, modern pigs in Northeast China show a similar mitochondrial haplotype composition as other regions (Fig. S3 and Table S7), suggesting extensive homogenization of pig populations over time, accompanied by a loss of at least some of the original, regional maternal lineages.

## Conclusion

Our results support early independent domestication and long-term genetic continuity of pigs both in Europe and East Asia. The earliest pigs from the middle Yellow River region already carried the maternal lineages that are dominant in both younger archaeological populations and modern Chinese pigs. Moreover, there is strong evidence for early Neolithic pig utilization and possibly independent domestication, or alternatively, incorporation of local wild boar into introduced domestic pig populations in northeast China. However, this *in situ* origin for northeastern Chinese pigs ended around 3,500 BP due to genetic replacement, likely by pigs originating from the middle Yellow River region.

## Materials and Methods

### Sample Information

We used ninety-three ancient pig specimens (bones or teeth) for DNA analyses, which originate from 15 archaeological sites in northern and central China (Fig. 1), including 65 specimens from nine sites in the lower and middle area of the Yellow River drainage basin (Nanzhuangtou and Cishan sites in Hebei province; Jiahu, Nanwa, Guchengzhai, Wadian and Wangchenggang sites in Henan province; Taosi and Gaohong sites in Shanxi province), 6 specimens from two sites located in the upper region of the Yellow River (Lajia and Changning sites in Qinghai province), 12 specimens from one site (Qinglongquan site in Hubei province) located in the middle region of the Yangtze River, and 10 specimens from three sites in Northeast China (Xinglongwa and Dashanqian sites in eastern Inner Mongolia and Wangfabozi site in Jilin province). Dates of these archaeological sites ranged from 10,600 to 2,500 years before present. Details regarding the archaeological sites and the contexts from which the specimens were recovered can be found in Table S1.

### Sample Preparation and Ancient DNA Extraction

All pre-PCR work was conducted in the Ancient DNA Laboratory at China Agricultural University. Samples were prepared by cautiously cleaning the adhering soils and other external contaminations using abrasive paper, and then washing them with 5% (vol/vol) sodium hypochlorite solution followed by double-distilled water and drying under UV-irradiation. After that, bones and teeth were ground to fine-grained powder.

DNA was extracted by using the QIAamp DNA Investigator kit (Qiagen) and Amicon Ultra-4 filters (Millipore). DNA extraction followed the QIAamp DNA Investigator handbook for purification of total DNA from bones or teeth. Amicon Ultra-4 (Millipore, 10 K) filters were used to concentrate ancient DNA to a final volume of ∼50 μL. Several mock extractions were carried out alongside the samples in the same manner to monitor for contamination.

### Amplification and Sequencing of Ancient DNA

We used loop-mediated PCR (L-PCR) followed by a specific singleplex PCR amplification and Sanger sequencing to obtain the targeted ancient pig DNA sequences. As previously described^18^, L-PCR is designed to efficiently enrich the target copy number using loop-mediated isothermal amplification primer sets; subsequent singleplex PCR then allows generating a specific amplicon that can then be sequenced.

Primers were designed according to the published mitochondrial sequence of *S. scrofa* (NC_000845). All L-PCR primers were designed by online loop-mediated isothermal amplification primer designing software (primerexplorer.jp/e/). The primer set of the mitochondrial *Cytb* gene was newly designed for this study. The L-PCR primer pair of the mitochondrial control region was designed based on the control region PCR primer pair described by Larson *et al*.^8^. Amplicons of the *Cytb* gene and the control region were 101 bp and 138 bp in size, respectively, and contained polymorphic sites which were used to define haplogroups or haplotypes. Detailed primer sequences are available in Table S2.

L-PCR and the specific singleplex PCR amplification were setup as previously described^18^. Several blank controls were performed in all PCR assays. L-PCR used the following cycling conditions: 37 °C for 10 min, 94 °C for 5 min, followed by 35 cycles of 94 °C for 30 s, 65 °C for 40 s, 72 °C for 30 s, and a final extension of 10 min at 72 °C. Secondary PCR used the following cycling conditions: 37 °C for 10 min, 94 °C for 5 min, followed by 35 cycles of 94 °C for 30 s, 61 °C for 30 s, 72 °C for 30 s, and a final extension of 10 min at 72 °C. Amplifications of the extraction blank controls and PCR blank controls were performed in all experiments to monitor contaminations. Amplification success was controlled by electrophoresis on a 2% agarose gel. Subsequently, PCR products were purified using the QIAquick PCR purification kit (Qiagen). Sequencing was carried out on an ABI 3730XL automated DNA sequencer (Applied Biosystems) using the ABI Prism Big Dye Terminator v3.1 Cycle Sequencing kit.

### Independent Replication Experiments

For all ancient DNA samples, we performed several replication experiments in the Ancient DNA Laboratory at China Agricultural University. Moreover, some of the positive samples were sent to the Ancient DNA Laboratory at the Research Center for Chinese Frontier Archeology at Jilin University for independent replication experiments (Table S1). DNA extraction, amplification and sequencing were carried out using the same methods and conditions in both laboratories.

### Radiocarbon Dating Analysis

All samples used in the present study were from well-defined archaeological contexts. Nevertheless, four ancient bones from the three key archaeological sites of Nanzhuangtou (1 bone), Cishan (2 bones) and Xinglongwa sites (1 bone), which yielded DNA sequences were sent for radiocarbon dating using direct accelerator mass spectrometry at Beta Analytic Inc. (USA) to provide further support of their ages (Table S5).

### Data Analyses

The reconstructed ancient DNA sequences were aligned with extant published homologous sequences from wild boars, local domestic pigs and commercial breeds from all over the world (Table S6). The ancient *Cytb* gene sequences (Table S3) and homologous sequences of Asian *S. scrofa* were aligned and investigated considering both DNA and amino acid (AA) phylogenetic relationship (Table S7). Particularly, to determine the origin and dispersal pattern of the dominant haplotypes in Chinese pig breeds, the ancient control region sequences (Table S4) were utilized in comparison with published sequences of ancient and modern Chinese domestic pigs and wild boars (Table S8). Sequences were aligned using MUSCLE^31^ or the online tool MAFFT^32^, and edited using MEGA 6^33^; then the online tool FaBox (users-birc.au.dk/biopv/php/fabox/) was used to classify haplotypes^34^. MrBayes 3.2.3^35^ was used for phylogenetic analysis with model parameters identified by jModelTest 2.1.1^36^. The length of the MCMC was set to 10,000,000. Parameter estimates and consensus trees resulting from 10 MrBayes runs were recorded and compared. The best supported phylogenetic consensus tree was summarized with discarding the first 25% as burn-in. The tree was depicted using the software FigTree v1.4.2 (http://tree.bio.ed.ac.uk/software/figtree/). Median-joining networks^37^ were reconstructed using the software Network 4.6.1.0 (www.fluxus-engineering.com/index.htm). The aligned DNA sequence file format conversions were performed using BioEdit 7.0^38^ and Forcon 1.0^39^.

## Electronic supplementary material

Supplementary Information
