# Supplementary material for: Origin and dispersal of early domestic pigs in northern China
Source: Sci Rep. 2017 Aug 10;7:5602. doi: 10.1038/s41598-017-06056-8 (PMC5552793; doi:10.1038/s41598-017-06056-8)
Supplement: Supplementary file 1 — Supplementary Information [file 41598_2017_6056_MOESM1_ESM.pdf]

Origin and dispersal of early domestic pigs in northern China

Hai Xiang[1,2], Jianqiang Gao[3], Dawei Cai[4], Yunbing Luo[5], Baoquan Yu[6], Langqing Liu[1], Ranran Liu[7], Hui Zhou[4], Xiaoyong Chen[8]， Weitao Dun[8]， Xi Wang[9]， Michael Hofreiter[10*] and Xingbo Zhao[1,*]

[1]National Engineering Laboratory for Animal Breeding; Ministry of Agricultural Key Laboratory of Animal Genetics, Breeding and Reproduction; and College of Animal Science and Technology, China Agricultural University, Beijing 100193, China;

[2]Institute of Genetics and Developmental Biology, Chinese Academy of Sciences, Beijing 100101, China；

[3]Hebei Provincial Institute of Cultural Relic, Shijiazhuang 050031, China;

[4]Ancient DNA Laboratory, Research Center for Chinese Frontier Archaeology, Jilin University, Changchun 130023, China;

[5]Hubei Provincial Institute of Cultural Relics and Archaeology, Wuhan 430077, China;

[6]Xushui County Office for Preservation of Ancient Monuments, Xushui 072550, China;

[7]Institute of Animal Sciences, Chinese Academy of Agricultural Sciences, Beijing 100193, China;

[8]Institute of Animal Science and Veterinary of Hebei Province, Baoding, 071000 China;

[9]Institute of Animal Science and Veterinary Medicine, Shanxi Academy of Agricultural Science, Taiyuan 030032, China;

[10]Faculty of Mathematics and Natural Sciences, Institute for Biochemistry and Biology, University of Potsdam, Karl-Liebknecht-Str. 24-25, Potsdam 14476, Germany.

Corresponding Author

*Correspondence and requests for materials should be addressed to X.Z. (email: zhxb@cau.edu.cn) and M.H. (email: Michael.hofreiter@uni-potsdam.de).

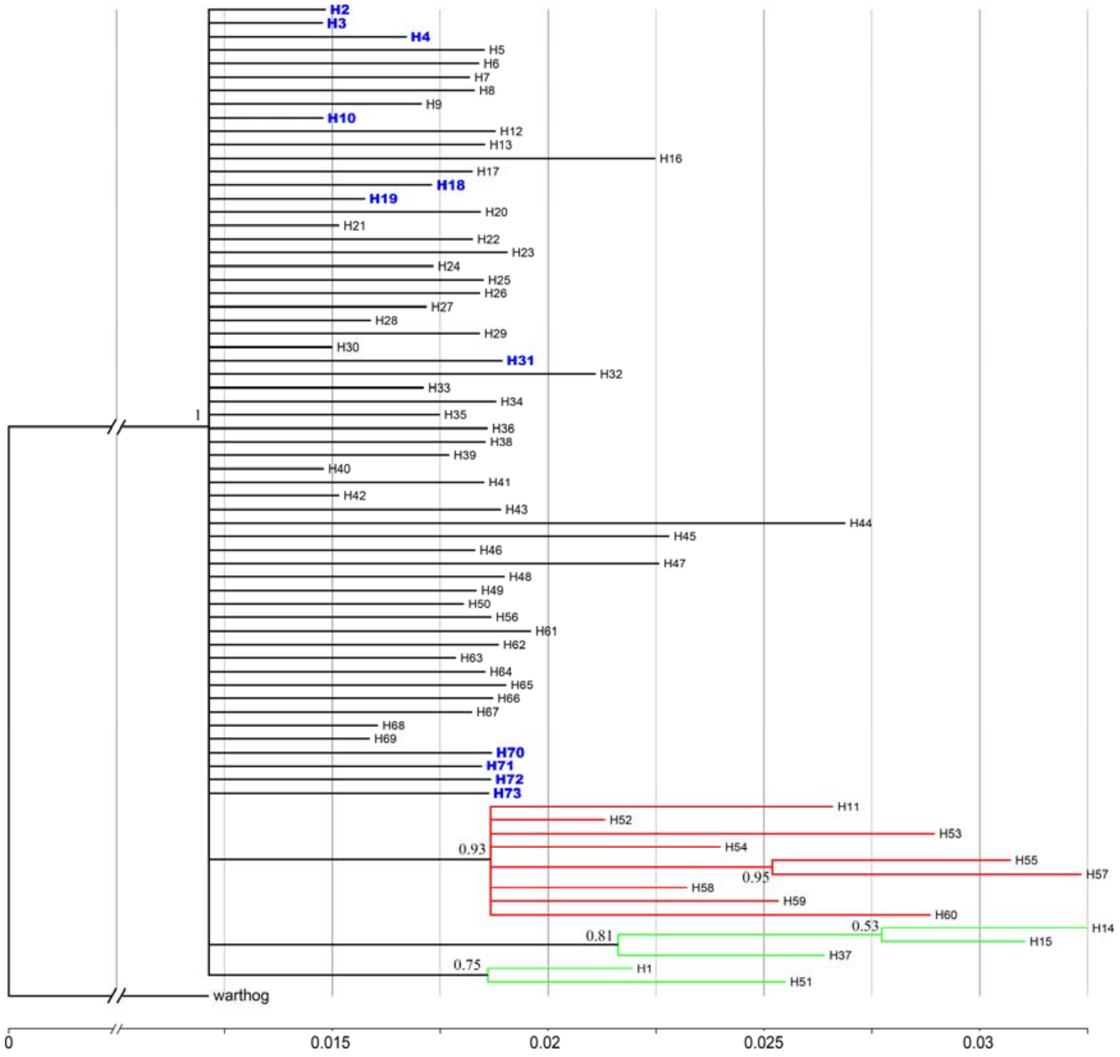

Fig. S1. Bayesian consensus phylogenetic tree of 73 control region haplotypes. Using the warthog (*P. africanus*) as outgroup, the consensus Bayesian tree shows one general clade and three compound clades. The haplotypes containing ancient samples are highlighted in blue bold.

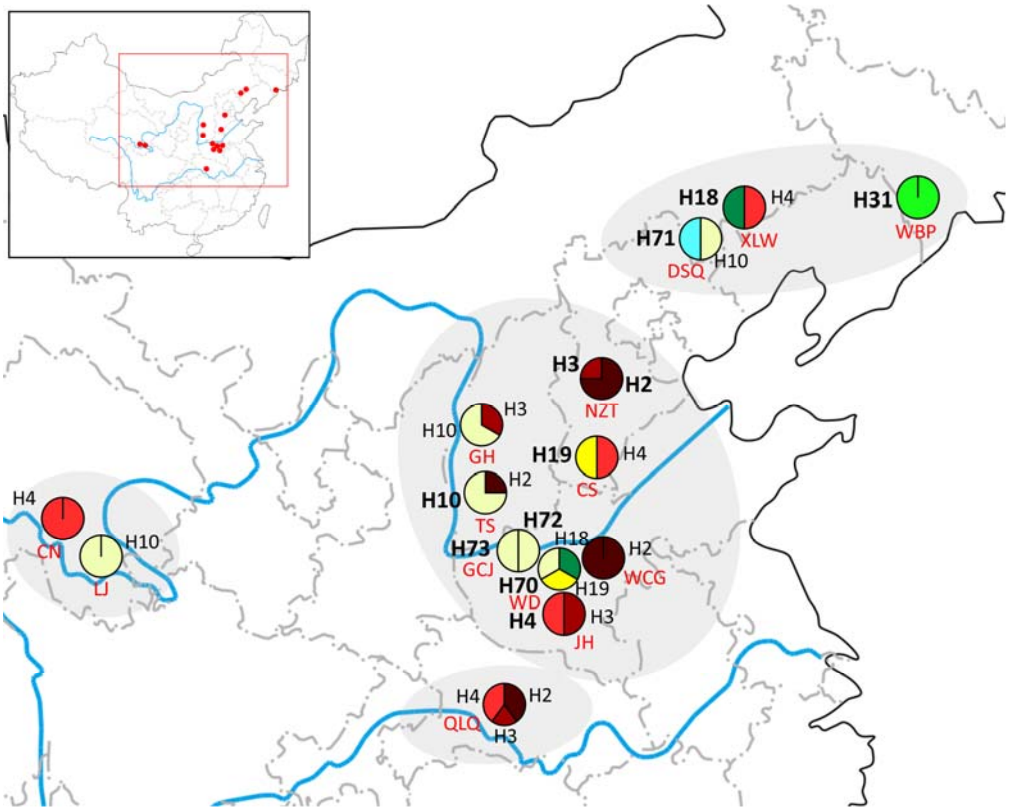

Fig. S2. Haplotype compositions of ancient samples for each archaeological site. Dot size does not indicate the number of sequences retrieved from a site. For colour codes, please refer to Fig. 4 in the main text. The maps are modified from free map materials deposited in the public database of National Administration of Surveying, Mapping and Geoinformation (http://219.238.166.215/mcp/index.asp).

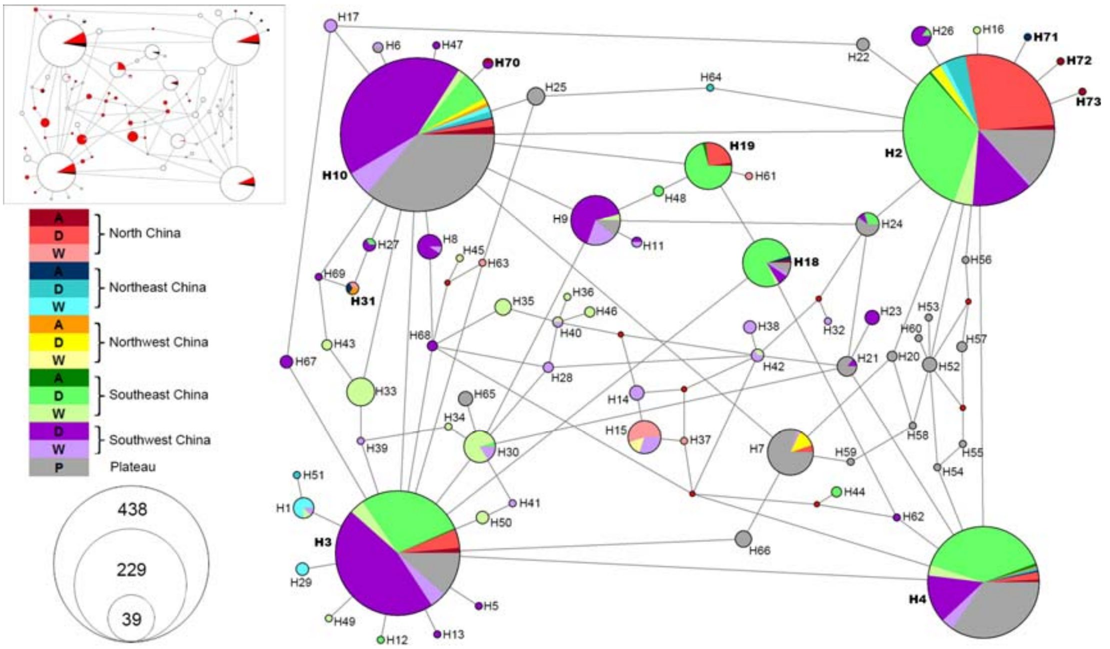

Fig. S3. Control region haplotype relationships illustrated by median-joining network analysis. Each haplotype is represented by a circle, with the area of the circle proportional to the haplotype's frequency. Different colors indicate samples originating from different regions. In the swatches panel, "A" indicates ancient samples, "D" indicates domestic pigs and "W" indicates wild boars. The haplotypes containing ancient samples are labeled in bold. Haplotypes inferred by the network analysis but not observed in the dataset-are indicated by small red dots. The insert in the upper-left corner is a skeleton diagram to show the network relationships of domestic pigs (in white), wild boars (in red) and ancient pigs (in black).

Table S1. Detailed information of ancient samples.

Region	Archaeological site	Location	Archaeological ID	Sample ID	Phase	Dates	Replication	CR+Cytb	Cytb sequence	CR sequence
	Nanzhuangtou	Xushui county, Hebei province	87xNT2⑥:064	NT1	Early Neolithic period	10,500-9,700 BP				
			86xNT1⑧:28	NT2	Early Neolithic period	10,500-9,700 BP		NZT1	cNZT1	dNZT1
			86xNT1⑧:29	NT3	Early Neolithic period	10,500-9,700 BP		NZT2	cNZT2	dNZT2
			87xNT2⑥:057	NT4	Early Neolithic period	10,500-9,700 BP				dNZT3
			87xNT2④:051	NT5	Early Neolithic period	10,500-9700 BP				
			86xNT1⑥:040	NT6	Early Neolithic period	**Cal. 10505-10270 BP**	R	NZT3	cNZT3	dNZT4
	Jiahu	Wuyang county, Henan province	T74H304	J1	Jiahu (phase Ⅰ)	9,000-8,600 BP		JH1	cJH1	dJH1
			T12H35	J2	Peiligang culture (Jiahu Ⅱ)	8,600-8,200 BP				
			T26④	J3	Peiligang culture (Jiahu Ⅱ)	8,600-8,200 BP		JH2	cJH2	dJH2
			T12H33	J4	Peiligang culture (JiahuⅢ)	8,200-7,800 BP				
			T16H75	J5	Peiligang culture (JiahuⅢ)	8,200-7,800 BP				
	Cishan	Wu'an county, Hebei province	95WCH108:6	S1	Cishan culture	7,500-7,300 BP				
			95WCH108:4	S2	Cishan culture	75,00-7,300 BP				
			95WCH108:5	S3	Cishan culture	**Cal. 7,665-7,575 BP**	R	CS1	cCS1	dCS1
			95WCH108:14	S4	Cishan culture	7,500-7,300 BP				
			95WCH108:8	S5	Cishan culture	7,500-7,300 BP				
			95WCFH110:6	S6	Cishan culture	7,500-7,300 BP			cCS2	
			95WCFH110:4	S7	Cishan culture	**Cal. 7,785-7,775 BP and Cal. 7,760-7,665 BP**	R	CS2	cCS3	dCS2
			95WCFH123:2	S8	Cishan culture	7,500-7,300 BP				
	Wadian	Yuzhou city, Henan province	T3⑤	W1	Late Longshan period	4,150-3,950 BP		WD1	cWD1	dWD1
			T3④	W2	Late Longshan period	4,150-3,950 BP				
			T4③	W3	Late Longshan period	4,150-3,950 BP				
			T3④	W4	Late Longshan period	4,150-3,950 BP		WD2	cWD2	dWD2
			T2③	W5	Late Longshan period	4,150-3,950 BP		WD3	cWD3	dWD3
			T2H61	W6	Late Longshan period	4,150-3,950 BP				
			T1H3	W7	Late Longshan period	4,150-3,950 BP				
			T3⑦	W8	Late Longshan period	4,150-3,950 BP				
			T3H61	W9	Late Longshan period	4,150-3,950 BP				
			T51(middle layer)	W10	Late Longshan period	4,150-3,950 BP				
			T20H156	GC1	Late Longshan period	4,150-3,950 BP				

Middle Yellow River Region	Guchengzhai	Xinmi city, Henan province	T72(11)	GC2	Erlitou period(phase Ⅲ)	3,560-3,500BP				
			T72(13)	GC3	Erlitou period(phase Ⅲ)	3,560-3,500BP				
			T102H70	GC4	Erlitou period(phase Ⅳ)	3,500-3,470 BP				
			T21⑦	GC5	Late Longshan period	4,150-3,950 BP		GCJ1	cGCJ1	dGCJ1
			T97H9	GC6	Late Longshan period	4,150-3,950 BP		GCJ2	cGCJ2	dGCJ2
			T102H71	GC7	Erlitou period(phase Ⅲ)	3,560-3,500BP				
			T200⑧	GC8	Late Longshan period	4,150-3,950 BP				
			T20⑥	GC9	Erligang period(phaseⅣ)	3,550-3,100 BP				
	Nanwa	Dengfeng city, Henan province	06DNIIT6206H35	N1	Xia dynasty	4,100-3,600 BP			cNW1	
			05DNIT7641H1	N2	Xia dynasty	4,100-3,600 BP			cNW2	
			06DNIIT6306H42	N3	Xia dynasty	4,100-3,600 BP			cNW3	
	Wangchenggang	Dengfeng city, Henan province	T0744H5	WC1	Erligang period	3,550-3,100 BP				
			T0544⑧	WC2	Erligang period	3,550-3,100 BP				
			T0644H13	WC3	Erligang period	3,550-3,100 BP				
			T6566⑦	WC4	Erligang period	3,550-3,100 BP				
			T0244⑥	WC5	Erligang period	3,550-3,100 BP		WCG1	cWCG1	dWCG1
			T264⑨	WC6	Erligang period	3,550-3,100 BP				
			T0544H18	WC7	Erligang period	3,550-3,100 BP				
	Taosi	Xiangfen county, Shanxi province	T5026HG8①	T1	Longshan period	4,350-3,850 BP		TS1	cTS1	dTS1
			T5026⑦	T2	Longshan period	4,350-3,850 BP		TS2	cTS2	dTS2
			T5125HG8③	T3	Longshan period	4,350-3,850 BP				
				T4	Longshan period	4,350-3,850 BP				
			T5126③	T5	Longshan period	4,350-3,850 BP				
			T5026⑦	T6	Longshan period	4,350-3,850 BP	R	TS3	cTS3	dTS3
			T5126H36②	T7	Longshan period	4,350-3,850 BP	R	TS4	cTS4	dTS4
			T5026③	T8	Longshan period	4,350-3,850 BP				
			T5126H34	T9	Longshan period	4,350-3,850 BP				
			T5126H34	T10	Longshan period	4,350-3,850 BP				
	Gaohong	Liulin county, Shanxi province	T14H11C	G1	Shang dynasty	3,500-3,200 BP		GH1	cGH1	dGH1
			T14⑦	G2	Shang dynasty	3,500-3,200 BP	R	GH2	cGH2	dGH2
			T14H11B	G3	Shang dynasty	3,500-3,200 BP				
			T14H11A	G4	Shang dynasty	3,500-3,200 BP	R	GH3	cGH3	dGH3
			T17③	G5	Shang dynasty	3,500-3,200 BP	R	GH4	cGH4	dGH4
			T18②	G6	Shang dynasty	3,500-3,200 BP		GH5	cGH5	dGH5
			T10⑦	G7	Shang dynasty	3,500-3,200 BP		GH6	cGH6	dGH6

Upper Yellow River Region	Lajia	Minhe county, Qinghai province	2000QMLT2711G1:②	L1	Qijia culture	~4,000 BP	R	LJ1	cLJ1	dLJ1
			T1105H78	L2	Qijia culture	~4,000 BP			cLJ2	
			2000QMLT345④	L3	Qijia culture	~4,000 BP		LJ2	cLJ3	dLJ2
			2002QMLT537⑥	L4	Qijia culture	~4,000 BP		LJ3	cLJ4	dLJ3
	Changning	Datong county, Qinghai province	06DCTE1N2	C1	Qijia culture	4,200-3,700 BP	R	CN1	cCN1	dCN1
			06DCTE2N4H52	C2	Qijia culture	4,200-3,700 BP			cCN2	
Middle Yangtze River Region	Qinglongquan	Shiyan city, Hubei province	M231:21	Q1	Middle Shijiahe culture	4,400-4,200 BP				
			M231:23	Q2	Middle Shijiahe culture	4,400-4,200 BP			cQLQ1	
			M231:6	Q3	Middle Shijiahe culture	4,400-4,200 BP		QLQ1	cQLQ2	dQLQ1
			M231:15	Q4	Middle Shijiahe culture	4,400-4,200 BP			cQLQ3	
			M231:4	Q5	Middle Shijiahe culture	4,400-4,200 BP				
			M231:16	Q6	Middle Shijiahe culture	4,400-4,200 BP		QLQ2	cQLQ4	dQLQ2
			M231:22	Q7	Middle Shijiahe culture	4,400-4,200 BP			cQLQ5	
			M231:3	Q8	Middle Shijiahe culture	4,400-4,200 BP	R	QLQ3	cQLQ6	dQLQ3
			M152	Q9	Middle Shijiahe culture	4,400-4,200 BP		QLQ4	cQLQ7	dQLQ4
			M231:2	Q10	Middle Shijiahe culture	4,400-4,200 BP				
			M152	Q11	Middle Shijiahe culture	4,400-4,200 BP			cQLQ8	
			M231:28	Q12	Middle Shijiahe culture	4,400-4,200 BP	R	QLQ5	cQLQ9	dQLQ5
Northeast China	Xinglongwa	Aohan Banner, Inner mongolia	93MAXIF259④:46	X1	Neolithic period (Phase 2)	8,000-7,400 BP		XLW1	cXLW1	dXLW1
			92MAXIT421H337:8	X2	Neolithic period (Phase 2)	8,000-7,400 BP			cXLW2	
			83MAXF6③:3A	X3	Neolithic period (Phase 2)	**Cal. 7,580-7,480 BP**		XLW2	cXLW3	dXLW2
	Wanfabozi	Tonghua city, Jilin province	TW H146:5	WB1	Late Neolithic Period	5,000-4,000 BP	R	WBP1	cWBP1	dWBP1
			T498⑤:8	WB2	Warring state period	~2,500 BP			cWBP2	
			T370⑧:64	WB3	Warring state period	~2,500 BP				
			T368④:1	WB4	Warring state period	~2,500 BP				
	Dashanqian	Harqin Banner, Inner mongolia	T110 H90	D1	Xiajiadian period (lower layer)	4,000-3,500 BP				
			H153④	D2	Xiajiadian period (lower layer)	4,000-3,500 BP	R	DSQ1	cDSQ1	dDSQ1
			T431G2⑦	D3	Xiajiadian period (lower layer)	4,000-3,500 BP	R	DSQ2	cDSQ2	dDSQ2

Note: The CR sequences with green background present the ones published in our previous studies (Larson et al., 2010 and Wang et al., 2012). The "R" in row 'Replication' indicates that the sample was externally replicated at Jilin university. The samples with ‘Cytb sequence’ or ‘CR sequence’ celles filled represent samples from which Cytb or CR sequences could be obtained whereas the ones with blank cells in these rows represent failures to generate Cytb or CR sequences.

Table S2. Primer information.

	Primer name	Sequence(5'-3')	Position*
Control region L-PCR primer	CR-FIP	GATATGTGCTATGTACGATCCCATGCATATAAGCATGTAC	159-410
	CR-BIP	CACGAGCTTAATTACCATGCGAAGAGGGATCCCTGCCAAG	
	CR-F3	CCTGTGTACGTCGTGCATTAAC	
	CR-B3	TCATCAATAGAAACCCCCACG	
Control region PCR primer	CR-F	TGCTAGTCCCCATGCATATAA	181-359
	CR-R	CCTGCCAAGCGGGTTGCTGG	
Cytb gene L-PCR primer	Cytb-FIP	CCTCAGATTCATTCTACGAGGTCTGTTCTGAGGAGCTACGGTCATC	381-675
	Cytb-BIP	TGCCATTCATCATTACCGCCCTTTCCGGTAGGGTTGTTGGA	
	Cytb-F3	CTTCATAGGCTACGTCCTGC	
	Cytb-B3	AGTGTAGTATGGGTGAAATGGAAT	
Cytb gene PCR primer	Cytb-F	CGGAACAGACCTCGTAGAATG	468-609
	Cytb-R	GGTTTCGTGCAGGAATAGGA	

*Positions are referenced to NC_000845.1

Table S3. The *Cytb* gene sequences of 49 ancient pigs.

	1	2	3	4	5	6	7	8	9	10	11	12	13	14	15	16	17	18	19	20	21	22	23	24	25	26	27	28	29	30	31	32	33	34	35	36	37	38	39	40	41	42	43	44	45	46	47	48	49	50
cNZT1	A	A	T	C	T	G	A	G	G	G	G	G	C	T	T	T	T	C	C	G	T	C	G	A	C	A	A	A	G	C	A	A	C	C	C	T	C	A	C	A	C	G	A	T	T	C	T	T	C	G
cNZT2	.	.	.	.	.	.	.	.	.	.	.	.	.	.	.	.	.	.	.	.	.	.	.	.	.	.	.	.	.	.	.	.	.	.	.	.	.	.	.	.	.	.	.	.	.	.	.	.	.	.
cNZT3	.	.	.	.	.	.	.	.	.	.	.	.	.	.	.	.	.	.	.	.	.	.	.	.	.	.	.	.	.	.	.	.	.	.	.	.	.	.	.	.	.	.	.	.	.	.	.	.	.	.
cJH1	.	.	.	.	.	.	.	.	.	.	.	.	.	.	.	.	.	.	.	.	.	.	.	.	.	.	.	.	.	.	.	.	.	.	.	.	.	.	.	.	.	.	.	.	.	.	.	.	.	.
cJH2	.	.	.	.	.	.	.	.	.	.	.	.	.	.	.	.	.	.	.	.	.	.	.	.	.	.	.	.	.	.	.	.	.	.	.	.	.	.	.	.	.	.	.	.	.	.	.	.	.	.
cCS1	.	.	.	.	.	.	.	.	.	.	.	.	.	.	.	.	.	.	.	.	.	.	.	.	.	.	.	.	.	.	.	.	.	.	.	.	.	.	.	.	.	.	.	.	.	.	.	.	.	.
cCS2	.	.	.	.	.	.	.	.	.	.	.	.	.	.	.	.	.	.	.	.	.	.	.	.	.	.	.	.	.	.	.	.	.	.	.	.	.	.	.	.	.	.	.	.	.	.	.	.	.	.
cCS3	.	.	.	.	.	.	.	.	.	.	.	.	.	.	.	.	.	.	.	.	.	.	.	.	.	.	.	.	.	.	.	.	.	.	.	.	.	.	.	.	.	.	.	.	.	.	.	.	.	.
cWD1	.	.	.	.	.	.	.	.	.	.	.	.	.	.	.	.	.	.	.	.	.	.	.	.	.	.	.	.	.	.	.	.	.	.	.	.	.	.	.	.	.	.	.	.	.	.	.	.	.	.
cWD2	.	.	.	.	.	.	.	.	.	.	.	.	.	.	.	.	.	.	.	.	.	.	.	.	.	.	.	.	.	.	.	.	.	.	.	.	.	.	.	.	.	.	.	.	.	.	.	.	.	.
cWD3	.	.	.	.	.	.	.	.	.	.	.	.	.	.	.	.	.	.	.	.	.	.	.	.	.	.	.	.	.	.	.	.	.	.	.	.	.	.	.	.	.	.	.	.	.	.	.	.	.	.
cGCJ1	.	.	.	.	.	.	.	.	.	.	.	.	.	.	.	.	.	.	.	.	.	.	.	.	.	.	.	.	.	.	.	.	.	.	.	.	.	.	.	.	.	.	.	.	.	.	.	.	.	.
cGCJ2	.	.	.	.	.	.	.	.	.	.	.	.	.	.	.	.	.	.	.	.	.	.	.	.	.	.	.	.	.	.	.	.	.	.	.	.	.	.	.	.	.	.	.	.	.	.	.	.	.	.
cNW1	.	.	.	.	.	.	.	.	.	.	.	.	.	.	.	.	.	.	.	.	.	.	.	.	.	.	.	.	.	.	.	.	.	.	.	.	.	.	.	.	.	.	.	.	.	.	.	.	.	.
cNW2	.	.	.	.	.	.	.	.	.	.	.	.	.	.	.	.	.	.	.	.	.	.	.	.	.	.	.	.	.	.	.	.	.	.	.	.	.	.	.	.	.	.	.	.	.	.	.	.	.	.
cNW3	.	.	.	.	.	.	.	.	.	.	.	.	.	.	.	.	.	.	.	.	.	.	.	.	.	.	.	.	.	.	.	.	.	.	.	.	.	.	.	.	.	.	.	.	.	.	.	.	.	.
cWCG1	.	.	.	.	.	.	.	.	.	.	.	.	.	.	.	.	.	.	.	.	.	.	.	.	.	.	.	.	.	.	.	.	.	.	.	.	.	.	.	.	.	.	.	.	.	.	.	.	.	.
cTS1	.	.	.	.	.	.	.	.	.	.	.	.	.	.	.	.	.	.	.	.	.	.	.	.	.	.	.	.	.	.	.	.	.	.	.	.	.	.	.	.	.	.	.	.	.	.	.	.	.	.
cTS2	.	.	.	.	.	.	.	.	.	.	.	.	.	.	.	.	.	.	.	.	.	.	.	.	.	.	.	.	.	.	.	.	.	.	.	.	.	.	.	.	.	.	.	.	.	.	.	.	.	.
cTS3	.	.	.	.	.	.	.	.	.	.	.	.	.	.	.	.	.	.	.	.	.	.	.	.	.	.	.	.	.	.	.	.	.	.	.	.	.	.	.	.	.	.	.	.	.	.	.	.	.	.
cTS4	.	.	.	.	.	.	.	.	.	.	.	.	.	.	.	.	.	.	.	.	.	.	.	.	.	.	.	.	.	.	.	.	.	.	.	.	.	.	.	.	.	.	.	.	.	.	.	.	.	.
cGH1	.	.	.	.	.	.	.	.	.	.	.	.	.	.	.	.	.	.	.	.	.	.	.	.	.	.	.	.	.	.	.	.	.	.	.	.	.	.	.	.	.	.	.	.	.	.	.	.	.	.
cGH2	.	.	.	.	.	.	.	.	.	.	.	.	.	.	.	.	.	.	.	.	.	.	.	.	.	.	.	.	.	.	.	.	.	.	.	.	.	.	.	.	.	.	.	.	.	.	.	.	.	.
cGH3	.	.	.	.	.	.	.	.	.	.	.	.	.	.	.	.	.	.	.	.	.	.	.	.	.	.	.	.	.	.	.	.	.	.	.	.	.	.	.	.	.	.	.	.	.	.	.	.	.	.
cGH4	.	.	.	.	.	.	.	.	.	.	.	.	.	.	.	.	.	.	.	.	.	.	.	.	.	.	.	.	.	.	.	.	.	.	.	.	.	.	.	.	.	.	.	.	.	.	.	.	.	.
cGH5	.	.	.	.	.	.	.	.	.	.	.	.	.	.	.	.	.	.	.	.	.	.	.	.	.	.	.	.	.	.	.	.	.	.	.	.	.	.	.	.	.	.	.	.	.	.	.	.	.	.
cGH6	.	.	.	.	.	.	.	.	.	.	.	.	.	.	.	.	.	.	.	.	.	.	.	.	.	.	.	.	.	.	.	.	.	.	.	.	.	.	.	.	.	.	.	.	.	.	.	.	.	.
cLJ1	.	.	.	.	.	.	.	.	.	.	.	.	.	.	.	.	.	.	.	.	.	.	.	.	.	.	.	.	.	.	.	.	.	.	.	.	.	.	.	.	.	.	.	.	.	.	.	.	.	.
cLJ2	.	.	.	.	.	.	.	.	.	.	.	.	.	.	.	.	.	.	.	.	.	.	.	.	.	.	.	.	.	.	.	.	.	.	.	.	.	.	.	.	.	.	.	.	.	.	.	.	.	.
cLJ3	.	.	.	.	.	.	.	.	.	.	.	.	.	.	.	.	.	.	.	.	.	.	.	.	.	.	.	.	.	.	.	.	.	.	.	.	.	.	.	.	.	.	.	.	.	.	.	.	.	.
cLJ4	.	.	.	.	.	.	.	.	.	.	.	.	.	.	.	.	.	.	.	.	.	.	.	.	.	.	.	.	.	.	.	.	.	.	.	.	.	.	.	.	.	.	.	.	.	.	.	.	.	.
cCN1	.	.	.	.	.	.	.	.	.	.	.	.	.	.	.	.	.	.	.	.	.	.	.	.	.	.	.	.	.	.	.	.	.	.	.	.	.	.	.	.	.	.	.	.	.	.	.	.	.	.
cCN2	.	.	.	.	.	.	.	.	.	.	.	.	.	.	.	.	.	.	.	.	.	.	.	.	.	.	.	.	.	.	.	.	.	.	.	.	.	.	.	.	.	.	.	.	.	.	.	.	.	.
cQLQ1	.	.	.	.	.	.	.	.	.	.	.	.	.	.	.	.	.	.	.	.	.	.	.	.	.	.	.	.	.	.	.	.	.	.	.	.	.	.	.	.	.	.	.	.	.	.	.	.	.	.
cQLQ2	.	.	.	.	.	.	.	.	.	.	.	.	.	.	.	.	.	.	.	.	.	.	.	.	.	.	.	.	.	.	.	.	.	.	.	.	.	.	.	.	.	.	.	.	.	.	.	.	.	.
cQLQ3	.	.	.	.	.	.	.	.	.	.	.	.	.	.	.	.	.	.	.	.	.	.	.	.	.	.	.	.	.	.	.	.	.	.	.	.	.	.	.	.	.	.	.	.	.	.	.	.	.	.
cQLQ4	.	.	.	.	.	.	.	.	.	.	.	.	.	.	.	.	.	.	.	.	.	.	.	.	.	.	.	.	.	.	.	.	.	.	.	.	.	.	.	.	.	.	.	.	.	.	.	.	.	.
cQLQ5	.	.	.	.	.	.	.	.	.	.	.	.	.	.	.	.	.	.	.	.	.	.	.	.	.	.	.	.	.	.	.	.	.	.	.	.	.	.	.	.	.	.	.	.	.	.	.	.	.	.
cQLQ6	.	.	.	.	.	.	.	.	.	.	.	.	.	.	.	.	.	.	.	.	.	.	.	.	.	.	.	.	.	.	.	.	.	.	.	.	.	.	.	.	.	.	.	.	.	.	.	.	.	.
cQLQ7	.	.	.	.	.	.	.	.	.	.	.	.	.	.	.	.	.	.	.	.	.	.	.	.	.	.	.	.	.	.	.	.	.	.	.	.	.	.	.	.	.	.	.	.	.	.	.	.	.	.
cQLQ8	.	.	.	.	.	.	.	.	.	.	.	.	.	.	.	.	.	.	.	.	.	.	.	.	.	.	.	.	.	.	.	.	.	.	.	.	.	.	.	.	.	.	.	.	.	.	.	.	.	.
cQLQ9	.	.	.	.	.	.	.	.	.	.	.	.	.	.	.	.	.	.	.	.	.	.	.	.	.	.	.	.	.	.	.	.	.	.	.	.	.	.	.	.	.	.	.	.	.	.	.	.	.	.
cXLW1	.	.	.	.	.	.	.	.	.	.	.	.	.	.	.	.	.	.	.	.	.	.	.	.	.	.	.	.	.	.	.	.	.	.	.	.	.	.	.	.	.	.	.	.	.	.	.	.	.	.
cXLW2	.	.	.	.	.	.	.	.	.	.	.	.	.	.	.	.	.	.	.	.	.	.	.	.	.	.	.	.	.	.	.	.	.	.	.	.	.	.	.	.	.	.	.	.	.	.	.	.	.	.
cXLW3	.	.	.	.	.	.	.	.	.	.	.	.	.	.	.	.	.	.	.	.	.	.	.	.	.	.	.	.	.	.	.	.	.	.	.	.	.	.	.	.	.	.	.	.	.	.	.	.	.	.
cWBP1	.	.	.	.	.	.	.	.	.	.	.	.	.	.	.	.	.	.	.	.	.	.	.	.	.	.	.	.	.	.	.	.	.	.	.	.	.	.	.	.	.	.	.	.	.	.	.	.	.	.
cWBP2	.	.	.	.	.	.	.	.	.	.	.	.	.	.	.	.	.	.	.	.	.	.	.	.	.	.	.	.	.	.	.	.	.	.	.	.	.	.	.	.	.	.	.	.	.	.	.	.	.	.
cDSQ1	.	.	.	.	.	.	.	.	.	.	.	.	.	.	.	.	.	.	.	.	.	.	.	.	.	.	.	.	.	.	.	.	.	.	.	.	.	.	.	.	.	.	.	.	.	.	.	.	.	.
cDSQ2	.	.	.	.	.	.	.	.	.	.	.	.	.	.	.	.	.	.	.	.	.	.	.	.	.	.	.	.	.	.	.	.	.	.	.	.	.	.	.	.	.	.	.	.	.	.	.	.	.	.

	51	52	53	54	55	56	57	58	59	60	61	62	63	64	65	66	67	68	69	70	71	72	73	74	75	76	77	78	79	80	81	82	83	84	85	86	87	88	89	90	91	92	93	94	95	96	97	98	99	100	101
cNZT1	C	C	T	T	T	C	A	C	T	T	T	A	T	C	C	T	G	C	C	A	T	T	C	A	T	C	A	T	T	A	C	C	G	C	C	C	T	C	G	C	A	G	C	C	G	T	A	C	A	T	C
cNZT2	.	.	.	.	.	.	.	.	.	.	.	.	.	.	.	.	.	.	.	.	.	.	.	.	.	.	.	.	.	.	.	.	.	.	.	.	.	.	.	.	.	.	.	.	.	.	.	.	.	.	.
cNZT3	.	.	.	.	.	.	.	.	.	.	.	.	.	.	.	.	.	.	.	.	.	.	.	.	.	.	.	.	.	.	.	.	.	.	.	.	.	.	.	.	.	.	.	.	.	.	.	.	.	.	.
cJH1	.	.	.	.	.	.	.	.	.	.	.	.	.	.	.	.	.	.	.	.	.	.	.	.	.	.	.	.	.	.	.	.	.	.	.	.	.	.	.	.	.	.	.	.	.	.	.	.	.	.	.
cJH2	.	.	.	.	.	.	.	.	.	.	.	.	.	.	.	.	.	.	.	.	.	.	.	.	.	.	.	.	.	.	.	.	.	.	.	.	.	.	.	.	.	.	.	.	.	.	.	.	.	.	.
cCS1	.	.	.	.	.	.	.	.	.	.	.	.	.	.	.	.	.	.	.	.	.	.	.	.	.	.	.	.	.	.	.	.	.	.	.	.	.	.	.	.	.	.	.	.	.	.	.	.	.	.	.
cCS2	.	.	.	.	.	.	.	.	.	.	.	.	.	.	.	.	.	.	.	.	.	.	.	.	.	.	.	.	.	.	.	.	.	.	.	.	.	.	.	.	.	.	.	.	.	.	.	.	.	.	.
cCS3	.	.	.	.	.	.	.	.	.	.	.	.	.	.	.	.	.	.	.	.	.	.	.	.	.	.	.	.	.	.	.	.	.	.	.	.	.	.	.	.	.	.	.	.	.	.	.	.	.	.	.
cWD1	.	.	.	.	.	.	.	.	.	.	.	.	.	.	.	.	.	.	.	.	.	.	.	.	.	.	.	.	.	.	.	.	.	.	.	.	.	.	.	.	.	.	.	.	.	.	.	.	.	.	.
cWD2	.	.	.	.	.	.	.	.	.	.	.	.	.	.	.	.	.	.	.	.	.	.	.	.	.	.	.	.	.	.	.	.	.	.	.	.	.	.	.	.	.	.	.	.	.	.	.	.	.	.	.
cWD3	.	.	.	.	.	.	.	.	.	.	.	.	.	.	.	.	.	.	.	.	.	.	.	.	.	.	.	.	.	.	.	.	.	.	.	.	.	.	.	.	.	.	.	.	.	.	.	.	.	.	.
cGCJ1	.	.	.	.	.	.	.	.	.	.	.	.	.	.	.	.	.	.	.	.	.	.	.	.	.	.	.	.	.	.	.	.	.	.	.	.	.	.	.	.	.	.	.	.	.	.	.	.	.	.	.
cGCJ2	.	.	.	.	.	.	.	.	.	.	.	.	.	.	.	.	.	.	.	.	.	.	.	.	.	.	.	.	.	.	.	.	.	.	.	.	.	.	.	.	.	.	.	.	.	.	.	.	.	.	.
cNW1	.	.	.	.	.	.	.	.	.	.	.	.	.	.	.	.	.	.	.	.	.	.	.	.	.	.	.	.	.	.	.	.	.	.	.	.	.	.	.	.	.	.	.	.	.	.	.	.	.	.	.
cNW2	.	.	.	.	.	.	.	.	.	.	.	.	.	.	.	.	.	.	.	.	.	.	.	.	.	.	.	.	.	.	.	.	.	.	.	.	.	.	.	.	.	.	.	.	.	.	.	.	.	.	.
cNW3	.	.	.	.	.	.	.	.	.	.	.	.	.	.	.	.	.	.	.	.	.	.	.	.	.	.	.	.	.	.	.	.	.	.	.	.	.	.	.	.	.	A	.	.	.	.	.	.	.	.	.
cWCG1	.	.	.	.	.	.	.	.	.	.	.	.	.	.	.	.	.	.	.	.	.	.	.	.	.	.	.	.	.	.	.	.	.	.	.	.	.	.	.	.	.	.	.	.	.	.	.	.	.	.	.
cTS1	.	.	.	.	.	.	.	.	.	.	.	.	.	.	.	.	.	.	.	.	.	.	.	.	.	.	.	.	.	.	.	.	.	.	.	.	.	.	.	.	.	.	.	.	.	.	.	.	.	.	.
cTS2	.	.	.	.	.	.	.	.	.	.	.	.	.	.	.	.	.	.	.	.	.	.	.	.	.	.	.	.	.	.	.	.	.	.	.	.	.	.	.	.	.	.	.	.	.	.	.	.	.	.	.
cTS3	.	.	.	.	.	.	.	.	.	.	.	.	.	.	.	.	.	.	.	.	.	.	.	.	.	.	.	.	.	.	.	.	.	.	.	.	.	.	.	.	.	.	.	.	.	.	.	.	.	.	.
cTS4	.	.	.	.	.	.	.	.	.	.	.	.	.	.	.	.	.	.	.	.	.	.	.	.	.	.	.	.	.	.	.	.	.	.	.	.	.	.	.	.	.	.	.	.	.	.	.	.	.	.	.
cGH1	.	.	.	.	.	.	.	.	.	.	.	.	.	.	.	.	.	.	.	.	.	.	.	.	.	.	.	.	.	.	.	.	.	.	.	.	.	.	.	.	.	.	.	.	.	.	.	.	.	.	.
cGH2	.	.	.	.	.	.	.	.	.	.	.	.	.	.	.	.	.	.	.	.	.	.	.	.	.	.	.	.	.	.	.	.	.	.	.	.	.	.	.	.	.	.	.	.	.	.	.	.	.	.	.
cGH3	.	.	.	.	.	.	.	.	.	.	.	.	.	.	.	.	.	.	.	.	.	.	.	.	.	.	.	.	.	.	.	.	.	.	.	.	.	.	.	.	.	.	.	.	.	.	.	.	.	.	.
cGH4	.	.	.	.	.	.	.	.	.	.	.	.	.	.	.	.	.	.	.	.	.	.	.	.	.	.	.	.	.	.	.	.	.	.	.	.	.	.	.	.	.	.	.	.	.	.	.	.	.	.	.
cGH5	.	.	.	.	.	.	.	.	.	.	.	.	.	.	.	.	.	.	.	.	.	.	.	.	.	.	.	.	.	.	.	.	.	.	.	.	.	.	.	.	.	.	.	.	.	.	.	.	.	.	.
cGH6	.	.	.	.	.	.	.	.	.	.	.	.	.	.	.	.	.	.	.	.	.	.	.	.	.	.	.	.	.	.	.	.	.	.	.	.	.	.	.	.	.	.	.	.	.	.	.	.	.	.	.
cLJ1	.	.	.	.	.	.	.	.	.	.	.	.	.	.	.	.	.	.	.	.	.	.	.	.	.	.	.	.	.	.	.	.	.	.	.	.	.	.	.	.	.	.	.	.	.	.	.	.	.	.	.
cLJ2	.	.	.	.	.	.	.	.	.	.	.	.	.	.	.	.	.	.	.	.	.	.	.	.	.	.	.	.	.	.	.	.	.	.	.	.	.	.	.	.	.	.	.	.	.	.	.	.	.	.	.
cLJ3	.	.	.	.	.	.	.	.	.	.	.	.	.	.	.	.	.	.	.	.	.	.	.	.	.	.	.	.	.	.	.	.	.	.	.	.	.	.	.	.	.	.	.	.	.	.	.	.	.	.	.
cLJ4	.	.	.	.	.	.	.	.	.	.	.	.	.	.	.	.	.	.	.	.	.	.	.	.	.	.	.	.	.	.	.	.	.	.	.	.	.	.	.	.	.	.	.	.	.	.	.	.	.	.	.
cCN1	.	.	.	.	.	.	.	.	.	.	.	.	.	.	.	.	.	.	.	.	.	.	.	.	.	.	.	.	.	.	.	.	.	.	.	.	.	.	.	.	.	.	.	.	.	.	.	.	.	.	.
cCN2	.	.	.	.	.	.	.	.	.	.	.	.	.	.	.	.	.	.	.	.	.	.	.	.	.	.	.	.	.	.	.	.	.	.	.	.	.	.	.	.	.	.	.	.	.	.	.	.	.	.	.
cQLQ1	.	.	.	.	.	.	.	.	.	.	.	.	.	.	.	.	.	.	.	.	.	.	.	.	.	.	.	.	.	.	.	.	.	.	.	.	.	.	.	.	.	.	.	.	.	.	.	.	.	.	.
cQLQ2	.	.	.	.	.	.	.	.	.	.	.	.	.	.	.	.	.	.	.	.	.	.	.	.	.	.	.	.	.	.	.	.	.	.	.	.	.	.	.	.	.	.	.	.	.	.	.	.	.	.	.
cQLQ3	.	.	.	.	.	.	.	.	.	.	.	.	.	.	.	.	.	.	.	.	.	.	.	.	.	.	.	.	.	.	.	.	.	.	.	.	.	.	.	.	.	.	.	.	.	.	.	.	.	.	.
cQLQ4	.	.	.	.	C	.	.	.	.	.	.	.	.	.	.	.	.	.	.	.	.	.	.	.	.	.	.	.	.	.	.	.	.	.	.	.	.	.	.	.	.	.	.	.	.	.	.	.	.	.	.
cQLQ5	.	.	.	.	C	.	.	.	.	.	.	.	.	.	.	.	.	.	.	.	.	.	.	.	.	.	.	.	.	.	.	.	.	.	.	.	.	.	.	.	.	.	.	.	.	.	.	.	.	.	.
cQLQ6	.	.	.	.	C	.	.	.	.	.	.	.	.	.	.	.	.	.	.	.	.	.	.	.	.	.	.	.	.	.	.	.	.	.	.	.	.	.	.	.	.	.	.	.	.	.	.	.	.	.	.
cQLQ7	.	.	.	.	.	.	.	.	.	.	.	.	.	.	.	.	.	.	.	.	.	.	.	.	.	.	.	.	.	.	.	.	.	.	.	.	.	.	.	.	.	.	.	.	.	.	.	.	.	.	.
cQLQ8	.	.	.	.	.	.	.	.	.	.	.	.	.	.	.	.	.	.	.	.	.	.	.	.	.	.	.	.	.	.	.	A	.	.	.	.	.	.	.	.	.
cQLQ9	.	.	.	.	.	.	.	.	.	.	.	.	.	.	.	.	.	.	.	.	.	.	.	.	.	.	.	.	.	.	.	.	.	.	.	.	.	.	.	.	.	A	.	.	.	.	.	.	.	.	.
cXLW1	.	.	.	.	.	.	.	.	.	.	.	.	.	.	.	.	.	.	.	.	.	.	.	.	.	.	.	.	.	.	.	.	.	.	.	.	.	.	.	.	.	.	.	.	.	.	.	.	.	.	.
cXLW2	.	.	.	.	.	.	.	.	.	.	.	.	.	.	.	.	.	.	.	.	.	.	.	.	.	.	.	.	.	.	.	.	.	.	.	.	.	.	.	.	.	.	.	.	.	.	.	.	.	.	.
cXLW3	.	.	.	.	.	.	.	.	.	.	.	.	.	.	.	.	.	.	.	.	.	.	.	.	.	.	.	.	.	.	.	.	.	.	.	.	.	.	.	.	.	.	.	.	.	.	.	.	.	.	.
cWBP1	.	.	.	.	.	.	.	.	.	.	.	.	.	.	.	.	.	.	.	.	.	.	.	G	.	.	.	.	.	.	.	.	.	.	.	.	.	.	.	.	.	.	.	.	.	.	.	.	.	.	.
cWBP2	.	.	.	.	C	.	.	.	.	.	.	.	.	.	.	.	.	.	.	.	.	.	.	.	.	.	.	.	.	.	.	.	.	.	.	.	.	.	.	.	.	.	.	.	.	.	.	.	.	.	.
cDSQ1	.	.	.	.	.	.	.	.	.	.	.	.	.	.	.	.	.	.	.	.	.	.	.	.	.	.	.	.	.	.	.	.	.	.	.	.	.	.	.	.	.	.	.	.	.	.	.	.	.	.	.
cDSQ2	.	.	.	.	.	.	.	.	.	.	.	.	.	.	.	.	.	.	.	.	.	.	.	.	.	.	.	.	.	.	.	.	.	.	.	.	.	.	.	.	.	.	.	.	.	.	.	.	.	.	.

Table S4. The 38 ancient pig control region sequences.

	1	2	3	4	5	6	7	8	9	10	11	12	13	14	15	16	17	18	19	20	21	22	23	24	25	26	27	28	29	30	31	32	33	34	35	36	37	38	39	40	41	42	43	44	45	46	47	48	49	50	51	52	53	54	55	56	57	58	59	60	61	62	63	64	65	66	67	68	69	70
dNZT1	G	C	A	T	G	T	A	C	A	T	A	T	T	A	T	T	A	T	T	A	A	T	A	T	T	A	C	A	T	A	G	T	A	C	A	T	A	T	C	A	T	T	A	T	T	G	A	T	C	G	T	A	C	A	T	A	G	C	A	C	A	T	A	T	C	A	T	G	T	C
dNZT2	.	.	.	.	.	.	.	.	.	.	.	.	.	.	.	.	.	.	.	.	.	.	.	.	.	.	.	.	.	.	.	.	.	.	.	.	.	.	.	.	.	.	.	.	.	.	.	.	.	.	.	.	.	.	.	.	.	.	.	.	.	.	.	.	.	.	.	.	.	.
dNZT3	.	.	.	.	.	.	.	.	.	.	.	.	.	.	.	.	.	.	.	.	.	.	.	.	.	.	.	.	.	.	.	.	.	.	.	.	.	.	.	.	.	.	.	.	.	.	.	.	.	.	.	.	.	.	.	.	.	.	.	.	.	.	.	.	.	.	.	.	.	.
dNZT4	.	.	.	.	.	.	.	.	.	.	.	.	.	.	.	.	.	.	.	.	.	.	.	.	.	.	.	.	.	.	.	.	.	.	.	.	.	.	T	.	.	.	.	.	.	.	.	.	.	.	.	.	.	.	.	.	.	.	.	.	.	.	.	.	.	.	.	.	.	.
dJH1	.	.	.	.	.	.	.	.	.	.	.	.	.	.	.	.	.	.	.	.	.	.	.	.	.	.	.	.	.	.	.	.	.	.	.	.	.	.	T	.	.	.	.	.	.	.	.	.	.	.	.	.	.	.	.	.	.	.	.	.	.	.	.	.	.	.	.	.	.	.
dJH2	.	.	.	.	.	.	.	.	.	.	.	.	.	.	.	.	.	.	.	.	.	.	.	.	.	.	.	.	.	.	.	.	.	.	.	.	.	.	T	.	.	.	.	.	.	.	.	.	.	.	.	.	.	.	.	.	.	.	.	.	.	.	.	.	.	.	.	.	.	.
dCS1	.	.	.	.	.	.	.	.	.	.	.	.	.	.	.	.	.	.	.	.	.	.	.	.	.	.	.	.	.	.	.	.	.	.	.	.	.	.	.	.	.	.	.	.	.	.	.	.	.	.	.	.	.	.	.	.	.	.	.	.	.	.	.	.	.	.	.	.	.	.
dCS2	.	.	.	.	.	.	.	.	.	.	.	.	.	.	.	.	.	.	.	.	.	.	.	.	.	.	.	.	.	.	.	.	.	.	.	.	.	.	T	.	.	.	.	.	.	.	.	.	.	.	.	.	.	.	.	.	.	.	.	.	.	.	.	.	.	.	.	.	.	.
dTS1	.	.	.	.	.	.	.	.	.	.	.	.	.	.	.	.	.	.	.	.	.	.	.	.	.	.	.	.	.	.	.	.	.	.	.	.	.	.	.	.	.	.	.	.	.	.	.	.	.	.	.	.	.	.	.	.	.	.	.	.	.	.	.	.	.	.	.	.	.	.
dTS2	.	.	.	.	.	.	.	.	.	.	.	.	.	.	.	.	.	.	.	.	.	.	.	.	.	.	.	.	.	.	.	.	.	.	.	.	.	.	.	.	.	.	.	.	.	.	.	.	.	.	.	.	.	.	.	.	.	.	.	.	.	.	.	.	.	.	.	.	.	.
dTS3	.	.	.	.	.	.	.	.	.	.	.	.	.	.	.	.	.	.	.	.	.	.	.	.	.	.	.	.	.	.	.	.	.	.	.	.	.	.	.	.	.	.	.	.	.	.	.	.	.	.	.	.	.	.	.	.	.	.	.	.	.	.	.	.	.	.	.	.	.	.
dTS4	.	.	.	.	.	.	.	.	.	.	.	.	.	.	.	.	.	.	.	.	.	.	.	.	.	.	.	.	.	.	.	.	.	.	.	.	.	.	.	.	.	.	.	.	.	.	.	.	.	.	.	.	.	.	.	.	.	.	.	.	.	.	.	.	.	.	.	.	.	.
dWD1	.	.	.	.	.	.	.	.	.	.	.	.	.	.	.	.	.	.	.	.	.	.	.	.	.	.	.	.	.	.	.	.	.	.	.	.	.	.	.	.	.	.	.	.	.	.	.	.	.	.	.	.	.	.	.	.	.	.	.	.	.	.	.	.	T	.	.	.	.	.
dWD2	.	.	.	.	.	.	.	.	.	.	.	.	.	.	.	.	.	.	.	.	.	.	.	.	.	.	.	.	.	.	.	.	.	.	.	.	.	.	T	.	.	.	.	.	.	.	.	.	.	.	.	.	.	.	.	.	.	.	.	.	.	.	.	.	.	.	.	.	.	.
dWD3	.	.	.	.	.	.	.	.	.	.	.	.	.	.	.	.	.	.	.	.	.	.	.	.	.	.	.	.	.	.	.	.	.	.	.	.	.	.	.	.	.	.	.	.	.	.	.	.	.	.	.	.	.	.	.	.	.	.	.	.	.	.	.	.	.	.	.	.	.	.
dGCJ1	.	.	.	.	.	.	.	.	.	.	.	.	.	.	.	.	.	.	.	.	.	.	.	.	.	.	.	.	.	.	.	.	.	.	.	.	.	.	.	.	.	.	.	.	.	.	.	.	.	.	.	.	.	.	.	.	.	.	.	.	.	.	.	.	.	.	.	.	.	.
dGCJ2	.	.	.	.	.	.	.	.	.	.	.	.	.	.	.	.	.	.	.	.	.	.	.	.	.	.	.	.	.	.	.	.	.	.	.	.	.	.	.	.	.	.	.	.	.	.	.	.	.	.	.	.	.	.	.	.	.	.	.	.	.	.	.	.	.	.	.	.	.	.
dGH1	.	.	.	.	.	.	.	.	.	.	.	.	.	.	.	.	.	.	.	.	.	.	.	.	.	.	.	.	.	.	.	.	.	.	.	.	.	.	.	.	.	.	.	.	.	.	.	.	.	.	.	.	.	.	.	.	.	.	.	.	.	.	.	.	.	.	.	.	.	.
dGH2	.	.	.	.	.	.	.	.	.	.	.	.	.	.	.	.	.	.	.	.	.	.	.	.	.	.	.	.	.	.	.	.	.	.	.	.	.	.	T	.	.	.	.	.	.	.	.	.	.	.	.	.	.	.	.	.	.	.	.	.	.	.	.	.	.	.	.	.	.	.
dGH3	.	.	.	.	.	.	.	.	.	.	.	.	.	.	.	.	.	.	.	.	.	.	.	.	.	.	.	.	.	.	.	.	.	.	.	.	.	.	.	.	.	.	.	.	.	.	.	.	.	.	.	.	.	.	.	.	.	.	.	.	.	.	.	.	.	.	.	.	.	.
dGH4	.	.	.	.	.	.	.	.	.	.	.	.	.	.	.	.	.	.	.	.	.	.	.	.	.	.	.	.	.	.	.	.	.	.	.	.	.	.	T	.	.	.	.	.	.	.	.	.	.	.	.	.	.	.	.	.	.	.	.	.	.	.	.	.	.	.	.	.	.	.
dGH5	.	.	.	.	.	.	.	.	.	.	.	.	.	.	.	.	.	.	.	.	.	.	.	.	.	.	.	.	.	.	.	.	.	.	.	.	.	.	.	.	.	.	.	.	.	.	.	.	.	.	.	.	.	.	.	.	.	.	.	.	.	.	.	.	.	.	.	.	.	.
dGH6	.	.	.	.	.	.	.	.	.	.	.	.	.	.	.	.	.	.	.	.	.	.	.	.	.	.	.	.	.	.	.	.	.	.	.	.	.	.	.	.	.	.	.	.	.	.	.	.	.	.	.	.	.	.	.	.	.	.	.	.	.	.	.	.	.	.	.	.	.	.
dWCG1	.	.	.	.	.	.	.	.	.	.	.	.	.	.	.	.	.	.	.	.	.	.	.	.	.	.	.	.	.	.	.	.	.	.	.	.	.	.	.	.	.	.	.	.	.	.	.	.	.	.	.	.	.	.	.	.	.	.	.	.	.	.	.	.	.	.	.	.	.	.
dXLW1	.	.	.	.	.	.	.	.	.	.	.	.	.	.	.	.	.	.	.	.	.	.	.	.	.	.	.	.	.	.	.	.	.	.	.	.	.	.	T	.	.	.	.	.	.	.	.	.	.	.	.	.	.	.	.	.	.	.	.	.	.	.	.	.	.	.	.	.	.	.
dXLW2	.	.	.	.	.	.	.	.	.	.	.	.	.	.	.	.	.	.	.	.	.	.	.	.	.	.	.	.	.	.	.	.	.	.	.	.	.	.	T	.	.	.	.	.	.	.	.	.	.	.	.	.	.	.	.	.	.	.	.	.	.	.	.	.	.	.	.	.	.	.
dWBP1	.	.	.	.	.	.	.	.	.	.	.	.	.	.	.	.	.	.	.	.	.	.	.	.	.	.	.	.	.	.	.	.	.	.	.	.	.	.	.	.	.	.	.	.	.	.	.	.	.	.	.	.	.	.	.	.	.	.	.	.	.	.	.	.	.	.	.	.	.	.
dDSQ1	.	.	.	.	.	.	.	.	.	.	.	.	.	.	.	.	.	.	.	.	.	.	.	.	.	.	.	.	.	.	.	.	.	.	.	.	.	.	.	.	.	.	.	.	.	.	.	.	.	.	.	.	.	.	.	.	.	.	.	.	.	.	.	.	.	.	.	.	.	.
dDSQ2	.	.	.	.	.	.	.	.	.	.	.	.	.	.	.	.	.	.	.	.	.	.	.	.	.	.	.	.	.	.	.	.	.	.	.	.	.	.	.	.	.	.	.	.	.	.	.	.	.	.	.	.	.	.	.	.	.	.	.	.	.	.	.	.	.	.	.	.	.	.
dCN1	.	.	.	.	.	.	.	.	.	.	.	.	.	.	.	.	.	.	.	.	.	.	.	.	.	.	.	.	.	.	.	.	.	.	.	.	.	.	T	.	.	.	.	.	.	.	.	.	.	.	.	.	.	.	.	.	.	.	.	.	.	.	.	.	.	.	.	.	.	.
dLJ1	.	.	.	.	.	.	.	.	.	.	.	.	.	.	.	.	.	.	.	.	.	.	.	.	.	.	.	.	.	.	.	.	.	.	.	.	.	.	.	.	.	.	.	.	.	.	.	.	.	.	.	.	.	.	.	.	.	.	.	.	.	.	.	.	.	.	.	.	.	.
dLJ2	.	.	.	.	.	.	.	.	.	.	.	.	.	.	.	.	.	.	.	.	.	.	.	.	.	.	.	.	.	.	.	.	.	.	.	.	.	.	.	.	.	.	.	.	.	.	.	.	.	.	.	.	.	.	.	.	.	.	.	.	.	.	.	.	.	.	.	.	.	.
dLJ3	.	.	.	.	.	.	.	.	.	.	.	.	.	.	.	.	.	.	.	.	.	.	.	.	.	.	.	.	.	.	.	.	.	.	.	.	.	.	.	.	.	.	.	.	.	.	.	.	.	.	.	.	.	.	.	.	.	.	.	.	.	.	.	.	.	.	.	.	.	.
dQLQ1	.	.	.	.	.	.	.	.	.	.	.	.	.	.	.	.	.	.	.	.	.	.	.	.	.	.	.	.	.	.	.	.	.	.	.	.	.	.	T	.	.	.	.	.	.	.	.	.	.	.	.	.	.	.	.	.	.	.	.	.	.	.	.	.	.	.	.	.	.	.
dQLQ2	.	.	.	.	.	.	.	.	.	.	.	.	.	.	.	.	.	.	.	.	.	.	.	.	.	.	.	.	.	.	.	.	.	.	.	.	.	.	T	.	.	.	.	.	.	.	.	.	.	.	.	.	.	.	.	.	.	.	.	.	.	.	.	.	.	.	.	.	.	.
dQLQ3	.	.	.	.	.	.	.	.	.	.	.	.	.	.	.	.	.	.	.	.	.	.	.	.	.	.	.	.	.	.	.	.	.	.	.	.	.	.	.	.	.	.	.	.	.	.	.	.	.	.	.	.	.	.	.	.	.	.	.	.	.	.	.	.	.	.	.	.	.	.
dQLQ4	.	.	.	.	.	.	.	.	.	.	.	.	.	.	.	.	.	.	.	.	.	.	.	.	.	.	.	.	.	.	.	.	.	.	.	.	.	.	.	.	.	.	.	.	.	.	.	.	.	.	.	.	.	.	.	.	.	.	.	.	.	.	.	.	.	.	.	.	.	.
dQLQ5	.	.	.	.	.	.	.	.	.	.	.	.	.	.	.	.	.	.	.	.	.	.	.	.	.	.	.	.	.	.	.	.	.	.	.	.	.	.	T	.	.	.	.	.	.	.	.	.	.	.	.	.	.	.	.	.	.	.	.	.	.	.	.	.	.	.	.	.	.	.

	71	72	73	74	75	76	77	78	79	80	81	82	83	84	85	86	87	88	89	90	91	92	93	94	95	96	97	98	99	100	101	102	103	104	105	106	107	108	109	110	111	112	113	114	115	116	117	118	119	120	121	122	123	124	125	126	127	128	129	130	131	132	133	134	135	136	137	138
dNZT1	A	A	A	T	A	A	T	T	C	C	A	G	T	C	A	A	C	A	T	G	C	G	T	A	T	C	A	C	C	A	C	C	A	T	T	A	G	A	T	C	A	C	G	A	G	C	T	T	A	A	T	T	A	C	C	A	T	G	C	C	G	C	G	T	G	A	A	A
dNZT2	.	.	.	.	.	.	.	.	.	.	.	.	.	.	.	.	.	.	.	.	.	.	.	.	.	.	.	.	.	.	.	.	.	.	.	.	.	.	.	.	.	.	.	.	.	.	.	.	.	.	.	.	.	.	.	.	.	.	.	.	.	.	.	.	.	.	.	.
dNZT3	.	.	.	.	.	.	.	.	.	.	.	.	.	.	.	.	.	.	.	.	.	.	.	.	.	.	.	.	.	.	.	.	.	.	.	.	.	.	.	.	.	.	.	.	.	.	.	.	.	.	.	.	.	.	.	.	.	.	.	.	.	.	.	.	.	.	.	.
dNZT4	.	.	.	.	.	.	C	.	.	.	.	.	.	.	.	.	.	.	.	.	.	.	.	.	.	.	.	.	.	.	.	.	.	.	.	.	.	.	.	.	.	.	.	.	.	.	.	.	.	.	.	.	.	.	.	.	.	.	.	.	.	.	.	.	.	.	.	.
dJH1	.	.	.	.	.	.	C	.	.	.	.	.	.	.	.	.	.	.	.	.	.	.	.	.	.	.	.	.	.	.	.	.	.	.	.	.	.	.	.	.	.	.	.	.	.	.	.	.	.	.	.	.	.	.	.	.	.	.	.	.	.	.	.	.	.	.	.	.
dJH2	.	.	.	.	.	.	.	.	.	.	.	.	.	.	.	.	.	.	.	.	.	.	.	.	.	.	.	.	.	.	.	.	.	.	.	.	.	.	.	.	.	.	.	.	.	.	.	.	.	.	.	.	.	.	.	.	.	.	.	.	.	.	.	.	.	.	.	.
dCS1	.	.	.	.	.	.	C	.	.	.	.	.	.	.	.	.	.	.	.	.	.	.	.	.	.	.	.	.	T	.	.	.	.	.	.	.	.	.	.	.	.	.	.	.	.	.	.	.	.	.	.	.	.	.	.	.	.	.	.	.	.	.	.	.	.	.	.	.
dCS2	.	.	.	.	.	.	.	.	.	.	.	.	.	.	.	.	.	.	.	.	.	.	.	.	.	.	.	.	.	.	.	.	.	.	.	.	.	.	.	.	.	.	.	.	.	.	.	.	.	.	.	.	.	.	.	.	.	.	.	.	.	.	.	.	.	.	.	.
dTS1	.	.	.	.	.	.	C	.	.	.	.	.	.	.	.	.	.	.	.	.	.	.	.	.	.	.	.	.	.	.	.	.	.	.	.	.	.	.	.	.	.	.	.	.	.	.	.	.	.	.	.	.	.	.	.	.	.	.	.	.	.	.	.	.	.	.	.	.
dTS2	.	.	.	.	.	.	C	.	.	.	.	.	.	.	.	.	.	.	.	.	.	.	.	.	.	.	.	.	.	.	.	.	.	.	.	.	.	.	.	.	.	.	.	.	.	.	.	.	.	.	.	.	.	.	.	.	.	.	.	.	.	.	.	.	.	.	.	.
dTS3	.	.	.	.	.	.	.	.	.	.	.	.	.	.	.	.	.	.	.	.	.	.	.	.	.	.	.	.	.	.	.	.	.	.	.	.	.	.	.	.	.	.	.	.	.	.	.	.	.	.	.	.	.	.	.	.	.	.	.	.	.	.	.	.	.	.	.	.
dTS4	.	.	.	.	.	.	C	.	.	.	.	.	.	.	.	.	.	.	.	.	.	.	.	.	.	.	.	.	.	.	.	.	.	.	.	.	.	.	.	.	.	.	.	.	.	.	.	.	.	.	.	.	.	.	.	.	.	.	.	.	.	.	.	.	.	.	.	.
dWD1	.	.	.	.	.	.	C	.	.	.	.	.	.	.	.	.	.	.	.	.	.	.	.	.	.	.	.	.	.	.	.	.	.	.	.	.	.	.	.	.	.	.	.	.	.	.	.	.	.	.	.	.	.	.	.	.	.	.	.	.	.	.	.	.	.	.	.	.
dWD2	.	.	.	.	.	.	C	.	.	.	.	.	.	.	.	.	.	.	.	.	.	.	.	.	.	.	.	.	T	.	.	.	.	.	.	.	.	.	.	.	.	.	.	.	.	.	.	.	.	.	.	.	.	.	.	.	.	.	.	.	.	.	.	.	.	.	.	.
dWD3	.	.	.	.	.	.	C	.	.	.	.	.	.	.	.	.	.	.	.	.	.	.	.	.	.	.	.	.	T	.	.	.	.	.	.	.	.	.	.	.	.	.	.	.	.	.	.	.	.	.	.	.	.	.	.	.	.	.	.	.	.	.	.	.	.	.	.	.
dGCJ1	.	.	.	.	.	.	.	.	.	.	.	.	.	.	.	.	.	.	.	.	.	.	.	.	.	.	.	.	.	.	.	.	.	.	.	.	.	.	.	.	.	.	.	.	.	.	C	.	.	.	.	.	.	.	.	.	.	.	.	.	.	.	.	.	.	.	.	.
dGCJ2	.	.	.	.	.	.	.	.	.	.	.	.	.	.	G	.	.	.	.	.	.	.	.	.	.	.	.	.	.	.	.	.	.	.	.	.	.	.	.	.	.	.	.	.	.	.	.	.	.	.	.	.	.	.	.	.	.	.	.	.	.	.	.	.	.	.	.	.
dGH1	.	.	.	.	.	.	C	.	.	.	.	.	.	.	.	.	.	.	.	.	.	.	.	.	.	.	.	.	.	.	.	.	.	.	.	.	.	.	.	.	.	.	.	.	.	.	.	.	.	.	.	.	.	.	.	.	.	.	.	.	.	.	.	.	.	.	.	.
dGH2	.	.	.	.	.	.	C	.	.	.	.	.	.	.	.	.	.	.	.	.	.	.	.	.	.	.	.	.	.	.	.	.	.	.	.	.	.	.	.	.	.	.	.	.	.	.	.	.	.	.	.	.	.	.	.	.	.	.	.	.	.	.	.	.	.	.	.	.
dGH3	.	.	.	.	.	.	C	.	.	.	.	.	.	.	.	.	.	.	.	.	.	.	.	.	.	.	.	.	.	.	.	.	.	.	.	.	.	.	.	.	.	.	.	.	.	.	.	.	.	.	.	.	.	.	.	.	.	.	.	.	.	.	.	.	.	.	.	.
dGH4	.	.	.	.	.	.	C	.	.	.	.	.	.	.	.	.	.	.	.	.	.	.	.	.	.	.	.	.	.	.	.	.	.	.	.	.	.	.	.	.	.	.	.	.	.	.	.	.	.	.	.	.	.	.	.	.	.	.	.	.	.	.	.	.	.	.	.	.
dGH5	.	.	.	.	.	.	C	.	.	.	.	.	.	.	.	.	.	.	.	.	.	.	.	.	.	.	.	.	.	.	.	.	.	.	.	.	.	.	.	.	.	.	.	.	.	.	.	.	.	.	.	.	.	.	.	.	.	.	.	.	.	.	.	.	.	.	.	.
dGH6	.	.	.	.	.	.	C	.	.	.	.	.	.	.	.	.	.	.	.	.	.	.	.	.	.	.	.	.	.	.	.	.	.	.	.	.	.	.	.	.	.	.	.	.	.	.	.	.	.	.	.	.	.	.	.	.	.	.	.	.	.	.	.	.	.	.	.	.
dWCG1	.	.	.	.	.	.	.	.	.	.	.	.	.	.	.	.	.	.	.	.	.	.	.	.	.	.	.	.	.	.	.	.	.	.	.	.	.	.	.	.	.	.	.	.	.	.	.	.	.	.	.	.	.	.	.	.	.	.	.	.	.	.	.	.	.	.	.	.
dXLW1	.	.	.	.	.	.	.	.	.	.	.	.	.	.	.	.	.	.	.	.	.	.	.	.	.	.	.	.	.	.	.	.	.	.	.	.	.	.	.	.	.	.	.	.	.	.	.	.	.	.	.	.	.	.	.	.	.	.	.	.	.	.	.	.	.	.	.	.
dXLW2	.	.	.	.	.	.	C	.	.	.	.	.	.	.	.	.	.	.	.	.	.	.	.	.	.	.	.	.	T	.	.	.	.	.	.	.	.	.	.	.	.	.	.	.	.	.	.	.	.	.	.	.	.	.	.	.	.	.	.	.	.	.	.	.	.	.	.	.
dWBP1	.	.	.	.	.	.	C	.	.	T	.	.	.	.	.	.	.	.	.	.	.	.	.	.	.	.	.	.	.	.	.	.	.	.	.	.	.	.	.	.	.	.	.	.	.	.	.	.	.	.	C	.	.	.	.	.	.	.	.	.	.	.	.	.	.	.	.	.
dDSQ1	.	.	.	.	.	.	C	.	.	.	.	.	.	.	.	.	.	.	.	.	.	.	.	.	.	.	.	.	.	.	.	.	.	.	.	.	.	.	.	.	.	.	.	.	.	.	.	.	.	.	.	.	.	.	.	.	.	.	.	.	.	.	.	.	.	.	.	.
dDSQ2	.	.	.	.	.	.	.	.	.	.	.	.	C	.	.	.	.	.	.	.	.	.	.	.	.	.	.	.	.	.	.	.	.	.	.	.	.	.	.	.	.	.	.	.	.	.	.	.	.	.	.	.	.	.	.	.	.	.	.	.	.	.	.	.	.	.	.	.
dCN1	.	.	.	.	.	.	.	.	.	.	.	.	.	.	.	.	.	.	.	.	.	.	.	.	.	.	.	.	.	.	.	.	.	.	.	.	.	.	.	.	.	.	.	.	.	.	.	.	.	.	.	.	.	.	.	.	.	.	.	.	.	.	.	.	.	.	.	.
dLJ1	.	.	.	.	.	.	C	.	.	.	.	.	.	.	.	.	.	.	.	.	.	.	.	.	.	.	.	.	.	.	.	.	.	.	.	.	.	.	.	.	.	.	.	.	.	.	.	.	.	.	.	.	.	.	.	.	.	.	.	.	.	.	.	.	.	.	.	.
dLJ2	.	.	.	.	.	.	C	.	.	.	.	.	.	.	.	.	.	.	.	.	.	.	.	.	.	.	.	.	.	.	.	.	.	.	.	.	.	.	.	.	.	.	.	.	.	.	.	.	.	.	.	.	.	.	.	.	.	.	.	.	.	.	.	.	.	.	.	.
dLJ3	.	.	.	.	.	.	C	.	.	.	.	.	.	.	.	.	.	.	.	.	.	.	.	.	.	.	.	.	.	.	.	.	.	.	.	.	.	.	.	.	.	.	.	.	.	.	.	.	.	.	.	.	.	.	.	.	.	.	.	.	.	.	.	.	.	.	.	.
dQLQ1	.	.	.	.	.	.	.	.	.	.	.	.	.	.	.	.	.	.	.	.	.	.	.	.	.	.	.	.	.	.	.	.	.	.	.	.	.	.	.	.	.	.	.	.	.	.	.	.	.	.	.	.	.	.	.	.	.	.	.	.	.	.	.	.	.	.	.	.
dQLQ2	.	.	.	.	.	.	.	.	.	.	.	.	.	.	.	.	.	.	.	.	.	.	.	.	.	.	.	.	.	.	.	.	.	.	.	.	.	.	.	.	.	.	.	.	.	.	.	.	.	.	.	.	.	.	.	.	.	.	.	.	.	.	.	.	.	.	.	.
dQLQ3	.	.	.	.	.	.	.	.	.	.	.	.	.	.	.	.	.	.	.	.	.	.	.	.	.	.	.	.	.	.	.	.	.	.	.	.	.	.	.	.	.	.	.	.	.	.	.	.	.	.	.	.	.	.	.	.	.	.	.	.	.	.	.	.	.	.	.	.
dQLQ4	.	.	.	.	.	.	.	.	.	.	.	.	.	.	.	.	.	.	.	.	.	.	.	.	.	.	.	.	.	.	.	.	.	.	.	.	.	.	.	.	.	.	.	.	.	.	.	.	.	.	.	.	.	.	.	.	.	.	.	.	.	.	.	.	.	.	.	.
dQLQ5	.	.	.	.	.	.	C	.	.	.	.	.	.	.	.	.	.	.	.	.	.	.	.	.	.	.	.	.	.	.	.	.	.	.	.	.	.	.	.	.	.	.	.	.	.	.	.	.	.	.	.	.	.	.	.	.	.	.	.	.	.	.	.	.	.	.	.	.

Note: The CR sequences with green background present the ones published in our previous studies (Larson et al., 2010 and Wang et al., 2012).

Table S5. Radiocarbon Dating Results. AMS dating on bone collagen was performed by Beta Analytic Inc.

Site	Sample ID	Beta No.	Percent Modern Carbon (pMC)	Fraction Modern	D14C	Measured Radiocarbon Age	13C/12C Ratio	Conventional Radiocarbon Age	2 Sigma Calibrated Dates
Cishan	S3	Beta-389044	43.1 +/- 0.2 pMC	0.4310 +/- 0.0016	-569.0 +/- 1.6 o/oo	6710 +/- 30 BP	-21.9 o/oo	6760 +/- 30 BP	Cal BC 5715 to 5625 (Cal BP 7665 to 7575)
	S7	Beta-389045	42.5 +/- 0.2 pMC	0.4247 +/- 0.0016	-575.3 +/- 1.6 o/oo	6820 +/- 30 BP	-21.2 o/oo	6880 +/- 30 BP	Cal BC 5835 to 5825 (Cal BP 7785 to 7775) and Cal BC 5810 to 5715 (Cal BP 7760 to 7665)
Nanzhuangtou	NT6	Beta-389048	31.7 +/- 0.1 pMC	0.3169 +/- 0.0012	-683.1 +/- 1.2 o/oo	9200 +/- 30 BP	-23.0 o/oo	9230 +/- 30 BP	Cal BC 8555 to 8320 (Cal BP 10505 to 10270)
Xinglongwa	X3	Beta-389042	43.7 +/- 0.2 pMC	0.4370 +/- 0.0016	-563.0 +/- 1.6 o/oo	6580 +/- 30 BP	-20.7 o/oo	6650 +/- 30 BP	Cal BC 5630 to 5530 (Cal BP 7580 to 7480)

Table S6. The 37 combined ancient pig sequences and 97 extant published homologous sequences.

Haplotype label	Haplotype size	Sequence label	Breeed/Status	Region
1	23	AF304200.1	Meishan pig	China
		AF486859.1	Xiang	China
		AF486860.1	Rongchang	China
		AY574048.1	Large White	
		EF545567.1	Saba pig	China:Yunnan
		EF545573.1	wild boar	China:Yunnan
		EF545575.1	Shanggao Mengshan	China:Jiangxi
		EF545576.1	Zang	China:Yunnan
		EF545579.1	wild boar	China:Jiangxi
		EF545587.1	Huzhu pig	China
		EF545589.1	Yimeng Black	China:Shandong
		KC250274.1	Lantang	China:Guangdong
		DSQ1	ancient pig	China
		GH1	ancient pig	China
		GH3	ancient pig	China
		GH5	ancient pig	China
		GH6	ancient pig	China
		LJ1	ancient pig	China
		LJ2	ancient pig	China
		LJ3	ancient pig	China
		TS1	ancient pig	China
		TS2	ancient pig	China
		TS4	ancient pig	China
2	8	AF304202.1	Landrace	
		AF486857.1	Ningxiang	China
		AF486861.1	Erhualian	China
		AF486870.1	Dahua White	China
		EF545570.1	wild boar	China:Fujian
		KC250273.1	Xiang	China:Guangdong
		WD2	ancient pig	China
		XLW2	ancient pig	China
3	12	AF034253.1	Landrace	
		AF304203.1	Swedish wild boar	Sweden
		AF486866.1	Landrace	
		AY337045.1	Duroc	
		AY574046.1	Hampshire	
		FJ236997.1	Duroc	
		FJ236999.1	European wild boar	Europe
		FJ237000.1	European wild boar	Europe
		FJ237001.1	European wild boar	Europe
		FJ237002.1	European wild boar	Europe
		FJ237003.1	European wild boar	Europe
		NC_000845.1	Landrace	
4	1	EF545568.1	wild boar	China:Yunnan
5	13	AB298688.1	Ohmini Pig	Japan
		AF486856.1	Zang	China
		AF486867.1	Wuzhishan	China:Hainan
		DQ466081.2	Nuogu pig	China:Guizhou
		DQ972936.3	Lanyu pig	Taiwan
		EF545569.1	wild boar	China:Fujian
		EF545584.1	wild boar	Vietnam

		EF545588.1	Huzhu pig	China
		GQ220329.1	Dahe	China:Yunnan
		GH2	ancient pig	China
		GH4	ancient pig	China
		JH1	ancient pig	China
		NZT3	ancient pig	China
6	1	EF545571.1	wild boar	China:Fujian
7	11	EF545572.1	wild boar	China:Hainan
		EF545577.1	Wei pig	China:Anhui
		EF545578.1	Aba pig	China:Sichuan
		EF545581.1	Qingping	China:Fujian
		EF545582.1	Qingping	China:Fujian
		EF545590.1	Bihu	China
		CN1	ancient pig	China
		CS2	ancient pig	China
		JH2	ancient pig	China
		QLQ1	ancient pig	China
		XLW1	ancient pig	China
8	1	EF545574.1	Saba pig	China:Yunnan
9	1	EF545580.1	wild boar	China
10	19	AF486855.1	Zhong Meishan	China
		AF486862.1	Tongcheng	China
		AF486863.1	Jinhua	China
		AF486864.1	Min	China
		AF486865.1	Qingping	China
		AF486868.1	Yimeng Black	China
		AF486871.1	Yushan Black	China
		AF486872.1	Jiangquhai	China
		AF486873.1	Wannan Spotted	China
		AF486874.1	Large White	
		AY334492.2	Jeju native black	South Korea
		DQ334860.1	Jeju native	South Korea
		EF545583.1	Bamei	China
		EF545591.1	Bihu	China
		NZT1	ancient pig	China
		NZT2	ancient pig	China
		QLQ4	ancient pig	China
		TS3	ancient pig	China
		WCG1	ancient pig	China
11	1	EF545585.1	wild boar	China:Yunnan Ruili
12	1	EF545586.1	wild boar	China:Yunnan
13	1	EF545592.1	wild boar	Malaysia
14	1	EF545593.1	Xiang pig	China
15	1	EU117375.1	Iberian	Spain
16	1	EU090702.1	Korean wild boar	South Korea
17	1	EU090703.1	Korean wild boar	South Korea
18	2	DQ518915.2	Lanyu pig	Taiwan
		EF375877.3	Lanyu pig	Taiwan
19	1	EU333163.1	Chinese northeast wildboar	China
20	1	FJ236991.1	Iberian	Spain
21	3	FJ236992.1	Iberian	Spain
		FJ236993.1	Iberian	Spain
		FJ236995.1	Iberian	Spain
22	1	FJ236994.1	Iberian	Spain

23	3	FJ236996.1	Duroc	
		FJ236998.1	European wild boar	Europe
		KC250275.1	Large White	
24	3	AP003428.1	Large White	
		NC_012095.1	Large White	
		AF486858.1	Duroc	
25	2	AF486869.1	Diannan Short-ear	China
		GQ220328.1	Banna Mini	China:Yunnan
26	2	GU147934.1	Taiwanesis pig	Taiwan
		NC_014692.1	taiwanensis	Taiwan
27	1	DQ534707.2	Taoyuan pig	Taiwan
28	1	AY574045.1	Berkshire	
29	2	AY574047.1	Korean Wild Boar	South Korea
		DQ207755.1	Korean wild boar	South Korea
30	1	DQ207754.1	Korean wild boar	South Korea
31	1	DQ207753.1	Korean wild boar	South Korea
32	1	DQ268530.1	Korean wild boar	South Korea
33	1	DQ274110.1	Jeju native	South Korea
34	1	DQ334861.1	Jeju native	South Korea
35	2	CS1	ancient pig	China
		WD3	ancient pig	China
36	1	DSQ2	ancient pig	China
37	1	GCJ1	ancient pig	China
38	1	GCJ2	ancient pig	China
39	1	QLQ2	ancient pig	China
40	1	QLQ3	ancient pig	China
41	1	QLQ5	ancient pig	China
42	1	WBP1	ancient pig	China
43	1	WD1	ancient pig	China

Table S7. The mtDNA *Cytb* gene sequences used in this analysis.

Haplotype label	Haplotype size	Sequence label	Breeed/Status	Region
1	502	AB015065.1	Sus scrofa Japanese wild boar	Japan
		AB015066.1	Sus scrofa Japanese wild boar	Japan
		AB015067.1	Sus scrofa Japanese wild boar	Japan
		AB015068.1	Sus scrofa Japanese wild boar	Japan
		AB015069.1	Sus scrofa Japanese wild boar	Japan
		AB015070.1	Sus scrofa Japanese wild boar	Japan
		AB015071.1	Sus scrofa Ryukyu wild boar	Japan:Ryukyu
		AB015072.1	Sus scrofa Ryukyu wild boar	Japan:Ryukyu
		AB015073.1	Sus scrofa Ryukyu wild boar	Japan:Ryukyu
		AB015076.1	Sus scrofa domesticus	Japan
		AB015077.1	Sus scrofa Meishan pig	China
		AB298688.1	Sus scrofa Ohmini Pig	Japan
		AF304200.1	Sus scrofa Meishan pig	China
		AF486855.1	Sus scrofa Zhong Meishan	China
		AF486856.1	Sus scrofa Zang	China
		AF486857.1	Sus scrofa Ningxiang	China
		AF486859.1	Sus scrofa Xiang	China
		AF486860.1	Sus scrofa Rongchang	China
		AF486861.1	Sus scrofa Erhualian	China
		AF486862.1	Sus scrofa Tongcheng	China
		AF486863.1	Sus scrofa Jinhua	China
		AF486864.1	Sus scrofa Min	China
		AF486865.1	Sus scrofa Qingping	China
		AF486868.1	Sus scrofa Yimeng Black	China
		AF486869.1	Sus scrofa Diannan Short-ear	China
		AF486870.1	Sus scrofa Dahua White	China
		AF486871.1	Sus scrofa Yushan Black	China
		AF486872.1	Sus scrofa Jiangquhai	China
		AF486873.1	Sus scrofa Wannan Spotted	China
		AJ314557.1	Sus scrofa domesticus	Japan
		AM492546.1	Sus scrofa	Indonesia:Asmat Territories-West New Guinea
		AM492547.1	Sus scrofa	Indonesia:Asmat Territories-West New Guinea
		AM492548.1	Sus scrofa	Indonesia:Asmat Territories-West New Guinea
		AM492549.1	Sus scrofa	Indonesia:Asmat Territories-West New Guinea
		AM492550.1	Sus scrofa	Indonesia:Asmat Territories-West New Guinea
		AM492551.1	Sus scrofa	Indonesia:Asmat Territories-West New Guinea
		AM492552.1	Sus scrofa	Indonesia:Asmat Territories-West New Guinea
		AM492553.1	Sus scrofa	Indonesia:Asmat Territories-West New Guinea
		AM492554.1	Sus scrofa	Indonesia:Asmat Territories-West New Guinea
		AM492555.1	Sus scrofa	Indonesia:Asmat Territories-West New Guinea
		AM492556.1	Sus scrofa	Indonesia:Asmat Territories-West New Guinea
		AM492557.1	Sus scrofa	Indonesia:Asmat Territories-West New Guinea
		AM492561.1	Sus scrofa	Indonesia:Bali
		AM492562.1	Sus scrofa	Indonesia:Bali
		AM492563.1	Sus scrofa	Indonesia:Bali
		AM492564.1	Sus scrofa	Indonesia:Bali
		AM492565.1	Sus scrofa	Indonesia:Bali
		AM492566.2	Sus scrofa	Indonesia:Bali
		AM492567.1	Sus scrofa	Indonesia:Bali
		AM492568.1	Sus scrofa	Indonesia:Bali
		AM492569.1	Sus scrofa	Indonesia:Bali

		AM492570.1	Sus scrofa	Indonesia:Bali
		AM492571.1	Sus scrofa	Indonesia:Manokwari-West New Guinea
		AM492572.1	Sus scrofa	Indonesia:Manokwari-West New Guinea
		AM492575.1	Sus scrofa	Indonesia:Nabire-West New Guinea
		AM492576.1	Sus scrofa	Indonesia:Nabire-West New Guinea
		AM492577.1	Sus scrofa	Indonesia:Nabire-West New Guinea
		AM492578.1	Sus scrofa	Indonesia:Nabire-West New Guinea
		AM492579.1	Sus scrofa	Indonesia:Nabire-West New Guinea
		AM492580.1	Sus scrofa	Indonesia:Nabire-West New Guinea
		AM492581.1	Sus scrofa	Indonesia:Nabire-West New Guinea
		AM492582.1	Sus scrofa	Indonesia:Nabire-West New Guinea
		AM492583.1	Sus scrofa	Indonesia:Nabire-West New Guinea
		AM492584.1	Sus scrofa	Indonesia:Nabire-West New Guinea
		AM492585.1	Sus scrofa	Indonesia:Nabire-West New Guinea
		AM492586.1	Sus scrofa	Indonesia:Nabire-West New Guinea
		AM492587.1	Sus scrofa	Indonesia:Nabire-West New Guinea
		AM492588.1	Sus scrofa	Indonesia:Nabire-West New Guinea
		AM492589.1	Sus scrofa	Indonesia:Nabire-West New Guinea
		AM492590.1	Sus scrofa	Indonesia:Nabire-West New Guinea
		AM492591.1	Sus scrofa	Indonesia:Kecamantan Simpang Hulu-Borneo
		AM492594.1	Sus scrofa	Sri Lanka
		AM492595.1	Sus scrofa	Sri Lanka
		AM492596.1	Sus scrofa	Sri Lanka
		AM492597.1	Sus scrofa	China:Sichuan Zizhong
		AM492598.1	Sus scrofa	China:Sichuan Zizhong
		AM492599.1	Sus scrofa	China:Sichuan Zizhong
		AM492600.1	Sus scrofa	China:Sichuan Ziyang
		AM492601.1	Sus scrofa	China:Sichuan Jingtang
		AM492602.1	Sus scrofa	China:Sichuan Jingtang
		AM492603.1	Sus scrofa	China:Sichuan Wenjiang
		AM492604.1	Sus scrofa	China:Sichuan Shangliu
		AM492605.2	Sus scrofa	China:Sichuan Shangliu
		AM492606.1	Sus scrofa	China:Sichuan Huayang
		AM492607.2	Sus scrofa	China:Sichuan Wuhou District
		AM492608.1	Sus scrofa	China:Sichuan Jingtang
		AM492609.1	Sus scrofa	China:Sichuan Xindu
		AM492610.1	Sus scrofa	Indonesia:Mukuwashi-Flores
		AM492613.1	Sus scrofa	Indonesia:Nabire Highlands-West New Guinea
		AM492614.1	Sus scrofa	Indonesia:Nabire Highlands-West New Guinea
		AM492615.1	Sus scrofa	Indonesia:Nabire Highlands-West New Guinea
		AM492616.1	Sus scrofa	Indonesia:Nabire Highlands-West New Guinea
		AM492627.1	Sus scrofa	Indonesia:Solo-Java
		AM492628.2	Sus scrofa	Indonesia:Solo-Java
		AM492629.1	Sus scrofa	Indonesia:Solo-Java
		AM492630.1	Sus scrofa	Indonesia:Solo-Java
		AM492631.1	Sus scrofa	Indonesia:Solo-Java
		AM492632.1	Sus scrofa	Indonesia:Solo-Java
		AM492633.1	Sus scrofa	Indonesia:Solo-Java
		AM492634.1	Sus scrofa	Indonesia:Bandung-Java
		AM492635.1	Sus scrofa	Indonesia:Bandung-Java
		AM492636.1	Sus scrofa	Indonesia:Bandung-Java
		AM492638.1	Sus scrofa	Indonesia:Bandung-Java
		AM492639.1	Sus scrofa	Indonesia:Bandung-Java
		AM492641.1	Sus scrofa	Indonesia:Bandung-Java

		AM492642.1	Sus scrofa	Indonesia:Bandung-Java
		AM492643.1	Sus scrofa	Indonesia:Bandung-Java
		AM492644.1	Sus scrofa	Indonesia:Bandung-Java
		AM492655.1	Sus scrofa	Vietnam
		AM492656.1	Sus scrofa	Vietnam
		AM492657.1	Sus scrofa	Vietnam
		AY334492.2	Sus scrofa Jeju native black	South Korea
		AY574047.1	Sus scrofa Korean Wild Boar	South Korea
		AY634181.1	Sus scrofa Korean wild boar	South Korea
		AY634182.1	Sus scrofa Korean wild boar	South Korea
		AY634183.1	Sus scrofa Korean wild boar	South Korea
		AY634184.1	Sus scrofa Korean wild boar	South Korea
		AY634185.1	Sus scrofa Korean wild boar	South Korea
		AY634186.1	Sus scrofa Korean wild boar	South Korea
		AY634187.1	Sus scrofa Korean wild boar	South Korea
		AY692030.1	Sus scrofa Korean wild boar	South Korea
		AY692031.1	Sus scrofa Korean wild boar	South Korea
		AY830160.1	Sus scrofa coreanus	South Korea
		AY830163.1	Sus scrofa coreanus	South Korea
		AY830164.1	Sus scrofa coreanus	South Korea
		AY830165.1	Sus scrofa coreanus	South Korea
		AY830166.1	Sus scrofa coreanus	South Korea
		AY830167.1	Sus scrofa coreanus	South Korea
		AY830169.1	Sus scrofa coreanus	South Korea
		AY830170.1	Sus scrofa coreanus	South Korea
		AY830171.1	Sus scrofa coreanus	South Korea
		AY830172.1	Sus scrofa coreanus	South Korea
		AY830180.1	Sus scrofa Jeju native	South Korea
		AY830181.1	Sus scrofa Jeju native	South Korea
		AY830182.1	Sus scrofa Jeju native	South Korea
		AY830184.1	Sus scrofa Jeju native	South Korea
		DQ207754.1	Sus scrofa Korean wild boar	South Korea
		DQ207755.1	Sus scrofa Korean wild boar	South Korea
		DQ274110.1	Sus scrofa Jeju native	South Korea
		DQ315597.1	Sus scrofa Hainan wild boar	China:Hainan
		DQ315598.1	Sus scrofa Hainan wild boar	China:Hainan
		DQ315599.1	Sus scrofa Yunnan wild boar	China:Yunnan
		DQ315600.1	Sus scrofa Yunnan wild boar	China:Yunnan
		DQ315603.1	Sus scrofa Vietnam wild boar	Vietnam
		DQ315604.1	Sus scrofa Wei pig	China:Anhui
		DQ334860.1	Sus scrofa Jeju native	South Korea
		DQ334861.1	Sus scrofa Jeju native	South Korea
		DQ466081.2	Sus scrofa Nuogu pig	China:Guizhou
		DQ518915.2	Sus scrofa Lanyu pig	China:Taiwan
		DQ972936.3	Sus scrofa Lanyu pig	China:Taiwan
		EF375877.3	Sus scrofa Lanyu pig	China:Taiwan
		EF545567.1	Sus scrofa Saba pig	China:Yunnan
		EF545569.1	Sus scrofa wild boar	China:Fujian
		EF545570.1	Sus scrofa wild boar	China:Fujian
		EF545571.1	Sus scrofa wild boar	China:Fujian
		EF545572.1	Sus scrofa wild boar	China:Hainan
		EF545573.1	Sus scrofa wild boar	China:Yunnan
		EF545574.1	Sus scrofa Saba pig	China:Yunnan
		EF545575.1	Sus scrofa Shanggao Mengshan	China:Jiangxi

EF545576.1	Sus scrofa Zang	China:Yunnan
EF545577.1	Sus scrofa Wei pig	China:Anhui
EF545578.1	Sus scrofa Aba pig	China:Sichuan
EF545579.1	Sus scrofa wild boar	China:Jiangxi
EF545580.1	Sus scrofa wild boar	China
EF545581.1	Sus scrofa Qingping	China
EF545582.1	Sus scrofa Qingping	China
EF545583.1	Sus scrofa Bamei	China
EF545584.1	Sus scrofa wild boar	Vietnam
EF545585.1	Sus scrofa wild boar	China:Yunnan Ruili
EF545586.1	Sus scrofa wild boar	China:Yunnan
EF545587.1	Sus scrofa Huzhu pig	China
EF545588.1	Sus scrofa Huzhu pig	China
EF545589.1	Sus scrofa Yimeng Black	China:Shandong
EF545590.1	Sus scrofa Bihu	China
EF545591.1	Sus scrofa Bihu	China
EF545593.1	Sus scrofa Xiang pig	China
EU000390.1	Sus scrofa taiwanensis	China:Taiwan
EU000391.1	Sus scrofa taiwanensis	China:Taiwan
EU000392.1	Sus scrofa taiwanensis	China:Taiwan
EU090702.1	Sus scrofa Korean wild boar	South Korea
EU333163.1	Sus scrofa Chinese northeast wildboar	China
EU660056.1	Sus scrofa Yantai black	China:Shandong
EU660057.1	Sus scrofa Yantai black	China:Shandong
EU660058.1	Sus scrofa Yantai black	China:Shandong
EU660059.1	Sus scrofa Yantai black	China:Shandong
EU660060.1	Sus scrofa Yantai black	China:Shandong
EU660061.1	Sus scrofa Yantai black	China:Shandong
EU660062.1	Sus scrofa Yantai black	China:Shandong
EU660063.1	Sus scrofa Yantai black	China:Shandong
EU660064.1	Sus scrofa Yantai black	China:Shandong
EU660065.1	Sus scrofa Yantai black	China:Shandong
EU660084.1	Sus scrofa Laiwu black	China:Shandong
EU660085.1	Sus scrofa Laiwu black	China:Shandong
EU660086.1	Sus scrofa Laiwu black	China:Shandong
EU660087.1	Sus scrofa Laiwu black	China:Shandong
EU660088.1	Sus scrofa Laiwu black	China:Shandong
EU660089.1	Sus scrofa Laiwu black	China:Shandong
EU660090.1	Sus scrofa Laiwu black	China:Shandong
EU660091.1	Sus scrofa Licha black	China:Shandong
EU660092.1	Sus scrofa Licha black	China:Shandong
EU660093.1	Sus scrofa Licha black	China:Shandong
EU660094.1	Sus scrofa Licha black	China:Shandong
EU660095.1	Sus scrofa Licha black	China:Shandong
EU660096.1	Sus scrofa Licha black	China:Shandong
EU660097.1	Sus scrofa Licha black	China:Shandong
EU660098.1	Sus scrofa Licha black	China:Shandong
EU660099.1	Sus scrofa Dapulian black	China:Shandong
EU660100.1	Sus scrofa Dapulian black	China:Shandong
EU660101.1	Sus scrofa Dapulian black	China:Shandong
EU660102.1	Sus scrofa Dapulian black	China:Shandong
EU660103.1	Sus scrofa Dapulian black	China:Shandong
EU660104.1	Sus scrofa Dapulian black	China:Shandong
EU660105.1	Sus scrofa Dapulian black	China:Shandong

EU660106.1	Sus scrofa Dapulian black	China:Shandong
EU660107.1	Sus scrofa Dapulian black	China:Shandong
EU660124.1	Sus scrofa Yimeng black	China:Shandong
EU660125.1	Sus scrofa Yimeng black	China:Shandong
EU660126.1	Sus scrofa Yimeng black	China:Shandong
EU660127.1	Sus scrofa Yimeng black	China:Shandong
EU660128.1	Sus scrofa Yimeng black	China:Shandong
EU660129.1	Sus scrofa Yimeng black	China:Shandong
EU660130.1	Sus scrofa Yimeng black	China:Shandong
EU979222.1	Sus scrofa Yimeng black	China:Shandong
EU979223.1	Sus scrofa Yimeng black	China:Shandong
EU979224.1	Sus scrofa Yimeng black	China:Shandong
EU979225.1	Sus scrofa Yimeng black	China:Shandong
EU979226.1	Sus scrofa Yimeng black	China:Shandong
EU979227.1	Sus scrofa Yimeng black	China:Shandong
EU979228.1	Sus scrofa Yimeng black	China:Shandong
EU979229.1	Sus scrofa Yimeng black	China:Shandong
EU979230.1	Sus scrofa Wulian Black	China:Shandong
EU979231.1	Sus scrofa Wulian Black	China:Shandong
EU979232.1	Sus scrofa Wulian Black	China:Shandong
EU979233.1	Sus scrofa Wulian Black	China:Shandong
EU979234.1	Sus scrofa Wulian Black	China:Shandong
EU979235.1	Sus scrofa Wulian Black	China:Shandong
EU979236.1	Sus scrofa Wulian Black	China:Shandong
EU979237.1	Sus scrofa Wulian Black	China:Shandong
EU979238.1	Sus scrofa Wulian Black	China:Shandong
EU979239.1	Sus scrofa Wulian Black	China:Shandong
EU979240.1	Sus scrofa Wulian Black	China:Shandong
EU979241.1	Sus scrofa Wulian Black	China:Shandong
EU979242.1	Sus scrofa Wulian Black	China:Shandong
EU979243.1	Sus scrofa Wulian Black	China:Shandong
EU979244.1	Sus scrofa Wulian Black	China:Shandong
EU979245.1	Sus scrofa Wulian Black	China:Shandong
EU979246.1	Sus scrofa Changwei White	China:Shandong
EU979247.1	Sus scrofa Changwei White	China:Shandong
EU979248.1	Sus scrofa Changwei White	China:Shandong
EU979249.1	Sus scrofa Changwei White	China:Shandong
EU979250.1	Sus scrofa Changwei White	China:Shandong
EU979251.1	Sus scrofa Changwei White	China:Shandong
EU979252.1	Sus scrofa Changwei White	China:Shandong
EU979253.1	Sus scrofa Changwei White	China:Shandong
EU979254.1	Sus scrofa Changwei White	China:Shandong
EU979255.1	Sus scrofa Changwei White	China:Shandong
EU979256.1	Sus scrofa Changwei White	China:Shandong
EU979257.1	Sus scrofa Changwei White	China:Shandong
EU979258.1	Sus scrofa Changwei White	China:Shandong
EU979259.1	Sus scrofa Changwei White	China:Shandong
EU979260.1	Sus scrofa Yantai black	China:Shandong
EU979261.1	Sus scrofa Yantai black	China:Shandong
EU979262.1	Sus scrofa Yantai black	China:Shandong
EU979263.1	Sus scrofa Yantai black	China:Shandong
EU979264.1	Sus scrofa Yantai black	China:Shandong
EU979265.1	Sus scrofa Laiwu black	China:Shandong
EU979266.1	Sus scrofa Laiwu black	China:Shandong

EU979267.1	Sus scrofa Laiwu black	China:Shandong
EU979268.1	Sus scrofa Laiwu black	China:Shandong
EU979269.1	Sus scrofa Laiwu black	China:Shandong
EU979270.1	Sus scrofa Laiwu black	China:Shandong
EU979271.1	Sus scrofa Laiwu black	China:Shandong
EU979272.1	Sus scrofa Laiwu black	China:Shandong
EU979273.1	Sus scrofa Dapulian black	China:Shandong
EU979274.1	Sus scrofa Dapulian black	China:Shandong
EU979275.1	Sus scrofa Dapulian black	China:Shandong
EU979276.1	Sus scrofa Dapulian black	China:Shandong
EU979277.1	Sus scrofa Dapulian black	China:Shandong
EU979278.1	Sus scrofa Dapulian black	China:Shandong
EU979279.1	Sus scrofa Licha black	China:Shandong
EU979280.1	Sus scrofa Licha black	China:Shandong
EU979281.1	Sus scrofa Licha black	China:Shandong
EU979282.1	Sus scrofa Licha black	China:Shandong
EU979283.1	Sus scrofa Licha black	China:Shandong
EU979284.1	Sus scrofa Licha black	China:Shandong
EU979285.1	Sus scrofa Licha black	China:Shandong
GQ220328.1	Sus scrofa Banna Mini	China:Yunnan
GQ220329.1	Sus scrofa Dahe	China:Yunnan
GU135654.1	Sus scrofa Jianli	China:Hubei
GU135655.1	Sus scrofa Dongchuan	China:Jiangsu
GU135656.1	Sus scrofa Dongchuan	China:Jiangsu
GU135657.1	Sus scrofa Jinhua	China:Zhejiang
GU135658.1	Sus scrofa Jinhua	China:Zhejiang
GU135659.1	Sus scrofa Jiaxing black	China:Zhejiang
GU135660.1	Sus scrofa Jiaxing black	China:Zhejiang
GU135662.1	Sus scrofa Jiangquhai	China:Jiangsu
GU135663.1	Sus scrofa Shawutou	China:Jiangsu
GU135664.1	Sus scrofa Shawutou	China:Jiangsu
GU135665.1	Sus scrofa Banna Mini	China:Yunnan
GU135666.1	Sus scrofa Banna Mini	China:Yunnan
GU135667.1	Sus scrofa Hetao big ear	China:Inner Mongolia
GU135668.1	Sus scrofa Hetao big ear	China:Inner Mongolia
GU135669.1	Sus scrofa Yanan	China:Sichuan
GU135670.1	Sus scrofa Yanan	China:Sichuan
GU135671.1	Sus scrofa Neijiang	China:Sichuan
GU135672.1	Sus scrofa Neijiang	China:Sichuan
GU135673.1	Sus scrofa Xingzi black	China:Jiangxi
GU135674.1	Sus scrofa Xingzi black	China:Jiangxi
GU135675.1	Sus scrofa Nancheng	China:Jiangxi
GU135676.1	Sus scrofa Nancheng	China:Jiangxi
GU135677.1	Sus scrofa Dahe	China:Yunnan
GU135678.1	Sus scrofa Dahe	China:Yunnan
GU135679.1	Sus scrofa Leping spotted	China:Jiangxi
GU135680.1	Sus scrofa Leping spotted	China:Jiangxi
GU135681.1	Sus scrofa Small Meishan	China:Jiangsu
GU135682.1	Sus scrofa Small Meishan	China:Jiangsu
GU135683.1	Sus scrofa Banna small ear	China:Yunnan
GU135684.1	Sus scrofa Banna small ear	China:Yunnan
GU135685.1	Sus scrofa Saba	China:Yunnan
GU135686.1	Sus scrofa Saba	China:Yunnan
GU135687.1	Sus scrofa Baoshan	China:Yunnan

GU135688.1	Sus scrofa Baoshan	China:Yunnan
GU135689.1	Sus scrofa Dongxiang spotted	China:Jiangxi
GU135690.1	Sus scrofa Dongxiang spotted	China:Jiangxi
GU135691.1	Sus scrofa Shandi	China:Hunan
GU135692.1	Sus scrofa Shandi	China:Hunan
GU135693.1	Sus scrofa Rongchang	China:Sichuan
GU135694.1	Sus scrofa Rongchang	China:Sichuan
GU135695.1	Sus scrofa Wenchang	China:Hainan
GU135696.1	Sus scrofa Wenchang	China:Hainan
GU135697.1	Sus scrofa Licha black	China:Shandong
GU135698.1	Sus scrofa Licha black	China:Shandong
GU135699.1	Sus scrofa Hanjiang black	China:Shaanxi Xi-An
GU135700.1	Sus scrofa Hanjiang black	China:Shaanxi Xi-An
GU135701.1	Sus scrofa Baixi	China:Guizhou
GU135702.1	Sus scrofa Baixi	China:Guizhou
GU135703.1	Sus scrofa Yimeng black	China:Shandong
GU135704.1	Sus scrofa Yimeng black	China:Shandong
GU135705.1	Sus scrofa Guanling	China:Guizhou
GU135706.1	Sus scrofa Guanling	China:Guizhou
GU135707.1	Sus scrofa Laiwu black	China:Shandong
GU135708.1	Sus scrofa Laiwu black	China:Shandong
GU135711.1	Sus scrofa Jiangkoluobo	China:Guizhou
GU135712.1	Sus scrofa Jiangkoluobo	China:Guizhou
GU135715.1	Sus scrofa Qianbei black	China:Guizhou
GU135716.1	Sus scrofa Qianbei black	China:Guizhou
GU135717.1	Sus scrofa Qingping	China:Hubei
GU135718.1	Sus scrofa Qingping	China:Hubei
GU135721.1	Sus scrofa Qiandong spotted	China:Guizhou
GU135722.1	Sus scrofa Qiandong spotted	China:Guizhou
GU135723.1	Sus scrofa Wannan spotted	China:Anhui
GU135724.1	Sus scrofa Wannan spotted	China:Anhui
GU135727.1	Sus scrofa Tongcheng	China:Hubei
GU135728.1	Sus scrofa Tongcheng	China:Hubei
GU135729.1	Sus scrofa Yangxin	China:Hubei
GU135730.1	Sus scrofa Yangxin	China:Hubei
GU135731.1	Sus scrofa Min	China:Heilongjiang
GU135732.1	Sus scrofa Min	China:Heilongjiang
GU135733.1	Sus scrofa Shanggao	China:Jiangxi
GU135734.1	Sus scrofa Shanggao	China:Jiangxi
GU135735.1	Sus scrofa Guangdong spotted	China:Guangdong
GU135736.1	Sus scrofa Guangdong spotted	China:Guangdong
GU135737.1	Sus scrofa Yushan	China:Jiangxi
GU135738.1	Sus scrofa Yushan	China:Jiangxi
GU135739.1	Sus scrofa Qinghai Bamei	China:Qinghai
GU135740.1	Sus scrofa Qinghai Bamei	China:Qinghai
GU135741.1	Sus scrofa Shaziling	China:Hunan
GU135742.1	Sus scrofa Putian	China:Fujian
GU135743.1	Sus scrofa Putian	China:Fujian
GU135744.1	Sus scrofa Wuyi black	China:Fujian
GU135745.1	Sus scrofa Wuyi black	China:Fujian
GU135746.1	Sus scrofa Huai	China:Fujian
GU135747.1	Sus scrofa Huai	China:Fujian
GU135749.1	Sus scrofa Erhualian	China:Jiangsu
GU135750.1	Sus scrofa Wuzhishan	China:Hainan

GU135751.1	Sus scrofa Wuzhishan	China:Hainan
GU135752.1	Sus scrofa Junan	China:Shandong
GU135753.1	Sus scrofa Junan	China:Shandong
GU135754.1	Sus scrofa Taoyuan	China:Taiwan
GU135755.1	Sus scrofa Taoyuan	China:Taiwan
GU135756.1	Sus scrofa Kele	China:Guizhou
GU135757.1	Sus scrofa Kele	China:Guizhou
GU135758.1	Sus scrofa Chenghua	China:Sichuan
GU135759.1	Sus scrofa Chenghua	China:Sichuan
GU135760.1	Sus scrofa Wan-An spotted	China:Jiangxi
GU135761.1	Sus scrofa Wan-An spotted	China:Jiangxi
GU135762.1	Sus scrofa Longlin	China:Guangxi
GU135763.1	Sus scrofa Longlin	China:Guangxi
GU135764.1	Sus scrofa Xiang	China:Guizhou
GU135765.1	Sus scrofa Xiang	China:Guizhou
GU135766.1	Sus scrofa Donglan	China:Guangxi
GU135767.1	Sus scrofa Donglan	China:Guangxi
GU135769.1	Sus scrofa Daweizi	China:Hunan
GU135771.1	Sus scrofa Luchuan	China:Guangxi
GU135773.1	Sus scrofa Dongshan	China:Guangxi
GU135774.1	Sus scrofa Shangyu spotted	China:Zhejiang
GU135775.1	Sus scrofa Shangyu spotted	China:Zhejiang
GU135777.1	Sus scrofa Guizhong	China:Guangxi
GU135778.1	Sus scrofa Ningxiang	China:Hunan
GU135779.1	Sus scrofa Ningxiang	China:Hunan
GU135780.1	Sus scrofa Exi black	China:Hubei
GU135781.1	Sus scrofa Exi black	China:Hubei
GU135782.1	Sus scrofa Bama	China:Guangxi
GU135785.1	Sus scrofa Zang	China:Tibet
GU135786.1	Sus scrofa Zhejiang wildboar	China:Zhejiang
GU135787.1	Sus scrofa Zhejiang wildboar	China:Zhejiang
GU135788.1	Sus scrofa Hainan wildboar	China:Hainan
GU135789.1	Sus scrofa Hainan wildboar	China:Hainan
GU135790.1	Sus scrofa Jiangxi wildbaor	China:Jiangxi
GU135791.1	Sus scrofa Northeast wildboar	China:Heilongjiang
GU135792.1	Sus scrofa Northeast wildboar	China:Heilongjiang
GU135793.1	Sus scrofa Northeast wildboar	China:Heilongjiang
GU135794.1	Sus scrofa Northeast wildboar	China:Heilongjiang
GU135805.1	Sus scrofa Taizhou wildboar	China:Zhejiang
GU135806.1	Sus scrofa Xiangshan wildboar	China:Zhejiang
GU135807.1	Sus scrofa Northeast wildboar	China:Heilongjiang
GU135808.1	Sus scrofa Northeast wildboar	China:Heilongjiang
GU135809.1	Sus scrofa Hainan wildboar	China:Hainan
GU135810.1	Sus scrofa Hainan wildboar	China:Hainan
GU135811.1	Sus scrofa Hainan wildboar	China:Hainan
GU135813.1	Sus scrofa Hainan wildboar	China:Hainan
GU135817.1	Sus scrofa Hainan wildboar	China:Hainan
GU135818.1	Sus scrofa Hainan wildboar	China:Hainan
GU135819.1	Sus scrofa Hainan wildboar	China:Hainan
GU135820.1	Sus scrofa Hainan wildboar	China:Hainan
GU135821.1	Sus scrofa Jiangshan wildboar	China:Zhejiang
GU135822.1	Sus scrofa Jiangshan wildboar	China:Zhejiang
GU135823.1	Sus scrofa Jiangxi wildbaor	China:Jiangxi
GU135824.1	Sus scrofa Jiangxi wildbaor	China:Jiangxi

GU135825.1	Sus scrofa Jiangxi wildbaor	China:Jiangxi
GU135826.1	Sus scrofa Lin-An wildboar	China:Zhejiang
GU135828.1	Sus scrofa Zhejiang wildboar	China:Zhejiang
GU135829.1	Sus scrofa Zhejiang wildboar	China:Zhejiang
GU135830.1	Sus scrofa Zhejiang wildboar	China:Zhejiang
GU135831.1	Sus scrofa Zhejiang wildboar	China:Zhejiang
GU135834.1	Sus scrofa Zhejiang wildboar	China:Zhejiang
GU135835.1	Sus scrofa Zhejiang wildboar	China:Zhejiang
GU135836.1	Sus scrofa Zhejiang wildboar	China:Zhejiang
GU135837.1	Sus scrofa Zhejiang wildboar	China:Zhejiang
GU147934.1	Sus scrofa taiwanesis pig	China:Taiwan
JN709898.1	Sus scrofa Lonrung	Vietnam
KC250273.1	Sus scrofa Xiang	China:Guangdong
KC250274.1	Sus scrofa Lantang	China:Guangdong
NC_014692.1	Sus scrofa taiwanesis	China:Taiwan
Z50087.1	Sus scrofa leucomystax	Japan
AF486867.1	Sus scrofa Wuzhishan	China:Hainan
GU135709.1	Sus scrofa Lingao	China:Hainan
GU135710.1	Sus scrofa Lingao	China:Hainan
GU135713.1	Sus scrofa Lantang	China:Guangdong
GU135714.1	Sus scrofa Lantang	China:Guangdong
GU135768.1	Sus scrofa Daweizi	China:Hunan
GU135772.1	Sus scrofa Dongshan	China:Guangxi
GU135783.1	Sus scrofa Bama	China:Guangxi
AM492573.1	Sus scrofa	Indonesia:Manokwari-West New Guinea
AM492574.1	Sus scrofa	Indonesia:Manokwari-West New Guinea
AY237530.1	Sus scrofa Meishan	China
AY237531.1	Sus scrofa Meishan	China
AY830179.1	Sus scrofa Jeju native	South Korea
DQ444704.1	Sus scrofa wild boar	Laos
EF545568.1	Sus scrofa wild boar	China:Yunnan
EU090703.1	Sus scrofa Korean wild boar	South Korea
cCN1	ancient pig	China:Qinghai, Changning site
cCN2	ancient pig	China:Qinghai, Changning site
cCS1	ancient pig	China:Hebei, Cishan site
cCS2	ancient pig	China:Hebei, Cishan site
cCS3	ancient pig	China:Hebei, Cishan site
cDSQ1	ancient pig	China:Inner Mongolia, Dashanqian site
cDSQ2	ancient pig	China:Inner Mongolia, Dashanqian site
cGCZ1	ancient pig	China:Henan, Guchengzhai site
cGCZ2	ancient pig	China:Henan, Guchengzhai site
cGH1	ancient pig	China:Shanxi, Gaohong site
cGH2	ancient pig	China:Shanxi, Gaohong site
cGH3	ancient pig	China:Shanxi, Gaohong site
cGH4	ancient pig	China:Shanxi, Gaohong site
cGH5	ancient pig	China:Shanxi, Gaohong site
cGH6	ancient pig	China:Shanxi, Gaohong site
cJH1	ancient pig	China:Henan, Jiahu site
cJH2	ancient pig	China:Henan, Jiahu site
cLJ1	ancient pig	China:Qinghai, Lajia site
cLJ2	ancient pig	China:Qinghai, Lajia site
cLJ3	ancient pig	China:Qinghai, Lajia site
cLJ4	ancient pig	China:Qinghai, Lajia site
cNW1	ancient pig	China:Henan, Nanwa site

		cNW2	ancient pig	China:Henan, Nanwa site
		cNZT1	ancient pig	China:Hebei, Nanzhuangtou site
		cNZT2	ancient pig	China:Hebei, Nanzhuangtou site
		cNZT3	ancient pig	China:Hebei, Nanzhuangtou site
		cQLQ1	ancient pig	China:Hubei, Qinglongquan site
		cQLQ2	ancient pig	China:Hubei, Qinglongquan site
		cQLQ3	ancient pig	China:Hubei, Qinglongquan site
		cQLQ7	ancient pig	China:Hubei, Qinglongquan site
		cTS1	ancient pig	China:Shanxi, Taosi site
		cTS2	ancient pig	China:Shanxi, Taosi site
		cTS3	ancient pig	China:Shanxi, Taosi site
		cTS4	ancient pig	China:Shanxi, Taosi site
		cWCG1	ancient pig	China:Henan, Wangchenggang site
		cWD1	ancient pig	China:Henan, Wadian site
		cWD2	ancient pig	China:Henan, Wadian site
		cWD3	ancient pig	China:Henan, Wadian site
		cXLW1	ancient pig	China:Inner Mongolia, Xinglongwa site
		cXLW2	ancient pig	China:Inner Mongolia, Xinglongwa site
		cXLW3	ancient pig	China:Inner Mongolia, Xinglongwa site
2	2	AB015074.1	Sus scrofa Ryukyu wild boar	Japan:Ryukyu
		AB015075.1	Sus scrofa Ryukyu wild boar	Japan:Ryukyu
3	11	AB015078.1	Sus scrofa Ohmini miniature pig	Japan
		AM492637.1	Sus scrofa	Indonesia:Bandung-Java
		AM492640.1	Sus scrofa	Indonesia:Bandung-Java
		AY634180.1	Sus scrofa Korean wild boar	South Korea
		EU979221.1	Sus scrofa Luyan white	China:Shandong
		GU135832.1	Sus scrofa Zhejiang wildboar	China:Zhejiang
		GU135833.1	Sus scrofa Zhejiang wildboar	China:Zhejiang
		cQLQ4	ancient pig	China:Hubei, Qinglongquan site
		cQLQ5	ancient pig	China:Hubei, Qinglongquan site
		cQLQ6	ancient pig	China:Hubei, Qinglongquan site
		cWBP2	ancient pig	China:Jilin, Wangbabozi site
4	1	AB376964.1	Sus scrofa domesticus	Thailand:Kanchanaburi
5	2	AM492611.1	Sus scrofa	Indonesia:Mukuwashi-Flores
		AM492612.1	Sus scrofa	Indonesia:Mukuwashi-Flores
6	5	AY830173.1	Sus scrofa Jeju native	South Korea
		AY830176.1	Sus scrofa Jeju native	South Korea
		AY830177.1	Sus scrofa Jeju native	South Korea
		AY830178.1	Sus scrofa Jeju native	South Korea
		AY830183.1	Sus scrofa Jeju native	South Korea
7	1	AY692029.1	Sus scrofa Korean wild boar	South Korea
8	3	AY692032.1	Sus scrofa Korean wild boar	South Korea
		AY830159.1	Sus scrofa coreanus	South Korea
		DQ268530.1	Sus scrofa Korean wild boar	South Korea
9	1	AY743589.1	Sus scrofa Indian wild boar	India
10	1	AY830161.1	Sus scrofa coreanus	South Korea
11	2	AY830162.1	Sus scrofa coreanus	South Korea
		DQ207753.1	Sus scrofa Korean wild boar	South Korea
12	1	AY830168.1	Sus scrofa coreanus	South Korea
13	1	AY830174.1	Sus scrofa Jeju native	South Korea
14	1	AY830175.1	Sus scrofa Jeju native	South Korea
15	1	EF545592.1	Sus scrofa wild boar	Malaysia
16	1	DQ534707.2	Sus scrofa Taoyuan pig	China:Taiwan
17	1	GU135653.1	Sus scrofa Jianli	China:Hubei

18	8	GU135661.1	Sus scrofa Jiangquhai	China:Jiangsu
		GU135719.1	Sus scrofa Wei	China:Anhui
		GU135720.1	Sus scrofa Wei	China:Anhui
		GU135770.1	Sus scrofa Luchuan	China:Guangxi
		GU135776.1	Sus scrofa Guizhong	China:Guangxi
		cNW3	ancient pig	China:Henan, Nanwa site
		cQLQ8	ancient pig	China:Hubei, Qinglongquan site
		cQLQ9	ancient pig	China:Hubei, Qinglongquan site
19	8	GU135725.1	Sus scrofa Mashen	China:Shanxi Datong
		GU135726.1	Sus scrofa Mashen	China:Shanxi Datong
		GU135748.1	Sus scrofa Erhualian	China:Jiangsu
		GU135812.1	Sus scrofa Hainan wildboar	China:Hainan
		GU135814.1	Sus scrofa Hainan wildboar	China:Hainan
		GU135815.1	Sus scrofa Hainan wildboar	China:Hainan
		GU135816.1	Sus scrofa Hainan wildboar	China:Hainan
		GU135827.1	Sus scrofa Mashen	China:Shanxi Datong
20	1	GU135784.1	Sus scrofa Zang	China:Tibet
21	1	cWBP1	ancient pig	China:Jinlin, Wangbabozi site

Table S8. The mtDNA control region sequences used in this analysis.

Haplotype label	Haplotype size	Sequence label	Breeed/Status	Distribution	Region
1	8	AB041466.1	Wild boar	Yunnan	Southwest China
		AY751460.1	Wild boar	Northeast China	Northeast China
		DQ496744.1	Wild boar	Northeast China	Northeast China
		DQ496753.1	Wild boar	Northeast China	Northeast China
		DQ496769.1	Wild boar	Northeast China	Northeast China
		DQ496771.1	Wild boar	Northeast China	Northeast China
		DQ496772.1	Wild boar	Northeast China	Northeast China
		EF545580.1	Wild boar	Jiangxi	Southeast China
2	423	AB041475.1	Jinhua	Zhejiang	Southeast China
		AB041476.1	Jinhua	Zhejiang	Southeast China
		AB041477.1	Jinhua	Zhejiang	Southeast China
		AF276923.1	Tongcheng	Hubei	Southeast China
		AF276925.1	Wannan Spotted	Anhui	Southeast China
		AM040649.1	Meishan	Taiwan	Southeast China
		AY178253.1	Wild boar	Yunnan	Southwest China
		AY178261.1	Wild boar	Yunnan	Southwest China
		AY463061.1	Ganzhongnan Spotted	Jiangxi	Southeast China
		AY884642.1	Wild boar	Gansu	Northwest China
		DQ152870.2	Leping Spotted	Jiangxi	Southeast China
		DQ152871.2	Yushan Black	Jiangxi	Southeast China
		DQ152880.2	Yimeng Black	Shandong	North China
		DQ152883.2	Yimeng Black	Shandong	North China
		DQ152891.2	Xiang	Guizhou	Southwest China
		DQ152893.2	Xiang	Guizhou	Southwest China
		DQ379105.2	Jiaozhou Black	Shandong	North China
		DQ379106.2	Jiaozhou Black	Shandong	North China
		DQ379107.2	Jiaozhou Black	Shandong	North China
		DQ379108.2	Jiaozhou Black	Shandong	North China
		DQ379109.2	Jiaozhou Black	Shandong	North China
		DQ379110.2	Leping Spotted	Jiangxi	Southeast China
		DQ379111.2	Leping Spotted	Jiangxi	Southeast China
		DQ379112.2	Quanbei	Guizhou	Southwest China
		DQ379113.2	Shanggao	Jiangxi	Southeast China
		DQ379114.2	Shengxian Spotted	Zhejiang	Southeast China
		DQ379115.2	Tongcheng	Hubei	Southeast China
		DQ379116.2	Xiang	Guizhou	Southwest China
		DQ379130.2	Yushan Black	Jiangxi	Southeast China
		DQ379165.2	Laiwu Black	Shandong	North China
		DQ379166.2	Laiwu Black	Shandong	North China
		DQ379167.2	Laiwu Black	Shandong	North China
		DQ379168.2	Laiwu Black	Shandong	North China
		DQ379169.2	Min	Northeast China	Northeast China
		DQ379170.2	Min	Northeast China	Northeast China
		DQ379171.2	Min	Northeast China	Northeast China
		DQ379172.2	Min	Northeast China	Northeast China
		DQ379173.2	Min	Northeast China	Northeast China
		DQ379175.2	Yimeng Black	Shandong	North China
		DQ379176.2	Jinhua	Zhejiang	Southeast China

DQ379177.2	Jinhua	Zhejiang	Southeast China
DQ379200.2	Jiangquhai	Jiangsu	Southeast China
DQ379201.2	Xiang	Guizhou	Southwest China
DQ496251.1	Aba	Sichuan	Plateau
DQ496257.1	Aba	Sichuan	Plateau
DQ496258.1	Aba	Sichuan	Plateau
DQ496259.1	Aba	Sichuan	Plateau
DQ496261.1	Aba	Sichuan	Plateau
DQ496268.1	Aba	Sichuan	Plateau
DQ496274.1	Bihu	Zhejiang	Southeast China
DQ496276.1	Bihu	Zhejiang	Southeast China
DQ496282.1	Bamei	Qinghai	Northwest China
DQ496283.1	Bamei	Qinghai	Northwest China
DQ496284.1	Bamei	Qinghai	Northwest China
DQ496285.1	Bamei	Qinghai	Northwest China
DQ496286.1	Bamei	Qinghai	Northwest China
DQ496288.1	Bamei	Qinghai	Northwest China
DQ496289.1	Bamei	Qinghai	Northwest China
DQ496291.1	Bamei	Qinghai	Northwest China
DQ496314.1	Baoshan	Yunnan	Southwest China
DQ496328.1	Chalu	Zhejiang	Southeast China
DQ496329.1	Chalu	Zhejiang	Southeast China
DQ496334.1	Chalu	Zhejiang	Southeast China
DQ496343.1	Dahuabai	Guangdong	Southeast China
DQ496344.1	Dahuabai	Guangdong	Southeast China
DQ496345.1	Dahuabai	Guangdong	Southeast China
DQ496346.1	Dahuabai	Guangdong	Southeast China
DQ496347.1	Dahuabai	Guangdong	Southeast China
DQ496348.1	Dahuabai	Guangdong	Southeast China
DQ496349.1	Dahuabai	Guangdong	Southeast China
DQ496350.1	Dahuabai	Guangdong	Southeast China
DQ496351.1	Dahuabai	Guangdong	Southeast China
DQ496352.1	Dahuabai	Guangdong	Southeast China
DQ496353.1	Dahuabai	Guangdong	Southeast China
DQ496354.1	Dahuabai	Guangdong	Southeast China
DQ496355.1	Dahuabai	Guangdong	Southeast China
DQ496356.1	Dahuabai	Guangdong	Southeast China
DQ496361.1	Dahe	Yunnan	Southwest China
DQ496364.1	Dahuabai	Guangdong	Southeast China
DQ496377.1	Diannan small-ear	Yunnan	Southwest China
DQ496400.1	Zang	Yunnan	Plateau
DQ496406.1	Zang	Yunnan	Plateau
DQ496407.1	Zang	Yunnan	Plateau
DQ496410.1	Erhualian	Jiangsu	Southeast China
DQ496462.1	Huzhu	Qinghai	Plateau
DQ496477.1	Jianli	Hubei	Southeast China
DQ496479.1	Jianli	Hubei	Southeast China
DQ496480.1	Jiaxing Black	Zhejiang	Southeast China
DQ496481.1	Jiaxing Black	Zhejiang	Southeast China
DQ496482.1	Jiaxing Black	Zhejiang	Southeast China
DQ496484.1	Jiaxing Black	Zhejiang	Southeast China

DQ496486.1	Jiaxing Black	Zhejiang	Southeast China
DQ496492.1	Jiangquhai	Jiangsu	Southeast China
DQ496493.1	Jiangquhai	Jiangsu	Southeast China
DQ496494.1	Jiangquhai	Jiangsu	Southeast China
DQ496496.1	Jiangquhai	Jiangsu	Southeast China
DQ496499.1	Jiaozhou Black	Shandong	North China
DQ496500.1	Jiaozhou Black	Shandong	North China
DQ496501.1	Jiaozhou Black	Shandong	North China
DQ496502.1	Jiaozhou Black	Shandong	North China
DQ496503.1	Jiaozhou Black	Shandong	North China
DQ496504.1	Jiaozhou Black	Shandong	North China
DQ496505.1	Jiaozhou Black	Shandong	North China
DQ496506.1	Jiaozhou Black	Shandong	North China
DQ496507.1	Jiaozhou Black	Shandong	North China
DQ496508.1	Jiaozhou Black	Shandong	North China
DQ496509.1	Jiaozhou Black	Shandong	North China
DQ496510.1	Jiaozhou Black	Shandong	North China
DQ496511.1	Jiaozhou Black	Shandong	North China
DQ496533.1	Leping Spotted	Jiangxi	Southeast China
DQ496534.1	Leping Spotted	Jiangxi	Southeast China
DQ496535.1	Leping Spotted	Jiangxi	Southeast China
DQ496536.1	Leping Spotted	Jiangxi	Southeast China
DQ496539.1	Leping Spotted	Jiangxi	Southeast China
DQ496541.1	Laiwu Black	Shandong	North China
DQ496542.1	Laiwu Black	Shandong	North China
DQ496543.1	Laiwu Black	Shandong	North China
DQ496544.1	Laiwu Black	Shandong	North China
DQ496545.1	Laiwu Black	Shandong	North China
DQ496546.1	Laiwu Black	Shandong	North China
DQ496547.1	Laiwu Black	Shandong	North China
DQ496554.1	Min	Northeast China	Northeast China
DQ496557.1	Min	Northeast China	Northeast China
DQ496558.1	Min	Northeast China	Northeast China
DQ496559.1	Min	Northeast China	Northeast China
DQ496560.1	Min	Northeast China	Northeast China
DQ496562.1	Min	Northeast China	Northeast China
DQ496563.1	Min	Northeast China	Northeast China
DQ496565.1	Min	Northeast China	Northeast China
DQ496570.1	Min	Northeast China	Northeast China
DQ496571.1	Min	Northeast China	Northeast China
DQ496576.1	Mingguang small-ear	Yunnan	Southwest China
DQ496577.1	Mingguang small-ear	Yunnan	Southwest China
DQ496578.1	Mingguang small-ear	Yunnan	Southwest China
DQ496581.1	Meishan	Zhejiang	Southeast China
DQ496600.1	Putian	Fujian	Southeast China
DQ496603.1	Putian	Fujian	Southeast China
DQ496604.1	Putian	Fujian	Southeast China
DQ496605.1	Putian	Fujian	Southeast China
DQ496609.1	Qianbei Black	Guizhou	Southwest China
DQ496617.1	Qianbei Black	Guizhou	Southwest China
DQ496624.1	Qingping	Hubei	Southeast China

		DQ496625.1	Qingping	Hubei	Southeast China
		DQ496626.1	Qingping	Hubei	Southeast China
		DQ496629.1	Qingping	Hubei	Southeast China
		DQ496635.1	Qingping	Hubei	Southeast China
		DQ496638.1	Qingping	Hubei	Southeast China
		DQ496646.1	Rongchang	Chongqing	Southwest China
		DQ496648.1	Rongchang	Chongqing	Southwest China
		DQ496650.1	Rongchang	Chongqing	Southwest China
		DQ496668.1	Saba	Yunnan	Southwest China
		DQ496696.1	Meishan	Jiangxi	Southeast China
		DQ496699.1	Meishan	Jiangxi	Southeast China
		DQ496700.1	Meishan	Jiangxi	Southeast China
		DQ496702.1	Meishan	Jiangxi	Southeast China
		DQ496703.1	Meishan	Jiangxi	Southeast China
		DQ496704.1	Meishan	Jiangxi	Southeast China
		DQ496706.1	Shengxian Spotted	Zhejiang	Southeast China
		DQ496708.1	Shengxian Spotted	Zhejiang	Southeast China
		DQ496763.1	Wild boar	Northeast China	Northeast China
		DQ496765.1	Wild boar	Northeast China	Northeast China
		DQ496766.1	Wild boar	Northeast China	Northeast China
		DQ496886.1	Wild boar	Zhejiang	Southeast China
		DQ496887.1	Wild boar	Zhejiang	Southeast China
		DQ496895.1	Wild boar	Zhejiang	Southeast China
		DQ496896.1	Wild boar	Zhejiang	Southeast China
		DQ496900.1	Wild boar	Zhejiang	Southeast China
		DQ496901.1	Wild boar	Zhejiang	Southeast China
		DQ496902.1	Wild boar	Zhejiang	Southeast China
		DQ496903.1	Wild boar	Zhejiang	Southeast China
		DQ496904.1	Wild boar	Zhejiang	Southeast China
		DQ496905.1	Wild boar	Zhejiang	Southeast China
		DQ496908.1	Wild boar	Zhejiang	Southeast China
		DQ496909.1	Wild boar	Zhejiang	Southeast China
		DQ496910.1	Wild boar	Zhejiang	Southeast China
		DQ496911.1	Wild boar	Zhejiang	Southeast China
		DQ496924.1	Wei	Anhui	Southeast China
		DQ496929.1	Wei	Anhui	Southeast China
		DQ496930.1	Wei	Anhui	Southeast China
		DQ496931.1	Wei	Anhui	Southeast China
		DQ496976.1	Yimeng Black	Shandong	North China
		DQ496977.1	Yimeng Black	Shandong	North China
		DQ496978.1	Yimeng Black	Shandong	North China
		DQ496982.1	Yimeng Black	Shandong	North China
		DQ496984.1	Yimeng Black	Shandong	North China
		DQ496985.1	Yimeng Black	Shandong	North China
		DQ496986.1	Yimeng Black	Shandong	North China
		DQ496995.1	Yushan Black	Jiangxi	Southeast China
		DQ496996.1	Yushan Black	Jiangxi	Southeast China
		DQ496997.1	Yushan Black	Jiangxi	Southeast China
		DQ496999.1	Yushan Black	Jiangxi	Southeast China
		DQ497000.1	Yushan Black	Jiangxi	Southeast China
		DQ779415.1	Wild boar	Taiwan	Southeast China

DQ779420.1	Wild boar	Shanghai	Southeast China
EF545583.1	Bamei	Qinghai	Northwest China
EF545591.1	Bihu	Zhejiang	Southeast China
EF590142.1	Luchuan	Guangxi	Southwest China
EF590144.1	Yushan Black	Jiangxi	Southeast China
EF590149.1	Tongcheng	Hubei	Southeast China
EF590153.1	Shawutou	Jiangsu	Southeast China
EF590160.1	Licha Black	Shandong	North China
EF590164.1	Hetao big-ear	Inner Mongolia (middle)	North China
EF590170.1	Dongxiang Spotted	Jiangxi	Southeast China
EF590174.1	Laiwu Black	Shandong	North China
EF590183.1	Min	Northeast China	Northeast China
EF590186.1	Jiashan	Jiangsu	Southeast China
EF590188.1	Chalu	Zhejiang	Southeast China
EU660156.1	Licha Black	Shandong	North China
EU660157.1	Licha Black	Shandong	North China
EU660158.1	Licha Black	Shandong	North China
EU660159.1	Licha Black	Shandong	North China
EU660160.1	Licha Black	Shandong	North China
EU660161.1	Licha Black	Shandong	North China
EU660162.1	Licha Black	Shandong	North China
EU660163.1	Licha Black	Shandong	North China
EU660164.1	Laiwu Black	Shandong	North China
EU660165.1	Laiwu Black	Shandong	North China
EU660166.1	Laiwu Black	Shandong	North China
EU660167.1	Laiwu Black	Shandong	North China
EU660168.1	Laiwu Black	Shandong	North China
EU660169.1	Laiwu Black	Shandong	North China
EU660170.1	Laiwu Black	Shandong	North China
EU660189.1	Yimeng Black	Shandong	North China
EU660190.1	Yimeng Black	Shandong	North China
EU660192.1	Yimeng Black	Shandong	North China
EU660193.1	Yimeng Black	Shandong	North China
EU660194.1	Yimeng Black	Shandong	North China
EU660196.1	Yantai Black	Shandong	North China
EU660197.1	Yantai Black	Shandong	North China
EU660198.1	Yantai Black	Shandong	North China
EU660199.1	Yantai Black	Shandong	North China
EU660200.1	Yantai Black	Shandong	North China
EU660201.1	Yantai Black	Shandong	North China
EU660202.1	Yantai Black	Shandong	North China
EU660204.1	Yantai Black	Shandong	North China
EU660205.1	Yantai Black	Shandong	North China
EU979126.1	Laiwu Black	Shandong	North China
EU979127.1	Laiwu Black	Shandong	North China
EU979128.1	Laiwu Black	Shandong	North China
EU979129.1	Laiwu Black	Shandong	North China
EU979130.1	Laiwu Black	Shandong	North China
EU979131.1	Laiwu Black	Shandong	North China
EU979132.1	Laiwu Black	Shandong	North China
EU979133.1	Laiwu Black	Shandong	North China

EU979140.1	Licha Black	Shandong	North China
EU979141.1	Licha Black	Shandong	North China
EU979142.1	Licha Black	Shandong	North China
EU979143.1	Licha Black	Shandong	North China
EU979144.1	Licha Black	Shandong	North China
EU979145.1	Licha Black	Shandong	North China
EU979146.1	Licha Black	Shandong	North China
EU979147.1	Yimeng Black	Shandong	North China
EU979148.1	Yimeng Black	Shandong	North China
EU979149.1	Yimeng Black	Shandong	North China
EU979150.1	Yimeng Black	Shandong	North China
EU979151.1	Yimeng Black	Shandong	North China
EU979152.1	Yimeng Black	Shandong	North China
EU979154.1	Yimeng Black	Shandong	North China
EU979155.1	Yantai Black	Shandong	North China
EU979156.1	Yantai Black	Shandong	North China
EU979157.1	Yantai Black	Shandong	North China
EU979158.1	Yantai Black	Shandong	North China
EU979159.1	Yantai Black	Shandong	North China
EU979173.1	Wulian Black	Shandong	North China
EU979174.1	Wulian Black	Shandong	North China
EU979176.1	Wulian Black	Shandong	North China
EU979180.1	Wulian Black	Shandong	North China
FJ601390.1	Nanyang Black	Henan	North China
FJ601393.1	Jianli	Hubei	Southeast China
FJ601394.1	Jinhua	Zhejiang	Southeast China
FJ601395.1	Jinhua	Zhejiang	Southeast China
FJ601397.1	Jiaxing Black	Zhejiang	Southeast China
FJ601399.1	Jiangquhai	Jiangsu	Southeast China
FJ601401.1	Shawutou	Jiangsu	Southeast China
FJ601404.1	Hetao Big-ear	Inner Mongolia (middle)	North China
FJ601405.1	Hetao Big-ear	Inner Mongolia (middle)	North China
FJ601412.1	Nancheng Black	Jiangxi	Southeast China
FJ601413.1	Nancheng Black	Jiangxi	Southeast China
FJ601426.1	DongXiang Spotted	Jiangxi	Southeast China
FJ601427.1	DongXiang Spotted	Jiangxi	Southeast China
FJ601434.1	Licha Black	Shandong	North China
FJ601435.1	Licha Black	Shandong	North China
FJ601440.1	Yimeng Black	Shandong	North China
FJ601441.1	Yimeng Black	Shandong	North China
FJ601444.1	Laiwu Black	Shandong	North China
FJ601445.1	Laiwu Black	Shandong	North China
FJ601454.1	Qingping	Hubei	Southeast China
FJ601455.1	Qingping	Hubei	Southeast China
FJ601460.1	Wannan Spotted	Anhui	Southeast China
FJ601461.1	Wannan Spotted	Anhui	Southeast China
FJ601464.1	Tongcheng	Hubei	Southeast China
FJ601467.1	Yangxin	Hubei	Southeast China
FJ601468.1	Min	Heilongjiang	Northeast China
FJ601469.1	Min	Heilongjiang	Northeast China
FJ601474.1	Yushan Black	Jiangxi	Southeast China

		FJ601475.1	Yushan Black	Jiangxi	Southeast China
		FJ601479.1	Putian	Fujian	Southeast China
		FJ601483.1	Huai	Fujian	Southeast China
		FJ601484.1	Huai	Fujian	Southeast China
		FJ601486.1	Erhualian	Jiangsu	Southeast China
		FJ601488.1	Wuzhishan	Hainan	Southeast China
		FJ601489.1	Ju'nan	Shandong	North China
		FJ601493.1	Kele	Guizhou	Southwest China
		FJ601504.1	Daweizi	Hunan	Southeast China
		FJ601505.1	Luchuan	Guangxi	Southwest China
		FJ601509.1	Shangyu Spotted	Zhejiang	Southeast China
		FJ601511.1	Guizhong Spotted	Guizhou	Southwest China
		FJ601520.1	Zang	Tibet	Plateau
		FJ601521.1	Wild boar	Zhejiang	Southeast China
		FJ601526.1	Wild boar	Heilongjiang	Northeast China
		FJ601527.1	Wild boar	Heilongjiang	Northeast China
		HM026671.1	Jinhua	Zhejiang	Southeast China
		HM026674.1	Laiwu Black	Shandong	North China
		HM026675.1	Meishan	Jiangsu	Southeast China
		HM026676.1	Min	Northeast China	Northeast China
		HQ148345.1	Zang	Sichuan	Plateau
		HQ148347.1	Zang	Sichuan	Plateau
		HQ148399.1	Zang	Tibet	Plateau
		HQ148401.1	Zang	Tibet	Plateau
		JX068012.1	Chenghua	Sichuan	Southwest China
		JX068013.1	Chenghua	Sichuan	Southwest China
		JX068014.1	Chenghua	Sichuan	Southwest China
		JX068018.1	Jinhua	Zhejiang	Southeast China
		JX068019.1	Jinhua	Zhejiang	Southeast China
		JX068023.1	Jinhua	Zhejiang	Southeast China
		JX068026.1	Jinhua	Zhejiang	Southeast China
		JX068028.1	Jinhua	Zhejiang	Southeast China
		JX068030.1	Jinhua	Zhejiang	Southeast China
		JX068036.1	Jinhua	Zhejiang	Southeast China
		JX068040.1	Jinhua	Zhejiang	Southeast China
		JX068041.1	Jinhua	Zhejiang	Southeast China
		JX068042.1	Jinhua	Zhejiang	Southeast China
		JX068046.1	Jinhua	Zhejiang	Southeast China
		JX068047.1	Jinhua	Zhejiang	Southeast China
		JX068048.1	Jinhua	Zhejiang	Southeast China
		JX068051.1	Jinhua	Zhejiang	Southeast China
		JX068053.1	Jinhua	Zhejiang	Southeast China
		JX068054.1	Jinhua	Zhejiang	Southeast China
		JX068056.1	Jinhua	Zhejiang	Southeast China
		JX068057.1	Jinhua	Zhejiang	Southeast China
		JX068062.1	Jinhua	Zhejiang	Southeast China
		JX068063.1	Jinhua	Zhejiang	Southeast China
		JX068064.1	Jinhua	Zhejiang	Southeast China
		JX068066.1	Jinhua	Zhejiang	Southeast China
		JX068067.1	Jinhua	Zhejiang	Southeast China
		JX068134.1	Zang	Tibet	Plateau

		JX068137.1	Zang	Tibet	Plateau
		JX068139.1	Zang	Tibet	Plateau
		JX068140.1	Zang	Tibet	Plateau
		JX068262.1	Zang	Yunnan	Plateau
		JX068307.1	Zang	Gansu	Plateau
		JX068308.1	Zang	Gansu	Plateau
		JX068315.1	Zang	Gansu	Plateau
		JX068316.1	Zang	Gansu	Plateau
		JX068317.1	Zang	Gansu	Plateau
		JX068318.1	Zang	Gansu	Plateau
		JX068320.1	Zang	Gansu	Plateau
		JX068321.1	Zang	Gansu	Plateau
		JX068323.1	Zang	Gansu	Plateau
		JX068324.1	Zang	Gansu	Plateau
		JX068325.1	Zang	Gansu	Plateau
		JX068326.1	Zang	Gansu	Plateau
		JX068329.1	Zang	Gansu	Plateau
		JX068332.1	Zang	Gansu	Plateau
		JX068333.1	Zang	Gansu	Plateau
		JX068334.1	Zang	Gansu	Plateau
		JX068340.1	Zang	Gansu	Plateau
		JX068346.1	Zang	Gansu	Plateau
		JX068347.1	Zang	Gansu	Plateau
		JX068350.1	Zang	Gansu	Plateau
		JX068351.1	Zang	Gansu	Plateau
		JX068352.1	Zang	Gansu	Plateau
		JX068353.1	Zang	Gansu	Plateau
		JX068354.1	Zang	Gansu	Plateau
		JX068357.1	Zang	Sichuan	Plateau
		JX068359.1	Zang	Sichuan	Plateau
		JX068361.1	Zang	Sichuan	Plateau
		JX068364.1	Zang	Sichuan	Plateau
		JX068367.1	Zang	Sichuan	Plateau
		JX068378.1	Zang	Sichuan	Plateau
		JX068380.1	Zang	Sichuan	Plateau
		JX068386.1	Zang	Sichuan	Plateau
		JX068391.1	Pengzhou Mountain	Sichuan	Southwest China
		JX068392.1	Pengzhou Mountain	Sichuan	Southwest China
		JX068397.1	Pengzhou Mountain	Sichuan	Southwest China
		JX068398.1	Pengzhou Mountain	Sichuan	Southwest China
		JX068399.1	Pengzhou Mountain	Sichuan	Southwest China
		JX068400.1	Pengzhou Mountain	Sichuan	Southwest China
		JX068406.1	Pengzhou Mountain	Sichuan	Southwest China
		JX068417.1	Pengzhou Mountain	Sichuan	Southwest China
		JX068418.1	Pengzhou Mountain	Sichuan	Southwest China
		JX068420.1	Pengzhou Mountain	Sichuan	Southwest China
		JX068422.1	Pengzhou Mountain	Sichuan	Southwest China
		JX068424.1	Pengzhou Mountain	Sichuan	Southwest China
		JX068427.1	Pengzhou Mountain	Sichuan	Southwest China
		JX068428.1	Pengzhou Mountain	Sichuan	Southwest China
		JX068429.1	Pengzhou Mountain	Sichuan	Southwest China

		JX068436.1	Pengzhou Mountain	Sichuan	Southwest China
		JX068437.1	Pengzhou Mountain	Sichuan	Southwest China
		JX068448.1	Wujin	Sichuan	Southwest China
		JX068451.1	Wujin	Sichuan	Southwest China
		JX068453.1	Wujin	Sichuan	Southwest China
		JX068456.1	Wujin	Sichuan	Southwest China
		JX068461.1	Wujin	Sichuan	Southwest China
		JX068471.1	Wujin	Sichuan	Southwest China
		JX068472.1	Wujin	Sichuan	Southwest China
		JX068475.1	Wujin	Sichuan	Southwest China
		JX068476.1	Wujin	Sichuan	Southwest China
		JX068482.1	Wujin	Sichuan	Southwest China
		JX068504.1	Yanan	Sichuan	Southwest China
		JX068508.1	Yanan	Sichuan	Southwest China
		JX068517.1	Yanan	Sichuan	Southwest China
		KC493608.1	Zang	Sichuan	Plateau
		KC493609.1	Zang	Sichuan	Plateau
		KC505408.1	Wujin	Sichuan	Southwest China
		KF472179.1	Daweizi	Hunan	Southeast China
		dNZT1	ancient pig	Hebei	North China
		dNZT2	ancient pig	Hebei	North China
		dNZT3	ancient pig	Hebei	North China
		dQLQ3	ancient pig	Hubei	Southeast China
		dQLQ4	ancient pig	Hubei	Southeast China
		dTS3	ancient pig	Shanxi	North China
		dWCG1	ancient pig	Henan	North China
3	286	AF276922.1	Erhualian	Jiangsu	Southeast China
		AF276928.1	Gandong Black	Jiangxi	Southeast China
		AM040650.1	Meishan	Taiwan	Southeast China
		AM040651.1	Meishan	Taiwan	Southeast China
		AM040652.1	Meishan	Taiwan	Southeast China
		AY230818.1	Erhualian	Jiangsu	Southeast China
		AY230824.1	Erhualian	Jiangsu	Southeast China
		AY230826.1	Erhualian	Jiangsu	Southeast China
		DQ152872.2	Neijiang	Sichuan	Southwest China
		DQ152873.2	Yushan Black	Jiangxi	Southeast China
		DQ152877.2	Shengxian Spotted	Zhejiang	Southeast China
		DQ379131.2	Neijiang	Sichuan	Southwest China
		DQ379132.2	Neijiang	Sichuan	Southwest China
		DQ379133.2	Jiangquhai	Jiangsu	Southeast China
		DQ379134.2	Leping Spotted	Jiangxi	Southeast China
		DQ379135.2	Quanbei	Guizhou	Southwest China
		DQ379136.2	Shengxian Spotted	Zhejiang	Southeast China
		DQ379137.2	Shengxian Spotted	Zhejiang	Southeast China
		DQ379138.2	Tongcheng	Hubei	Southeast China
		DQ379139.2	Tongcheng	Hubei	Southeast China
		DQ379140.2	Xiangxi Black	Hunan	Southeast China
		DQ379141.2	Yushan Black	Jiangxi	Southeast China
		DQ379142.2	Neijiang	Sichuan	Southwest China
		DQ379143.2	Neijiang	Sichuan	Southwest China
		DQ379152.2	Shengxian Spotted	Zhejiang	Southeast China

		DQ466081.2	Nuogu	Guizhou	Southwest China
		DQ496260.1	Aba	Sichuan	Plateau
		DQ496263.1	Aba	Sichuan	Plateau
		DQ496275.1	Bihu	Zhejiang	Southeast China
		DQ496277.1	Bihu	Zhejiang	Southeast China
		DQ496278.1	Bihu	Zhejiang	Southeast China
		DQ496279.1	Bihu	Zhejiang	Southeast China
		DQ496280.1	Bihu	Zhejiang	Southeast China
		DQ496303.1	Wild boar	Yunnan	Southwest China
		DQ496304.1	Wild boar	Yunnan	Southwest China
		DQ496306.1	Diannan small-ear	Yunnan	Southwest China
		DQ496315.1	Baoshan	Yunnan	Southwest China
		DQ496322.1	Baoshan	Yunnan	Southwest China
		DQ496323.1	Baoshan	Yunnan	Southwest China
		DQ496363.1	Dahe	Yunnan	Southwest China
		DQ496399.1	Zang	Yunnan	Plateau
		DQ496401.1	Zang	Yunnan	Plateau
		DQ496404.1	Zang	Yunnan	Plateau
		DQ496413.1	Erhualian	Jiangsu	Southeast China
		DQ496414.1	Erhualian	Jiangsu	Southeast China
		DQ496421.1	Erhualian	Jiangsu	Southeast China
		DQ496422.1	Erhualian	Jiangsu	Southeast China
		DQ496437.1	Gaopo	Guizhou	Southwest China
		DQ496440.1	Hanjiang Black	Shaanxi	North China
		DQ496443.1	Hanjiang Black	Shaanxi	North China
		DQ496459.1	Huzhu	Qinghai	Plateau
		DQ496460.1	Huzhu	Qinghai	Plateau
		DQ496461.1	Huzhu	Qinghai	Plateau
		DQ496466.1	Huzhu	Qinghai	Plateau
		DQ496487.1	Jiaxing Black	Zhejiang	Southeast China
		DQ496488.1	Jinhua	Zhejiang	Southeast China
		DQ496514.1	Lantang	Guangdong	Southeast China
		DQ496515.1	Lantang	Guangdong	Southeast China
		DQ496521.1	Lantang	Guangdong	Southeast China
		DQ496522.1	Lantang	Guangdong	Southeast China
		DQ496527.1	Lantang	Guangdong	Southeast China
		DQ496528.1	Lantang	Guangdong	Southeast China
		DQ496538.1	Leping Spotted	Jiangxi	Southeast China
		DQ496540.1	Leping Spotted	Jiangxi	Southeast China
		DQ496582.1	Neijiang	Sichuan	Southwest China
		DQ496583.1	Neijiang	Sichuan	Southwest China
		DQ496584.1	Neijiang	Sichuan	Southwest China
		DQ496585.1	Neijiang	Sichuan	Southwest China
		DQ496586.1	Neijiang	Sichuan	Southwest China
		DQ496587.1	Neijiang	Sichuan	Southwest China
		DQ496588.1	Neijiang	Sichuan	Southwest China
		DQ496589.1	Neijiang	Sichuan	Southwest China
		DQ496590.1	Neijiang	Sichuan	Southwest China
		DQ496591.1	Neijiang	Sichuan	Southwest China
		DQ496592.1	Neijiang	Sichuan	Southwest China
		DQ496593.1	Neijiang	Sichuan	Southwest China

DQ496594.1	Neijiang	Sichuan	Southwest China
DQ496595.1	Neijiang	Sichuan	Southwest China
DQ496596.1	Neijiang	Sichuan	Southwest China
DQ496601.1	Putian	Fujian	Southeast China
DQ496602.1	Putian	Fujian	Southeast China
DQ496627.1	Qingping	Hubei	Southeast China
DQ496634.1	Qingping	Hubei	Southeast China
DQ496637.1	Qingping	Hubei	Southeast China
DQ496647.1	Rongchang	Chongqing	Southwest China
DQ496652.1	Rongchang	Chongqing	Southwest China
DQ496653.1	Rongchang	Chongqing	Southwest China
DQ496659.1	Saba	Yunnan	Southwest China
DQ496705.1	Shengxian Spotted	Zhejiang	Southeast China
DQ496707.1	Shengxian Spotted	Zhejiang	Southeast China
DQ496737.1	Wannan Spotted	Anhui	Southeast China
DQ496777.1	Wild boar	Fujian	Southeast China
DQ496783.1	Wild boar	Yunnan	Southwest China
DQ496787.1	Wild boar	Fujian	Southeast China
DQ496816.1	Wild boar	Jiangxi	Southeast China
DQ496826.1	Wild boar	Guizhou	Southwest China
DQ496829.1	Wild boar	Guizhou	Southwest China
DQ496862.1	Wild boar	Yunnan	Southwest China
DQ496871.1	Wild boar	Yunnan	Southwest China
DQ496872.1	Wild boar	Yunnan	Southwest China
DQ496881.1	Wild boar	Yunnan	Southwest China
DQ496883.1	Wild boar	Yunnan	Southwest China
DQ496884.1	Wild boar	Yunnan	Southwest China
DQ496907.1	Wild boar	Zhejiang	Southeast China
DQ496912.1	Wild boar	Zhejiang	Southeast China
DQ496921.1	Wei	Anhui	Southeast China
DQ496922.1	Wei	Anhui	Southeast China
DQ496923.1	Wei	Anhui	Southeast China
DQ496928.1	Wei	Anhui	Southeast China
DQ496971.1	Yahe	Sichuan	Southwest China
DQ496974.1	Yahe	Sichuan	Southwest China
DQ496987.1	Yushan Black	Jiangxi	Southeast China
DQ496988.1	Yushan Black	Jiangxi	Southeast China
DQ496991.1	Yushan Black	Jiangxi	Southeast China
DQ496992.1	Yushan Black	Jiangxi	Southeast China
DQ496993.1	Yushan Black	Jiangxi	Southeast China
DQ496998.1	Yushan Black	Jiangxi	Southeast China
DQ779416.1	Wild boar	Taiwan	Southeast China
DQ779417.1	Wild boar	Taiwan	Southeast China
DQ972936.3	Lanyu	Taiwan	Southeast China
EF545569.1	Wild boar	Yunnan	Southwest China
EF545588.1	Huzhu	Qinghai	Plateau
EF590156.1	Ningxiang	Hunan	Southeast China
EF590157.1	Mashen	Shanxi	North China
EF590158.1	Longlin	Guangxi	Southwest China
EF590159.1	Lingao	Hainan	Southeast China
EF590161.1	Lantang	Guangdong	Southeast China

		EF590190.1	Jinhua	Zhejiang	Southeast China
		EU008085.1	Wild boar	Taiwan	Southeast China
		EU008086.1	Wild boar	Taiwan	Southeast China
		EU008087.1	Wild boar	Taiwan	Southeast China
		EU979166.1	Wulian Black	Shandong	North China
		EU979167.1	Wulian Black	Shandong	North China
		EU979168.1	Wulian Black	Shandong	North China
		EU979169.1	Wulian Black	Shandong	North China
		EU979170.1	Wulian Black	Shandong	North China
		EU979172.1	Wulian Black	Shandong	North China
		EU979177.1	Wulian Black	Shandong	North China
		EU979181.1	Wulian Black	Shandong	North China
		FJ156279.1	Wild boar	Taiwan	Southeast China
		FJ601406.1	Yanan	Sichuan	Southwest China
		FJ601408.1	Neijiang	Sichuan	Southwest China
		FJ601409.1	Neijiang	Sichuan	Southwest China
		FJ601411.1	Xingzi Black	Jiangxi	Southeast China
		FJ601418.1	Small Meishan	Jiangsu	Southeast China
		FJ601419.1	Small Meishan	Jiangsu	Southeast China
		FJ601430.1	Rongchang	Chongqing	Southwest China
		FJ601436.1	Hanjiang Black	Shaanxi	North China
		FJ601446.1	Lingao	Hainan	Southeast China
		FJ601450.1	Lantang	Guangdong	Southeast China
		FJ601451.1	Lantang	Guangdong	Southeast China
		FJ601462.1	Mashen	Shanxi	North China
		FJ601463.1	Mashen	Shanxi	North China
		FJ601465.1	Tongcheng	Hubei	Southeast China
		FJ601478.1	Shaziling	Hunan	Southeast China
		FJ601482.1	Wuyi Black	Fujian	Southeast China
		FJ601485.1	Erhualian	Jiangsu	Southeast China
		FJ601492.1	Taoyuan	Taiwan	Southeast China
		FJ601494.1	Kele	Guizhou	Southwest China
		FJ601496.1	Chenghua	Sichuan	Southwest China
		FJ601497.1	Longlin	Guangxi	Southwest China
		FJ601502.1	Donglan	Guangxi	Southwest China
		FJ601507.1	Donglan	Guangxi	Southwest China
		FJ601510.1	Shangyu Spotted	Zhejiang	Southeast China
		FJ601516.1	Exi Black	Hubei	Southeast China
		FJ601518.1	Bamaxiang	Guangxi	Southwest China
		GQ220329.1	Dahe	Yunnan	Southwest China
		HM026677.1	Neijiang	Sichuan	Southwest China
		HQ148330.1	Zang	Tibet	Plateau
		HQ148333.1	Zang	Tibet	Plateau
		HQ148427.1	Zang	Yunnan	Plateau
		HQ148430.1	Zang	Yunnan	Plateau
		JX068015.1	Chenghua	Sichuan	Southwest China
		JX068035.1	Jinhua	Zhejiang	Southeast China
		JX068039.1	Jinhua	Zhejiang	Southeast China
		JX068052.1	Jinhua	Zhejiang	Southeast China
		JX068060.1	Jinhua	Zhejiang	Southeast China
		JX068065.1	Jinhua	Zhejiang	Southeast China

		JX068068.1	Neijiang	Sichuan	Southwest China
		JX068069.1	Neijiang	Sichuan	Southwest China
		JX068070.1	Neijiang	Sichuan	Southwest China
		JX068071.1	Neijiang	Sichuan	Southwest China
		JX068072.1	Neijiang	Sichuan	Southwest China
		JX068073.1	Neijiang	Sichuan	Southwest China
		JX068075.1	Neijiang	Sichuan	Southwest China
		JX068076.1	Neijiang	Sichuan	Southwest China
		JX068077.1	Neijiang	Sichuan	Southwest China
		JX068078.1	Neijiang	Sichuan	Southwest China
		JX068079.1	Neijiang	Sichuan	Southwest China
		JX068080.1	Neijiang	Sichuan	Southwest China
		JX068081.1	Neijiang	Sichuan	Southwest China
		JX068082.1	Neijiang	Sichuan	Southwest China
		JX068083.1	Neijiang	Sichuan	Southwest China
		JX068084.1	Neijiang	Sichuan	Southwest China
		JX068085.1	Neijiang	Sichuan	Southwest China
		JX068087.1	Neijiang	Sichuan	Southwest China
		JX068088.1	Neijiang	Sichuan	Southwest China
		JX068089.1	Neijiang	Sichuan	Southwest China
		JX068090.1	Neijiang	Sichuan	Southwest China
		JX068091.1	Neijiang	Sichuan	Southwest China
		JX068092.1	Neijiang	Sichuan	Southwest China
		JX068093.1	Neijiang	Sichuan	Southwest China
		JX068094.1	Neijiang	Sichuan	Southwest China
		JX068096.1	Neijiang	Sichuan	Southwest China
		JX068097.1	Neijiang	Sichuan	Southwest China
		JX068098.1	Neijiang	Sichuan	Southwest China
		JX068099.1	Neijiang	Sichuan	Southwest China
		JX068100.1	Neijiang	Sichuan	Southwest China
		JX068101.1	Neijiang	Sichuan	Southwest China
		JX068102.1	Neijiang	Sichuan	Southwest China
		JX068103.1	Neijiang	Sichuan	Southwest China
		JX068104.1	Neijiang	Sichuan	Southwest China
		JX068105.1	Neijiang	Sichuan	Southwest China
		JX068106.1	Neijiang	Sichuan	Southwest China
		JX068108.1	Neijiang	Sichuan	Southwest China
		JX068109.1	Neijiang	Sichuan	Southwest China
		JX068110.1	Neijiang	Sichuan	Southwest China
		JX068112.1	Neijiang	Sichuan	Southwest China
		JX068113.1	Neijiang	Sichuan	Southwest China
		JX068114.1	Neijiang	Sichuan	Southwest China
		JX068115.1	Neijiang	Sichuan	Southwest China
		JX068116.1	Neijiang	Sichuan	Southwest China
		JX068153.1	Zang	Tibet	Plateau
		JX068200.1	Zang	Tibet	Plateau
		JX068202.1	Zang	Tibet	Plateau
		JX068206.1	Zang	Tibet	Plateau
		JX068208.1	Zang	Tibet	Plateau
		JX068210.1	Zang	Tibet	Plateau
		JX068215.1	Zang	Tibet	Plateau

		JX068219.1	Zang	Tibet	Plateau
		JX068258.1	Zang	Yunnan	Plateau
		JX068260.1	Zang	Yunnan	Plateau
		JX068309.1	Zang	Gansu	Plateau
		JX068310.1	Zang	Gansu	Plateau
		JX068311.1	Zang	Gansu	Plateau
		JX068312.1	Zang	Gansu	Plateau
		JX068313.1	Zang	Gansu	Plateau
		JX068360.1	Zang	Sichuan	Plateau
		JX068363.1	Zang	Sichuan	Plateau
		JX068379.1	Zang	Sichuan	Plateau
		JX068382.1	Zang	Sichuan	Plateau
		JX068393.1	Pengzhou Mountain	Sichuan	Southwest China
		JX068394.1	Pengzhou Mountain	Sichuan	Southwest China
		JX068403.1	Pengzhou Mountain	Sichuan	Southwest China
		JX068404.1	Pengzhou Mountain	Sichuan	Southwest China
		JX068408.1	Pengzhou Mountain	Sichuan	Southwest China
		JX068409.1	Pengzhou Mountain	Sichuan	Southwest China
		JX068411.1	Pengzhou Mountain	Sichuan	Southwest China
		JX068414.1	Pengzhou Mountain	Sichuan	Southwest China
		JX068419.1	Pengzhou Mountain	Sichuan	Southwest China
		JX068423.1	Pengzhou Mountain	Sichuan	Southwest China
		JX068425.1	Pengzhou Mountain	Sichuan	Southwest China
		JX068431.1	Pengzhou Mountain	Sichuan	Southwest China
		JX068433.1	Pengzhou Mountain	Sichuan	Southwest China
		JX068435.1	Pengzhou Mountain	Sichuan	Southwest China
		JX068445.1	Wujin	Sichuan	Southwest China
		JX068449.1	Wujin	Sichuan	Southwest China
		JX068466.1	Wujin	Sichuan	Southwest China
		JX068467.1	Wujin	Sichuan	Southwest China
		JX068470.1	Wujin	Sichuan	Southwest China
		JX068486.1	Yanan	Sichuan	Southwest China
		JX068487.1	Yanan	Sichuan	Southwest China
		JX068491.1	Yanan	Sichuan	Southwest China
		JX068492.1	Yanan	Sichuan	Southwest China
		JX068493.1	Yanan	Sichuan	Southwest China
		JX068495.1	Yanan	Sichuan	Southwest China
		JX068498.1	Yanan	Sichuan	Southwest China
		JX068500.1	Yanan	Sichuan	Southwest China
		JX068501.1	Yanan	Sichuan	Southwest China
		JX068505.1	Yanan	Sichuan	Southwest China
		JX068506.1	Yanan	Sichuan	Southwest China
		JX068509.1	Yanan	Sichuan	Southwest China
		JX068512.1	Yanan	Sichuan	Southwest China
		JX068514.1	Yanan	Sichuan	Southwest China
		JX068518.1	Yanan	Sichuan	Southwest China
		JX068519.1	Yanan	Sichuan	Southwest China
		JX068521.1	Yanan	Sichuan	Southwest China
		JX068522.1	Yanan	Sichuan	Southwest China
		KC505406.1	Neijiang	Sichuan	Southwest China
		KC505407.1	Pengzhou Mountain	Sichuan	Southwest China

		KF601700.1	Taoyuan Black	Hunan	Southeast China
		dGH2	ancient pig	Shanxi	North China
		dGH4	ancient pig	Shanxi	North China
		dJH1	ancient pig	Henan	North China
		dNZT4	ancient pig	Hebei	North China
		dQLQ5	ancient pig	Hubei	Southeast China
4	229	AF276924.1	Wannan Spotted	Anhui	Southeast China
		AF276926.1	Wannan Spotted	Anhui	Southeast China
		AF276930.1	Jinhua	Zhejiang	Southeast China
		AF276931.1	Putian	Fujian	Southeast China
		AF276932.1	Wannan Spotted	Anhui	Southeast China
		AM040641.1	Taoyuan	Taiwan	Southeast China
		AM040642.1	Taoyuan	Taiwan	Southeast China
		AM040643.1	Taoyuan	Taiwan	Southeast China
		AM040644.1	Taoyuan	Taiwan	Southeast China
		AM040645.1	Taoyuan	Taiwan	Southeast China
		AM040646.1	Taoyuan	Taiwan	Southeast China
		AM040647.1	Meishan	Taiwan	Southeast China
		AM040648.1	Meishan	Taiwan	Southeast China
		AM040653.1	Taoyuan	Taiwan	Southeast China
		AY178264.1	Wild boar	Yunnan	Southwest China
		AY178268.1	Wild boar	Yunnan	Southwest China
		AY486115.1	Ganzhongnan Spotted	Jiangxi	Southeast China
		DQ152876.2	Xiangxi Black	Hunan	Southeast China
		DQ152884.2	Shanggao	Jiangxi	Southeast China
		DQ152894.2	Xiangxi Black	Hunan	Southeast China
		DQ379146.2	Tongcheng	Hubei	Southeast China
		DQ379147.2	Tongcheng	Hubei	Southeast China
		DQ379148.2	Jinhua	Zhejiang	Southeast China
		DQ379149.2	Jinhua	Zhejiang	Southeast China
		DQ379150.2	Jiangquhai	Jiangsu	Southeast China
		DQ496252.1	Aba	Sichuan	Plateau
		DQ496255.1	Aba	Sichuan	Plateau
		DQ496281.1	Bihu	Zhejiang	Southeast China
		DQ496330.1	Chalu	Zhejiang	Southeast China
		DQ496332.1	Chalu	Zhejiang	Southeast China
		DQ496333.1	Chalu	Zhejiang	Southeast China
		DQ496374.1	Diannan small-ear	Yunnan	Southwest China
		DQ496383.1	Diannan small-ear	Yunnan	Southwest China
		DQ496386.1	Diannan small-ear	Yunnan	Southwest China
		DQ496389.1	Diannan small-ear	Yunnan	Southwest China
		DQ496390.1	Diannan small-ear	Yunnan	Southwest China
		DQ496394.1	Zang	Yunnan	Plateau
		DQ496395.1	Zang	Yunnan	Plateau
		DQ496396.1	Zang	Yunnan	Plateau
		DQ496397.1	Zang	Yunnan	Plateau
		DQ496398.1	Zang	Yunnan	Plateau
		DQ496408.1	Zang	Yunnan	Plateau
		DQ496412.1	Erhualian	Jiangsu	Southeast China
		DQ496442.1	Hanjiang Black	Shaanxi	North China
		DQ496449.1	Huai	Fujian	Southeast China

DQ496463.1	Huzhu	Qinghai	Plateau
DQ496478.1	Jianli	Hubei	Southeast China
DQ496489.1	Jinhua	Zhejiang	Southeast China
DQ496491.1	Jinhua	Zhejiang	Southeast China
DQ496495.1	Jiangquhai	Jiangsu	Southeast China
DQ496497.1	Jiangquhai	Jiangsu	Southeast China
DQ496551.1	Mingguang small-ear	Yunnan	Southwest China
DQ496552.1	Mingguang small-ear	Yunnan	Southwest China
DQ496567.1	Min	Northeast China	Northeast China
DQ496568.1	Min	Northeast China	Northeast China
DQ496574.1	Mingguang small-ear	Yunnan	Southwest China
DQ496575.1	Mingguang small-ear	Yunnan	Southwest China
DQ496579.1	Mingguang small-ear	Yunnan	Southwest China
DQ496630.1	Qingping	Hubei	Southeast China
DQ496631.1	Qingping	Hubei	Southeast China
DQ496632.1	Qingping	Hubei	Southeast China
DQ496644.1	Rongchang	Chongqing	Southwest China
DQ496645.1	Rongchang	Chongqing	Southwest China
DQ496649.1	Rongchang	Chongqing	Southwest China
DQ496657.1	Saba	Yunnan	Southwest China
DQ496658.1	Saba	Yunnan	Southwest China
DQ496669.1	Saba	Yunnan	Southwest China
DQ496709.1	Shengxian Spotted	Zhejiang	Southeast China
DQ496734.1	Wannan Spotted	Anhui	Southeast China
DQ496735.1	Wannan Spotted	Anhui	Southeast China
DQ496736.1	Wannan Spotted	Anhui	Southeast China
DQ496738.1	Wannan Spotted	Anhui	Southeast China
DQ496739.1	Wannan Spotted	Anhui	Southeast China
DQ496740.1	Wannan Spotted	Anhui	Southeast China
DQ496794.1	Wild boar	Hainan	Southeast China
DQ496798.1	Wild boar	Hainan	Southeast China
DQ496810.1	Wild boar	Hainan	Southeast China
DQ496814.1	Wild boar	Hainan	Southeast China
DQ496817.1	Wild boar	Jiangxi	Southeast China
DQ496853.1	Wild boar	Yunnan	Southwest China
DQ496861.1	Wild boar	Yunnan	Southwest China
DQ496864.1	Wild boar	Yunnan	Southwest China
DQ496868.1	Wild boar	Yunnan	Southwest China
DQ496873.1	Wild boar	Yunnan	Southwest China
DQ496888.1	Wild boar	Zhejiang	Southeast China
DQ496913.1	Wei	Anhui	Southeast China
DQ496914.1	Wei	Anhui	Southeast China
DQ496917.1	Wei	Anhui	Southeast China
DQ496918.1	Wei	Anhui	Southeast China
DQ496919.1	Wei	Anhui	Southeast China
DQ496925.1	Wei	Anhui	Southeast China
DQ496927.1	Wei	Anhui	Southeast China
DQ496965.1	Xiang	Guizhou	Southwest China
DQ534707.2	Taoyuan	Taiwan	Southeast China
EF545572.1	Wild boar	Yunnan	Southwest China
EF545577.1	Wei	Anhui	Southeast China

EF545578.1	Aba	Sichuan	Plateau
EF545581.1	Qingping	Hubei	Southeast China
EF545582.1	Qingping	Hubei	Southeast China
EF545590.1	Bihu	Zhejiang	Southeast China
EF590147.1	Wannan Spotted	Anhui	Southeast China
EF590148.1	Tunchang	Hainan	Southeast China
EF590151.1	Shanggao	Jiangxi	Southeast China
EF590163.1	Jianli	Hubei	Southeast China
EF590176.1	Daweizi	Hunan	Southeast China
EF590182.1	Qingping	Hubei	Southeast China
EF590193.1	Wei	Anhui	Southeast China
EF590195.1	Jiangquhai	Jiangsu	Southeast China
EF590199.1	Yanan	Sichuan	Southwest China
EU979171.1	Wulian Black	Shandong	North China
EU979175.1	Wulian Black	Shandong	North China
FJ601391.1	Nanyang Black	Henan	North China
FJ601392.1	Jianli	Hubei	Southeast China
FJ601398.1	Jiangquhai	Jiangsu	Southeast China
FJ601400.1	Shawutou	Jiangsu	Southeast China
FJ601407.1	Yanan	Sichuan	Southwest China
FJ601420.1	Banna small-ear	Yunnan	Southwest China
FJ601428.1	Huchuan Mountain	Hunan	Southeast China
FJ601429.1	Huchuan Mountain	Hunan	Southeast China
FJ601447.1	Lingao	Hainan	Southeast China
FJ601448.1	Jiangkoluobo	Guizhou	Southwest China
FJ601449.1	Jiangkoluobo	Guizhou	Southwest China
FJ601456.1	Wei	Anhui	Southeast China
FJ601457.1	Wei	Anhui	Southeast China
FJ601466.1	Yangxin	Hubei	Southeast China
FJ601481.1	Wuyi Black	Fujian	Southeast China
FJ601490.1	Ju'nan	Shandong	North China
FJ601501.1	Donglan	Guangxi	Southwest China
FJ601513.1	Ningxiang	Hunan	Southeast China
FJ601525.1	Wild boar	Jiangxi	Southeast China
GQ169775.1	Taoyuan	Taiwan	Southeast China
HM026672.1	Jiangquhai	Jiangsu	Southeast China
HM026679.1	Rongchang	Chongqing	Southwest China
HQ148352.1	Zang	Sichuan	Plateau
HQ148354.1	Zang	Sichuan	Plateau
HQ148367.1	Hezuo	Gansu	Plateau
HQ148368.1	Hezuo	Gansu	Plateau
HQ148403.1	Zang	Tibet	Plateau
HQ148410.1	Zang	Tibet	Plateau
HQ148418.1	Zang	Yunnan	Plateau
HQ148422.1	Zang	Yunnan	Plateau
JX068024.1	Jinhua	Zhejiang	Southeast China
JX068025.1	Jinhua	Zhejiang	Southeast China
JX068027.1	Jinhua	Zhejiang	Southeast China
JX068029.1	Jinhua	Zhejiang	Southeast China
JX068031.1	Jinhua	Zhejiang	Southeast China
JX068032.1	Jinhua	Zhejiang	Southeast China

		JX068033.1	Jinhua	Zhejiang	Southeast China
		JX068034.1	Jinhua	Zhejiang	Southeast China
		JX068043.1	Jinhua	Zhejiang	Southeast China
		JX068050.1	Jinhua	Zhejiang	Southeast China
		JX068055.1	Jinhua	Zhejiang	Southeast China
		JX068059.1	Jinhua	Zhejiang	Southeast China
		JX068074.1	Neijiang	Sichuan	Southwest China
		JX068086.1	Neijiang	Sichuan	Southwest China
		JX068095.1	Neijiang	Sichuan	Southwest China
		JX068117.1	Zang	Tibet	Plateau
		JX068118.1	Zang	Tibet	Plateau
		JX068156.1	Zang	Tibet	Plateau
		JX068203.1	Zang	Tibet	Plateau
		JX068205.1	Zang	Tibet	Plateau
		JX068218.1	Zang	Tibet	Plateau
		JX068226.1	Zang	Tibet	Plateau
		JX068229.1	Zang	Tibet	Plateau
		JX068232.1	Zang	Tibet	Plateau
		JX068233.1	Zang	Tibet	Plateau
		JX068234.1	Zang	Tibet	Plateau
		JX068237.1	Zang	Yunnan	Plateau
		JX068238.1	Zang	Yunnan	Plateau
		JX068239.1	Zang	Yunnan	Plateau
		JX068240.1	Zang	Yunnan	Plateau
		JX068243.1	Zang	Yunnan	Plateau
		JX068244.1	Zang	Yunnan	Plateau
		JX068245.1	Zang	Yunnan	Plateau
		JX068246.1	Zang	Yunnan	Plateau
		JX068248.1	Zang	Yunnan	Plateau
		JX068249.1	Zang	Yunnan	Plateau
		JX068252.1	Zang	Yunnan	Plateau
		JX068253.1	Zang	Yunnan	Plateau
		JX068254.1	Zang	Yunnan	Plateau
		JX068255.1	Zang	Yunnan	Plateau
		JX068256.1	Zang	Yunnan	Plateau
		JX068257.1	Zang	Yunnan	Plateau
		JX068259.1	Zang	Yunnan	Plateau
		JX068263.1	Zang	Yunnan	Plateau
		JX068264.1	Zang	Yunnan	Plateau
		JX068265.1	Zang	Yunnan	Plateau
		JX068266.1	Zang	Yunnan	Plateau
		JX068273.1	Zang	Yunnan	Plateau
		JX068274.1	Zang	Yunnan	Plateau
		JX068280.1	Zang	Sichuan	Plateau
		JX068281.1	Zang	Sichuan	Plateau
		JX068283.1	Zang	Sichuan	Plateau
		JX068285.1	Zang	Sichuan	Plateau
		JX068287.1	Zang	Sichuan	Plateau
		JX068288.1	Zang	Sichuan	Plateau
		JX068291.1	Zang	Sichuan	Plateau
		JX068292.1	Zang	Sichuan	Plateau

		JX068303.1	Zang	Sichuan	Plateau
		JX068304.1	Zang	Sichuan	Plateau
		JX068305.1	Zang	Sichuan	Plateau
		JX068306.1	Zang	Sichuan	Plateau
		JX068355.1	Zang	Sichuan	Plateau
		JX068358.1	Zang	Sichuan	Plateau
		JX068362.1	Zang	Sichuan	Plateau
		JX068365.1	Zang	Sichuan	Plateau
		JX068366.1	Zang	Sichuan	Plateau
		JX068368.1	Zang	Sichuan	Plateau
		JX068369.1	Zang	Sichuan	Plateau
		JX068370.1	Zang	Sichuan	Plateau
		JX068371.1	Zang	Sichuan	Plateau
		JX068372.1	Zang	Sichuan	Plateau
		JX068373.1	Zang	Sichuan	Plateau
		JX068375.1	Zang	Sichuan	Plateau
		JX068381.1	Zang	Sichuan	Plateau
		JX068383.1	Zang	Sichuan	Plateau
		JX068384.1	Zang	Sichuan	Plateau
		JX068488.1	Yanan	Sichuan	Southwest China
		JX068497.1	Yanan	Sichuan	Southwest China
		JX068507.1	Yanan	Sichuan	Southwest China
		JX068511.1	Yanan	Sichuan	Southwest China
		JX068524.1	Yanan	Sichuan	Southwest China
		KF472177.1	Shaziling	Hunan	Southeast China
		dCN1	ancient pig	Qinghai	Northwest China
		dCS2	ancient pig	Hebei	North China
		dJH2	ancient pig	Henan	North China
		dQLQ1	ancient pig	Hubei	Southeast China
		dQLQ2	ancient pig	Hubei	Southeast China
		dXLW1	ancient pig	Inner mongolia (East)	Northeast China
5	1	AF276929.1	Neijiang	Sichuan	Southwest China
6	2	AY178252.1	Wild boar	Yunnan	Southwest China
		DQ496264.1	Aba	Sichuan	Plateau
7	39	AY178254.1	Wild boar	Yunnan	Southwest China
		DQ152879.2	Bamei	Qinghai	Northwest China
		DQ379163.2	Bamei	Qinghai	Northwest China
		DQ379164.2	Bamei	Qinghai	Northwest China
		DQ496262.1	Aba	Sichuan	Plateau
		DQ496266.1	Aba	Sichuan	Plateau
		DQ496267.1	Aba	Sichuan	Plateau
		DQ496445.1	Hanjiang Black	Shaanxi	North China
		DQ496446.1	Hanjiang Black	Shaanxi	North China
		DQ496455.1	Huzhu	Qinghai	Plateau
		DQ496456.1	Huzhu	Qinghai	Plateau
		DQ496464.1	Huzhu	Qinghai	Plateau
		DQ496465.1	Huzhu	Qinghai	Plateau
		FJ601476.1	Bamei	Qinghai	Northwest China
		FJ601477.1	Bamei	Qinghai	Northwest China
		HQ148344.1	Zang	Sichuan	Plateau
		HQ148359.1	Zang	Tibet	Plateau

		JX068142.1	Zang	Tibet	Plateau
		JX068143.1	Zang	Tibet	Plateau
		JX068183.1	Zang	Tibet	Plateau
		JX068184.1	Zang	Tibet	Plateau
		JX068185.1	Zang	Tibet	Plateau
		JX068186.1	Zang	Tibet	Plateau
		JX068327.1	Zang	Gansu	Plateau
		JX068328.1	Zang	Gansu	Plateau
		JX068330.1	Zang	Gansu	Plateau
		JX068335.1	Zang	Gansu	Plateau
		JX068336.1	Zang	Gansu	Plateau
		JX068337.1	Zang	Gansu	Plateau
		JX068338.1	Zang	Gansu	Plateau
		JX068339.1	Zang	Gansu	Plateau
		JX068341.1	Zang	Gansu	Plateau
		JX068356.1	Zang	Sichuan	Plateau
		JX068385.1	Zang	Sichuan	Plateau
		JX068387.1	Zang	Sichuan	Plateau
		JX068388.1	Zang	Sichuan	Plateau
		JX068389.1	Zang	Sichuan	Plateau
		JX068390.1	Zang	Sichuan	Plateau
		KC493607.1	Zang	Sichuan	Plateau
8	11	AY178255.1	Wild boar	Yunnan	Southwest China
		DQ496655.1	Saba	Yunnan	Southwest China
		DQ496656.1	Saba	Yunnan	Southwest China
		DQ496660.1	Saba	Yunnan	Southwest China
		DQ496676.1	Saba	Yunnan	Southwest China
		DQ496677.1	Saba	Yunnan	Southwest China
		DQ496679.1	Saba	Yunnan	Southwest China
		DQ496680.1	Saba	Yunnan	Southwest China
		EF545574.1	Saba	Yunnan	Southwest China
		HQ148450.1	Saba	Yunnan	Southwest China
		HQ148451.1	Saba	Yunnan	Southwest China
9	46	AY178256.1	Wild boar	Yunnan	Southwest China
		AY178258.1	Wild boar	Yunnan	Southwest China
		AY178266.1	Wild boar	Yunnan	Southwest China
		AY178267.1	Wild boar	Yunnan	Southwest China
		AY178270.1	Wild boar	Yunnan	Southwest China
		DQ496269.1	Aba	Sichuan	Plateau
		DQ496305.1	Diannan small-ear	Yunnan	Southwest China
		DQ496308.1	Diannan small-ear	Yunnan	Southwest China
		DQ496309.1	Diannan small-ear	Yunnan	Southwest China
		DQ496311.1	Baoshan	Yunnan	Southwest China
		DQ496312.1	Baoshan	Yunnan	Southwest China
		DQ496326.1	Baoshan	Yunnan	Southwest China
		DQ496365.1	Diannan small-ear	Yunnan	Southwest China
		DQ496368.1	Diannan small-ear	Yunnan	Southwest China
		DQ496371.1	Diannan small-ear	Yunnan	Southwest China
		DQ496372.1	Diannan small-ear	Yunnan	Southwest China
		DQ496373.1	Diannan small-ear	Yunnan	Southwest China
		DQ496375.1	Diannan small-ear	Yunnan	Southwest China

		DQ496376.1	Diannan small-ear	Yunnan	Southwest China
		DQ496384.1	Diannan small-ear	Yunnan	Southwest China
		DQ496385.1	Diannan small-ear	Yunnan	Southwest China
		DQ496388.1	Diannan small-ear	Yunnan	Southwest China
		DQ496391.1	Diannan small-ear	Yunnan	Southwest China
		DQ496423.1	Fugong	Yunnan	Southwest China
		DQ496674.1	Saba	Yunnan	Southwest China
		DQ496675.1	Saba	Yunnan	Southwest China
		DQ496741.1	Wild boar	Yunnan	Southwest China
		DQ496869.1	Wild boar	Yunnan	Southwest China
		DQ496870.1	Wild boar	Yunnan	Southwest China
		DQ496882.1	Wild boar	Yunnan	Southwest China
		DQ496898.1	Wild boar	Zhejiang	Southeast China
		DQ496972.1	Yahe	Sichuan	Southwest China
		DQ779428.1	Wild boar	Taiwan	Southeast China
		EF590196.1	Qiandong Spotted	Guizhou	Southwest China
		FJ601424.1	Baoshan	Yunnan	Southwest China
		FJ601425.1	Baoshan	Yunnan	Southwest China
		GQ220328.1	Banna Mini	Yunnan	Southwest China
		HQ148363.1	Zang	Tibet	Plateau
		HQ148415.1	Zang	Yunnan	Plateau
		HQ148442.1	Zang	Yunnan	Plateau
		HQ148447.1	Zang	Yunnan	Plateau
		HQ148494.1	Diannan small-ear	Yunnan	Southwest China
		HQ148495.1	Diannan small-ear	Yunnan	Southwest China
		HQ148506.1	Diannan small-ear	Yunnan	Southwest China
		HQ148508.1	Diannan small-ear	Yunnan	Southwest China
		HQ148548.1	Baoshan	Yunnan	Southwest China
10	438	AY178257.1	Wild boar	Yunnan	Southwest China
		AY178259.1	Wild boar	Yunnan	Southwest China
		AY178260.1	Wild boar	Yunnan	Southwest China
		AY178262.1	Wild boar	Yunnan	Southwest China
		AY178263.1	Wild boar	Yunnan	Southwest China
		AY178272.1	Wild boar	Yunnan	Southwest China
		AY178274.1	Wild boar	Yunnan	Southwest China
		AY884683.1	Wild boar	Hunan	Southeast China
		DQ152869.2	Xiangxi Black	Hunan	Southeast China
		DQ152881.2	Qianbei Black	Guizhou	Southwest China
		DQ152882.2	Qianbei Black	Guizhou	Southwest China
		DQ152895.2	Xiangxi Black	Hunan	Southeast China
		DQ379103.2	Bamei	Qinghai	Northwest China
		DQ379104.2	Bamei	Qinghai	Northwest China
		DQ379174.2	Qianbei Black	Guizhou	Southwest China
		DQ379181.2	Longlin	Guangxi	Southwest China
		DQ379182.2	Longlin	Guangxi	Southwest China
		DQ379183.2	Longlin	Guangxi	Southwest China
		DQ379184.2	Longlin	Guangxi	Southwest China
		DQ379185.2	Longlin	Guangxi	Southwest China
		DQ379186.2	Shanggao	Jiangxi	Southeast China
		DQ496253.1	Aba	Sichuan	Plateau
		DQ496254.1	Aba	Sichuan	Plateau

DQ496256.1	Aba	Sichuan	Plateau
DQ496265.1	Aba	Sichuan	Plateau
DQ496270.1	Aba	Sichuan	Plateau
DQ496271.1	Aba	Sichuan	Plateau
DQ496272.1	Aba	Sichuan	Plateau
DQ496287.1	Bamei	Qinghai	Northwest China
DQ496290.1	Bamei	Qinghai	Northwest China
DQ496292.1	Bamei	Qinghai	Northwest China
DQ496293.1	Bamaxiang	Guangxi	Southwest China
DQ496294.1	Bamaxiang	Guangxi	Southwest China
DQ496295.1	Bamaxiang	Guangxi	Southwest China
DQ496296.1	Bamaxiang	Guangxi	Southwest China
DQ496297.1	Bamaxiang	Guangxi	Southwest China
DQ496298.1	Bamaxiang	Guangxi	Southwest China
DQ496299.1	Bamaxiang	Guangxi	Southwest China
DQ496300.1	Bamaxiang	Guangxi	Southwest China
DQ496301.1	Bamaxiang	Guangxi	Southwest China
DQ496302.1	Bamaxiang	Guangxi	Southwest China
DQ496307.1	Diannan small-ear	Yunnan	Southwest China
DQ496310.1	Baoshan	Yunnan	Southwest China
DQ496313.1	Baoshan	Yunnan	Southwest China
DQ496316.1	Baoshan	Yunnan	Southwest China
DQ496317.1	Baoshan	Yunnan	Southwest China
DQ496318.1	Baoshan	Yunnan	Southwest China
DQ496319.1	Baoshan	Yunnan	Southwest China
DQ496320.1	Baoshan	Yunnan	Southwest China
DQ496321.1	Baoshan	Yunnan	Southwest China
DQ496324.1	Baoshan	Yunnan	Southwest China
DQ496325.1	Baoshan	Yunnan	Southwest China
DQ496327.1	Baoshan	Yunnan	Southwest China
DQ496331.1	Chalu	Zhejiang	Southeast China
DQ496335.1	Chenghua	Sichuan	Southwest China
DQ496336.1	Chenghua	Sichuan	Southwest China
DQ496337.1	Chenghua	Sichuan	Southwest China
DQ496338.1	Chenghua	Sichuan	Southwest China
DQ496358.1	Dahe	Yunnan	Southwest China
DQ496359.1	Dahe	Yunnan	Southwest China
DQ496360.1	Dahe	Yunnan	Southwest China
DQ496366.1	Diannan small-ear	Yunnan	Southwest China
DQ496367.1	Diannan small-ear	Yunnan	Southwest China
DQ496369.1	Diannan small-ear	Yunnan	Southwest China
DQ496370.1	Diannan small-ear	Yunnan	Southwest China
DQ496378.1	Diannan small-ear	Yunnan	Southwest China
DQ496379.1	Diannan small-ear	Yunnan	Southwest China
DQ496380.1	Diannan small-ear	Yunnan	Southwest China
DQ496381.1	Diannan small-ear	Yunnan	Southwest China
DQ496382.1	Diannan small-ear	Yunnan	Southwest China
DQ496387.1	Diannan small-ear	Yunnan	Southwest China
DQ496393.1	Zang	Yunnan	Plateau
DQ496402.1	Zang	Yunnan	Plateau
DQ496403.1	Zang	Yunnan	Plateau

		DQ496405.1	Zang	Yunnan	Plateau
		DQ496409.1	Zang	Yunnan	Plateau
		DQ496424.1	Zang	Sichuan	Plateau
		DQ496425.1	Zang	Sichuan	Plateau
		DQ496426.1	Zang	Sichuan	Plateau
		DQ496427.1	Zang	Sichuan	Plateau
		DQ496429.1	Zang	Sichuan	Plateau
		DQ496430.1	Zang	Sichuan	Plateau
		DQ496431.1	Zang	Sichuan	Plateau
		DQ496432.1	Zang	Sichuan	Plateau
		DQ496434.1	Zang	Sichuan	Plateau
		DQ496435.1	Zang	Sichuan	Plateau
		DQ496436.1	Gaopo	Guizhou	Southwest China
		DQ496438.1	Gaopo	Guizhou	Southwest China
		DQ496439.1	Hanjiang Black	Shaanxi	North China
		DQ496441.1	Hanjiang Black	Shaanxi	North China
		DQ496444.1	Hanjiang Black	Shaanxi	North China
		DQ496453.1	Huzhu	Qinghai	Plateau
		DQ496483.1	Jiaxing Black	Zhejiang	Southeast China
		DQ496490.1	Jinhua	Zhejiang	Southeast China
		DQ496537.1	Leping Spotted	Jiangxi	Southeast China
		DQ496548.1	Mingguang small-ear	Yunnan	Southwest China
		DQ496549.1	Mingguang small-ear	Yunnan	Southwest China
		DQ496550.1	Mingguang small-ear	Yunnan	Southwest China
		DQ496553.1	Mingguang small-ear	Yunnan	Southwest China
		DQ496555.1	Min	Northeast China	Northeast China
		DQ496556.1	Min	Northeast China	Northeast China
		DQ496561.1	Min	Northeast China	Northeast China
		DQ496564.1	Min	Northeast China	Northeast China
		DQ496566.1	Min	Northeast China	Northeast China
		DQ496569.1	Min	Northeast China	Northeast China
		DQ496572.1	Mingguang small-ear	Yunnan	Southwest China
		DQ496573.1	Mingguang small-ear	Yunnan	Southwest China
		DQ496580.1	Mingguang small-ear	Yunnan	Southwest China
		DQ496606.1	Qianbei Black	Guizhou	Southwest China
		DQ496607.1	Qianbei Black	Guizhou	Southwest China
		DQ496608.1	Qianbei Black	Guizhou	Southwest China
		DQ496610.1	Qianbei Black	Guizhou	Southwest China
		DQ496611.1	Qianbei Black	Guizhou	Southwest China
		DQ496612.1	Qianbei Black	Guizhou	Southwest China
		DQ496613.1	Qianbei Black	Guizhou	Southwest China
		DQ496614.1	Qianbei Black	Guizhou	Southwest China
		DQ496615.1	Qianbei Black	Guizhou	Southwest China
		DQ496616.1	Qianbei Black	Guizhou	Southwest China
		DQ496618.1	Qianbei Black	Guizhou	Southwest China
		DQ496628.1	Qingping	Hubei	Southeast China
		DQ496633.1	Qingping	Hubei	Southeast China
		DQ496636.1	Qingping	Hubei	Southeast China
		DQ496639.1	Xiang	Guizhou	Southwest China
		DQ496640.1	Xiang	Guizhou	Southwest China
		DQ496641.1	Xiang	Guizhou	Southwest China

DQ496642.1	Xiang	Guizhou	Southwest China
DQ496651.1	Rongchang	Chongqing	Southwest China
DQ496661.1	Saba	Yunnan	Southwest China
DQ496662.1	Saba	Yunnan	Southwest China
DQ496663.1	Saba	Yunnan	Southwest China
DQ496664.1	Saba	Yunnan	Southwest China
DQ496665.1	Saba	Yunnan	Southwest China
DQ496666.1	Saba	Yunnan	Southwest China
DQ496667.1	Saba	Yunnan	Southwest China
DQ496670.1	Saba	Yunnan	Southwest China
DQ496671.1	Saba	Yunnan	Southwest China
DQ496672.1	Saba	Yunnan	Southwest China
DQ496673.1	Saba	Yunnan	Southwest China
DQ496678.1	Saba	Yunnan	Southwest China
DQ496681.1	Saba	Yunnan	Southwest China
DQ496682.1	Saba	Yunnan	Southwest China
DQ496683.1	Saba	Yunnan	Southwest China
DQ496684.1	Saba	Yunnan	Southwest China
DQ496685.1	Saba	Yunnan	Southwest China
DQ496686.1	Saba	Yunnan	Southwest China
DQ496687.1	Saba	Yunnan	Southwest China
DQ496688.1	Saba	Yunnan	Southwest China
DQ496689.1	Saba	Yunnan	Southwest China
DQ496690.1	Saba	Yunnan	Southwest China
DQ496691.1	Saba	Yunnan	Southwest China
DQ496692.1	Saba	Yunnan	Southwest China
DQ496695.1	Meishan	Jiangxi	Southeast China
DQ496701.1	Meishan	Jiangxi	Southeast China
DQ496761.1	Wild boar	Northeast China	Northeast China
DQ496762.1	Wild boar	Northeast China	Northeast China
DQ496767.1	Wild boar	Northeast China	Northeast China
DQ496801.1	Wild boar	Hainan	Southeast China
DQ496818.1	Wild boar	Jiangxi	Southeast China
DQ496830.1	Wild boar	Guizhou	Southwest China
DQ496832.1	Wild boar	Sichuan	Southwest China
DQ496846.1	Wild boar	Sichuan	Southwest China
DQ496850.1	Wild boar	Yunnan	Southwest China
DQ496854.1	Wild boar	Yunnan	Southwest China
DQ496855.1	Wild boar	Yunnan	Southwest China
DQ496856.1	Wild boar	Yunnan	Southwest China
DQ496857.1	Wild boar	Yunnan	Southwest China
DQ496859.1	Wild boar	Yunnan	Southwest China
DQ496863.1	Wild boar	Yunnan	Southwest China
DQ496867.1	Wild boar	Yunnan	Southwest China
DQ496874.1	Wild boar	Yunnan	Southwest China
DQ496878.1	Wild boar	Yunnan	Southwest China
DQ496879.1	Wild boar	Yunnan	Southwest China
DQ496880.1	Wild boar	Yunnan	Southwest China
DQ496897.1	Wild boar	Zhejiang	Southeast China
DQ496899.1	Wild boar	Zhejiang	Southeast China
DQ496970.1	Yahe	Sichuan	Southwest China

DQ496973.1	Yahe	Sichuan	Southwest China
DQ496975.1	Yahe	Sichuan	Southwest China
EF536857.1	Kele	Guizhou	Southwest China
EF545567.1	Saba	Yunnan	Southwest China
EF545573.1	Wild boar	Yunnan	Southwest China
EF545575.1	Meishan	Jiangxi	Southeast China
EF545576.1	Zang	Yunnan	Plateau
EF545579.1	Wild boar	Jiangxi	Southeast China
EF545587.1	Huzhu	Qinghai	Plateau
EF545589.1	Yimeng Black	Shandong	North China
EF590143.1	Huai	Fujian	Southeast China
EF590145.1	Yangxin	Hubei	Southeast China
EF590150.1	Taoyuan	Taiwan	Southeast China
EF590152.1	Shaziling	Hunan	Southeast China
EF590154.1	Qianbei Black	Guizhou	Southwest China
EF590172.1	Xiang	Guizhou	Southwest China
EF590173.1	Kele	Guizhou	Southwest China
EF590175.1	Chenghua	Sichuan	Southwest China
EF590177.1	Banna mini	Yunnan	Southwest China
EF590178.1	Bamaxiang	Guangxi	Southwest China
EF590187.1	Hanjiang Black	Shaanxi	North China
EF590192.1	Jiaxing Black	Zhejiang	Southeast China
EF590194.1	Exi Black	Hubei	Southeast China
EU979178.1	Wulian Black	Shandong	North China
EU979179.1	Wulian Black	Shandong	North China
FJ601402.1	Banna mini	Yunnan	Southwest China
FJ601403.1	Banna mini	Yunnan	Southwest China
FJ601410.1	Xingzi Black	Jiangxi	Southeast China
FJ601415.1	Dahe	Yunnan	Southwest China
FJ601421.1	Banna small-ear	Yunnan	Southwest China
FJ601422.1	Saba	Yunnan	Southwest China
FJ601423.1	Saba	Yunnan	Southwest China
FJ601431.1	Rongchang	Chongqing	Southwest China
FJ601432.1	Wenchang	Hainan	Southeast China
FJ601437.1	Hanjiang Black	Shaanxi	North China
FJ601438.1	Bamaxiang	Guangxi	Southwest China
FJ601439.1	Bamaxiang	Guangxi	Southwest China
FJ601442.1	Guanling	Guizhou	Southwest China
FJ601443.1	Guanling	Guizhou	Southwest China
FJ601452.1	Qianbei Black	Guizhou	Southwest China
FJ601453.1	Qianbei Black	Guizhou	Southwest China
FJ601470.1	Shanggao	Jiangxi	Southeast China
FJ601471.1	Shanggao	Jiangxi	Southeast China
FJ601487.1	Wuzhishan	Hainan	Southeast China
FJ601491.1	Taoyuan	Taiwan	Southeast China
FJ601495.1	Chenghua	Sichuan	Southwest China
FJ601498.1	Longlin	Guangxi	Southwest China
FJ601499.1	Xiang	Guizhou	Southwest China
FJ601500.1	Xiang	Guizhou	Southwest China
FJ601503.1	Daweizi	Hunan	Southeast China
FJ601508.1	Donglan	Guangxi	Southwest China

		FJ601512.1	Guizhong Spotted	Guizhou	Southwest China
		FJ601515.1	Exi Black	Hubei	Southeast China
		FJ601517.1	Bamaxiang	Guangxi	Southwest China
		FJ601519.1	Zang	Tibet	Plateau
		FJ601522.1	Wild boar	Zhejiang	Southeast China
		FJ601528.1	Wild boar	Heilongjiang	Northeast China
		FJ601529.1	Wild boar	Heilongjiang	Northeast China
		HM026673.1	Leping Spotted	Jiangxi	Southeast China
		HM026678.1	Qianbei Black	Guizhou	Southwest China
		HQ148311.1	Zang	Tibet	Plateau
		HQ148312.1	Zang	Tibet	Plateau
		HQ148314.1	Zang	Tibet	Plateau
		HQ148339.1	Zang	Tibet	Plateau
		HQ148396.1	Zang	Tibet	Plateau
		HQ148400.1	Zang	Tibet	Plateau
		HQ148404.1	Zang	Tibet	Plateau
		HQ148405.1	Zang	Tibet	Plateau
		HQ148414.1	Zang	Yunnan	Plateau
		HQ148479.1	Saba	Yunnan	Southwest China
		HQ148482.1	Saba	Yunnan	Southwest China
		HQ148485.1	Saba	Yunnan	Southwest China
		HQ148537.1	Baoshan	Yunnan	Southwest China
		HQ148540.1	Baoshan	Yunnan	Southwest China
		HQ148551.1	Baoshan	Yunnan	Southwest China
		JX068016.1	Chenghua	Sichuan	Southwest China
		JX068017.1	Chenghua	Sichuan	Southwest China
		JX068107.1	Neijiang	Sichuan	Southwest China
		JX068111.1	Neijiang	Sichuan	Southwest China
		JX068119.1	Zang	Tibet	Plateau
		JX068120.1	Zang	Tibet	Plateau
		JX068121.1	Zang	Tibet	Plateau
		JX068122.1	Zang	Tibet	Plateau
		JX068123.1	Zang	Tibet	Plateau
		JX068124.1	Zang	Tibet	Plateau
		JX068125.1	Zang	Tibet	Plateau
		JX068126.1	Zang	Tibet	Plateau
		JX068127.1	Zang	Tibet	Plateau
		JX068128.1	Zang	Tibet	Plateau
		JX068129.1	Zang	Tibet	Plateau
		JX068130.1	Zang	Tibet	Plateau
		JX068131.1	Zang	Tibet	Plateau
		JX068132.1	Zang	Tibet	Plateau
		JX068133.1	Zang	Tibet	Plateau
		JX068135.1	Zang	Tibet	Plateau
		JX068136.1	Zang	Tibet	Plateau
		JX068138.1	Zang	Tibet	Plateau
		JX068141.1	Zang	Tibet	Plateau
		JX068144.1	Zang	Tibet	Plateau
		JX068145.1	Zang	Tibet	Plateau
		JX068146.1	Zang	Tibet	Plateau
		JX068147.1	Zang	Tibet	Plateau

		JX068148.1	Zang	Tibet	Plateau
		JX068149.1	Zang	Tibet	Plateau
		JX068150.1	Zang	Tibet	Plateau
		JX068151.1	Zang	Tibet	Plateau
		JX068152.1	Zang	Tibet	Plateau
		JX068154.1	Zang	Tibet	Plateau
		JX068155.1	Zang	Tibet	Plateau
		JX068157.1	Zang	Tibet	Plateau
		JX068158.1	Zang	Tibet	Plateau
		JX068159.1	Zang	Tibet	Plateau
		JX068160.1	Zang	Tibet	Plateau
		JX068161.1	Zang	Tibet	Plateau
		JX068162.1	Zang	Tibet	Plateau
		JX068163.1	Zang	Tibet	Plateau
		JX068164.1	Zang	Tibet	Plateau
		JX068165.1	Zang	Tibet	Plateau
		JX068166.1	Zang	Tibet	Plateau
		JX068167.1	Zang	Tibet	Plateau
		JX068168.1	Zang	Tibet	Plateau
		JX068169.1	Zang	Tibet	Plateau
		JX068170.1	Zang	Tibet	Plateau
		JX068171.1	Zang	Tibet	Plateau
		JX068172.1	Zang	Tibet	Plateau
		JX068173.1	Zang	Tibet	Plateau
		JX068174.1	Zang	Tibet	Plateau
		JX068175.1	Zang	Tibet	Plateau
		JX068176.1	Zang	Tibet	Plateau
		JX068177.1	Zang	Tibet	Plateau
		JX068178.1	Zang	Tibet	Plateau
		JX068179.1	Zang	Tibet	Plateau
		JX068180.1	Zang	Tibet	Plateau
		JX068181.1	Zang	Tibet	Plateau
		JX068182.1	Zang	Tibet	Plateau
		JX068187.1	Zang	Tibet	Plateau
		JX068188.1	Zang	Tibet	Plateau
		JX068189.1	Zang	Tibet	Plateau
		JX068190.1	Zang	Tibet	Plateau
		JX068191.1	Zang	Tibet	Plateau
		JX068192.1	Zang	Tibet	Plateau
		JX068194.1	Zang	Tibet	Plateau
		JX068195.1	Zang	Tibet	Plateau
		JX068197.1	Zang	Tibet	Plateau
		JX068198.1	Zang	Tibet	Plateau
		JX068199.1	Zang	Tibet	Plateau
		JX068201.1	Zang	Tibet	Plateau
		JX068204.1	Zang	Tibet	Plateau
		JX068207.1	Zang	Tibet	Plateau
		JX068209.1	Zang	Tibet	Plateau
		JX068211.1	Zang	Tibet	Plateau
		JX068212.1	Zang	Tibet	Plateau
		JX068213.1	Zang	Tibet	Plateau

		JX068214.1	Zang	Tibet	Plateau
		JX068216.1	Zang	Tibet	Plateau
		JX068217.1	Zang	Tibet	Plateau
		JX068220.1	Zang	Tibet	Plateau
		JX068221.1	Zang	Tibet	Plateau
		JX068222.1	Zang	Tibet	Plateau
		JX068223.1	Zang	Tibet	Plateau
		JX068224.1	Zang	Tibet	Plateau
		JX068225.1	Zang	Tibet	Plateau
		JX068227.1	Zang	Tibet	Plateau
		JX068230.1	Zang	Tibet	Plateau
		JX068231.1	Zang	Tibet	Plateau
		JX068235.1	Zang	Tibet	Plateau
		JX068236.1	Zang	Tibet	Plateau
		JX068241.1	Zang	Yunnan	Plateau
		JX068242.1	Zang	Yunnan	Plateau
		JX068247.1	Zang	Yunnan	Plateau
		JX068250.1	Zang	Yunnan	Plateau
		JX068267.1	Zang	Yunnan	Plateau
		JX068268.1	Zang	Yunnan	Plateau
		JX068269.1	Zang	Yunnan	Plateau
		JX068270.1	Zang	Yunnan	Plateau
		JX068271.1	Zang	Yunnan	Plateau
		JX068275.1	Zang	Sichuan	Plateau
		JX068276.1	Zang	Sichuan	Plateau
		JX068277.1	Zang	Sichuan	Plateau
		JX068278.1	Zang	Sichuan	Plateau
		JX068279.1	Zang	Sichuan	Plateau
		JX068282.1	Zang	Sichuan	Plateau
		JX068284.1	Zang	Sichuan	Plateau
		JX068286.1	Zang	Sichuan	Plateau
		JX068289.1	Zang	Sichuan	Plateau
		JX068290.1	Zang	Sichuan	Plateau
		JX068293.1	Zang	Sichuan	Plateau
		JX068294.1	Zang	Sichuan	Plateau
		JX068295.1	Zang	Sichuan	Plateau
		JX068296.1	Zang	Sichuan	Plateau
		JX068297.1	Zang	Sichuan	Plateau
		JX068298.1	Zang	Sichuan	Plateau
		JX068299.1	Zang	Sichuan	Plateau
		JX068300.1	Zang	Sichuan	Plateau
		JX068301.1	Zang	Sichuan	Plateau
		JX068302.1	Zang	Sichuan	Plateau
		JX068322.1	Zang	Gansu	Plateau
		JX068374.1	Zang	Sichuan	Plateau
		JX068376.1	Zang	Sichuan	Plateau
		JX068377.1	Zang	Sichuan	Plateau
		JX068401.1	Pengzhou Mountain	Sichuan	Southwest China
		JX068402.1	Pengzhou Mountain	Sichuan	Southwest China
		JX068407.1	Pengzhou Mountain	Sichuan	Southwest China
		JX068410.1	Pengzhou Mountain	Sichuan	Southwest China

		JX068412.1	Pengzhou Mountain	Sichuan	Southwest China
		JX068413.1	Pengzhou Mountain	Sichuan	Southwest China
		JX068415.1	Pengzhou Mountain	Sichuan	Southwest China
		JX068416.1	Pengzhou Mountain	Sichuan	Southwest China
		JX068421.1	Pengzhou Mountain	Sichuan	Southwest China
		JX068426.1	Pengzhou Mountain	Sichuan	Southwest China
		JX068430.1	Pengzhou Mountain	Sichuan	Southwest China
		JX068432.1	Pengzhou Mountain	Sichuan	Southwest China
		JX068438.1	Pengzhou Mountain	Sichuan	Southwest China
		JX068447.1	Wujin	Sichuan	Southwest China
		JX068450.1	Wujin	Sichuan	Southwest China
		JX068452.1	Wujin	Sichuan	Southwest China
		JX068454.1	Wujin	Sichuan	Southwest China
		JX068455.1	Wujin	Sichuan	Southwest China
		JX068457.1	Wujin	Sichuan	Southwest China
		JX068458.1	Wujin	Sichuan	Southwest China
		JX068459.1	Wujin	Sichuan	Southwest China
		JX068460.1	Wujin	Sichuan	Southwest China
		JX068462.1	Wujin	Sichuan	Southwest China
		JX068463.1	Wujin	Sichuan	Southwest China
		JX068464.1	Wujin	Sichuan	Southwest China
		JX068465.1	Wujin	Sichuan	Southwest China
		JX068468.1	Wujin	Sichuan	Southwest China
		JX068469.1	Wujin	Sichuan	Southwest China
		JX068473.1	Wujin	Sichuan	Southwest China
		JX068474.1	Wujin	Sichuan	Southwest China
		JX068477.1	Wujin	Sichuan	Southwest China
		JX068478.1	Wujin	Sichuan	Southwest China
		JX068479.1	Wujin	Sichuan	Southwest China
		JX068480.1	Wujin	Sichuan	Southwest China
		JX068481.1	Wujin	Sichuan	Southwest China
		JX068489.1	Yanan	Sichuan	Southwest China
		JX068490.1	Yanan	Sichuan	Southwest China
		JX068494.1	Yanan	Sichuan	Southwest China
		JX068496.1	Yanan	Sichuan	Southwest China
		JX068499.1	Yanan	Sichuan	Southwest China
		JX068502.1	Yanan	Sichuan	Southwest China
		JX068503.1	Yanan	Sichuan	Southwest China
		JX068510.1	Yanan	Sichuan	Southwest China
		JX068513.1	Yanan	Sichuan	Southwest China
		JX068516.1	Yanan	Sichuan	Southwest China
		JX068520.1	Yanan	Sichuan	Southwest China
		JX068523.1	Yanan	Sichuan	Southwest China
		KC493610.1	Zang	Sichuan	Plateau
		KC493611.1	Zang	Sichuan	Plateau
		KC493612.1	Zang	Sichuan	Plateau
		KC505409.1	Yanan	Sichuan	Southwest China
		dDSQ1	ancient pig	Inner mongolia (East)	Northeast China
		dGH1	ancient pig	Shanxi	North China
		dGH3	ancient pig	Shanxi	North China
		dGH5	ancient pig	Shanxi	North China

		dGH6	ancient pig	Shanxi	North China
		dLJ1	ancient pig	Qinghai	Northwest China
		dLJ2	ancient pig	Qinghai	Northwest China
		dLJ3	ancient pig	Qinghai	Northwest China
		dTS1	ancient pig	Shanxi	North China
		dTS2	ancient pig	Shanxi	North China
		dTS4	ancient pig	Shanxi	North China
11	2	AY178269.1	Wild boar	Yunnan	Southwest China
		HQ148512.1	Diannan small-ear	Yunnan	Southwest China
12	1	AY230825.1	Erhualian	Jiangsu	Southeast China
13	1	AY486118.1	Xiang	Guizhou	Southwest China
14	4	AY884610.1	Wild boar	Sichuan	Southwest China
		JX068443.1	Wild boar	Chongqing	Southwest China
		JX068444.1	Wild boar	Chongqing	Southwest China
		KC505411.1	Wild boar	Chongqing	Southwest China
15	20	AY884627.1	Wild boar	Shanxi	North China
		AY884639.1	Wild boar	Shanxi	North China
		AY884640.1	Wild boar	Sichuan	Southwest China
		AY884684.1	Wild boar	Shanxi	North China
		DQ496788.1	Wild boar	Gansu	Northwest China
		DQ496789.1	Wild boar	Gansu	Northwest China
		DQ496790.1	Wild boar	Gansu	Northwest China
		DQ496833.1	Wild boar	Shaanxi	North China
		DQ496834.1	Wild boar	Shaanxi	North China
		DQ496837.1	Wild boar	Shaanxi	North China
		DQ496838.1	Wild boar	Shaanxi	North China
		DQ496839.1	Wild boar	Shaanxi	North China
		DQ496840.1	Wild boar	Shaanxi	North China
		DQ496841.1	Wild boar	Shaanxi	North China
		DQ496842.1	Wild boar	Shaanxi	North China
		DQ496844.1	Wild boar	Sichuan	Southwest China
		DQ496845.1	Wild boar	Sichuan	Southwest China
		JX068440.1	Wild boar	Chongqing	Southwest China
		JX068441.1	Wild boar	Chongqing	Southwest China
		JX068442.1	Wild boar	Chongqing	Southwest China
16	1	AY884641.1	Wild boar	Hunan	Southeast China
17	3	AY884685.1	Wild boar	Sichuan	Southwest China
		DQ496827.1	Wild boar	Guizhou	Southwest China
		DQ496831.1	Wild boar	Guizhou	Southwest China
18	43	DQ152875.2	Tongcheng	Hubei	Southeast China
		DQ152885.2	Shanggao	Jiangxi	Southeast China
		DQ152889.2	Jiangquhai	Jiangsu	Southeast China
		DQ379178.2	Shanggao	Jiangxi	Southeast China
		DQ496339.1	Dahuabai	Guangdong	Southeast China
		DQ496340.1	Dahuabai	Guangdong	Southeast China
		DQ496341.1	Dahuabai	Guangdong	Southeast China
		DQ496342.1	Dahuabai	Guangdong	Southeast China
		DQ496357.1	Dahuabai	Guangdong	Southeast China
		DQ496447.1	Huai	Fujian	Southeast China
		DQ496448.1	Huai	Fujian	Southeast China
		DQ496450.1	Huai	Fujian	Southeast China

		DQ496512.1	Lantang	Guangdong	Southeast China
		DQ496513.1	Lantang	Guangdong	Southeast China
		DQ496516.1	Lantang	Guangdong	Southeast China
		DQ496517.1	Lantang	Guangdong	Southeast China
		DQ496518.1	Lantang	Guangdong	Southeast China
		DQ496519.1	Lantang	Guangdong	Southeast China
		DQ496520.1	Lantang	Guangdong	Southeast China
		DQ496523.1	Lantang	Guangdong	Southeast China
		DQ496524.1	Lantang	Guangdong	Southeast China
		DQ496525.1	Lantang	Guangdong	Southeast China
		DQ496526.1	Lantang	Guangdong	Southeast China
		DQ496530.1	Lantang	Guangdong	Southeast China
		DQ496599.1	Putian	Fujian	Southeast China
		DQ496643.1	Xiang	Guizhou	Southwest China
		DQ496694.1	Meishan	Jiangxi	Southeast China
		DQ496697.1	Meishan	Jiangxi	Southeast China
		DQ496776.1	Wild boar	Fujian	Southeast China
		DQ496990.1	Yushan Black	Jiangxi	Southeast China
		EF545570.1	Wild boar	Yunnan	Southwest China
		EF590146.1	Wuzhishan	Hainan	Southeast China
		FJ601433.1	Wenchang	Hainan	Southeast China
		FJ601458.1	Qiandong Spotted	Guizhou	Southwest China
		FJ601459.1	Qiandong Spotted	Guizhou	Southwest China
		FJ601514.1	Ningxiang	Hunan	Southeast China
		HQ148323.1	Zang	Tibet	Plateau
		HQ148326.1	Zang	Tibet	Plateau
		JX068228.1	Zang	Tibet	Plateau
		KF472178.1	Ningxiang	Hunan	Southeast China
		KF660222.1	Qianshao Spotted	Hunan	Southeast China
		dWD2	ancient pig	Henan	North China
		dXLW2	ancient pig	Inner mongolia (East)	Northeast China
19	41	DQ152878.2	Yushan Black	Jiangxi	Southeast China
		DQ379153.2	Yimeng Black	Shandong	North China
		DQ379154.2	Yimeng Black	Shandong	North China
		DQ379155.2	Jinhua	Zhejiang	Southeast China
		DQ379156.2	Jiangquhai	Jiangsu	Southeast China
		DQ379157.2	Meishan	Zhejiang	Southeast China
		DQ379159.2	Meishan	Zhejiang	Southeast China
		DQ496415.1	Erhualian	Jiangsu	Southeast China
		DQ496416.1	Erhualian	Jiangsu	Southeast China
		DQ496417.1	Erhualian	Jiangsu	Southeast China
		DQ496418.1	Erhualian	Jiangsu	Southeast China
		DQ496419.1	Erhualian	Jiangsu	Southeast China
		DQ496420.1	Erhualian	Jiangsu	Southeast China
		DQ496498.1	Jiaozhou Black	Shandong	North China
		DQ496529.1	Lantang	Guangdong	Southeast China
		DQ496916.1	Wei	Anhui	Southeast China
		DQ496920.1	Wei	Anhui	Southeast China
		DQ496926.1	Wei	Anhui	Southeast China
		DQ496979.1	Yimeng Black	Shandong	North China
		DQ496980.1	Yimeng Black	Shandong	North China

		DQ496981.1	Yimeng Black	Shandong	North China
		DQ496983.1	Yimeng Black	Shandong	North China
		DQ496989.1	Yushan Black	Jiangxi	Southeast China
		DQ496994.1	Yushan Black	Jiangxi	Southeast China
		EF590198.1	Pudong White	Shanghai	Southeast China
		EU660191.1	Yimeng Black	Shandong	North China
		EU660195.1	Yimeng Black	Shandong	North China
		EU979153.1	Yimeng Black	Shandong	North China
		FJ601396.1	Jiaxing Black	Zhejiang	Southeast China
		JX068020.1	Jinhua	Zhejiang	Southeast China
		JX068021.1	Jinhua	Zhejiang	Southeast China
		JX068022.1	Jinhua	Zhejiang	Southeast China
		JX068037.1	Jinhua	Zhejiang	Southeast China
		JX068038.1	Jinhua	Zhejiang	Southeast China
		JX068044.1	Jinhua	Zhejiang	Southeast China
		JX068045.1	Jinhua	Zhejiang	Southeast China
		JX068049.1	Jinhua	Zhejiang	Southeast China
		JX068058.1	Jinhua	Zhejiang	Southeast China
		JX068061.1	Jinhua	Zhejiang	Southeast China
		dCS1	ancient pig	Hebei	Southeast China
		dWD3	ancient pig	Henan	North China
20	2	DQ152886.2	Huzhu	Qinghai	Plateau
		EU532157.1	Hezuo	Gansu	Plateau
21	7	DQ152887.2	Huzhu	Qinghai	Plateau
		DQ379179.2	Huzhu	Qinghai	Plateau
		DQ379180.2	Huzhu	Qinghai	Plateau
		DQ496452.1	Huzhu	Qinghai	Plateau
		DQ496457.1	Huzhu	Qinghai	Plateau
		DQ496458.1	Huzhu	Qinghai	Plateau
		JX068405.1	Pengzhou Mountain	Sichuan	Southwest China
22	3	DQ152888.2	Huzhu	Qinghai	Plateau
		DQ496451.1	Huzhu	Qinghai	Plateau
		JX068331.1	Zang	Gansu	Plateau
23	4	DQ496362.1	Dahe	Yunnan	Southwest China
		FJ601414.1	Dahe	Yunnan	Southwest China
		HQ148521.1	Wujin	Yunnan	Southwest China
		HQ148527.1	Gaoligongshan	Yunnan	Southwest China
24	10	DQ496392.1	Zang	Yunnan	Plateau
		EF590141.1	Leping Spotted	Jiangxi	Southeast China
		FJ601416.1	Leping Spotted	Jiangxi	Southeast China
		FJ601417.1	Leping Spotted	Jiangxi	Southeast China
		HQ148340.1	Zang	Tibet	Plateau
		HQ148341.1	Zang	Tibet	Plateau
		JX068251.1	Zang	Yunnan	Plateau
		JX068261.1	Zang	Yunnan	Plateau
		JX068272.1	Zang	Yunnan	Plateau
		JX068434.1	Pengzhou Mountain	Sichuan	Southwest China
25	6	DQ496428.1	Zang	Sichuan	Plateau
		DQ496433.1	Zang	Sichuan	Plateau
		DQ496454.1	Huzhu	Qinghai	Plateau
		JX068343.1	Zang	Gansu	Plateau

		JX068344.1	Zang	Gansu	Plateau
		JX068349.1	Zang	Gansu	Plateau
26	7	DQ496485.1	Jiaxing Black	Zhejiang	Southeast China
		DQ496964.1	Xiang	Guizhou	Southwest China
		DQ496966.1	Xiang	Guizhou	Southwest China
		DQ496967.1	Xiang	Guizhou	Southwest China
		DQ496968.1	Xiang	Guizhou	Southwest China
		DQ496969.1	Xiang	Guizhou	Southwest China
		EF545593.1	Xiang	Guizhou	Southwest China
27	3	DQ496598.1	Putian	Fujian	Southeast China
		EF590169.1	Guanling	Guizhou	Southwest China
		JX068446.1	Wujin	Sichuan	Southwest China
28	2	DQ496743.1	Wild boar	Yunnan	Southwest China
		DQ496875.1	Wild boar	Yunnan	Southwest China
29	3	DQ496764.1	Wild boar	Northeast China	Northeast China
		DQ496768.1	Wild boar	Northeast China	Northeast China
		DQ496770.1	Wild boar	Northeast China	Northeast China
30	19	DQ496773.1	Wild boar	Fujian	Southeast China
		DQ496774.1	Wild boar	Fujian	Southeast China
		DQ496775.1	Wild boar	Fujian	Southeast China
		DQ496779.1	Wild boar	Fujian	Southeast China
		DQ496780.1	Wild boar	Fujian	Southeast China
		DQ496781.1	Wild boar	Fujian	Southeast China
		DQ496782.1	Wild boar	Fujian	Southeast China
		DQ496784.1	Wild boar	Fujian	Southeast China
		DQ496785.1	Wild boar	Fujian	Southeast China
		DQ496786.1	Wild boar	Fujian	Southeast China
		DQ496815.1	Wild boar	Jiangxi	Southeast China
		DQ496819.1	Wild boar	Jiangxi	Southeast China
		DQ496828.1	Wild boar	Guizhou	Southwest China
		DQ496858.1	Wild boar	Yunnan	Southwest China
		DQ496894.1	Wild boar	Zhejiang	Southeast China
		DQ496906.1	Wild boar	Zhejiang	Southeast China
		DQ496915.1	Wei	Anhui	Southeast China
		DQ779418.1	Wild boar	Taiwan	Southeast China
		EF545571.1	Wild boar	Yunnan	Southwest China
31	3	DQ496791.1	Wild boar	Gansu	Northwest China
		DQ496836.1	Wild boar	Shaanxi	North China
		dWBP1	ancient pig	Jilin	Northeast China
32	1	DQ496792.1	Wild boar	Yunnan	Southwest China
33	15	DQ496793.1	Wild boar	Hainan	Southeast China
		DQ496795.1	Wild boar	Hainan	Southeast China
		DQ496796.1	Wild boar	Hainan	Southeast China
		DQ496797.1	Wild boar	Hainan	Southeast China
		DQ496800.1	Wild boar	Hainan	Southeast China
		DQ496804.1	Wild boar	Hainan	Southeast China
		DQ496805.1	Wild boar	Hainan	Southeast China
		DQ496807.1	Wild boar	Hainan	Southeast China
		DQ496809.1	Wild boar	Hainan	Southeast China
		DQ496811.1	Wild boar	Hainan	Southeast China
		DQ496812.1	Wild boar	Hainan	Southeast China

		DQ496813.1	Wild boar	Hainan	Southeast China
		DQ496889.1	Wild boar	Zhejiang	Southeast China
		FJ601523.1	Wild boar	Hainan	Southeast China
		FJ601524.1	Wild boar	Hainan	Southeast China
34	1	DQ496799.1	Wild boar	Hainan	Southeast China
35	5	DQ496802.1	Wild boar	Hainan	Southeast China
		DQ496806.1	Wild boar	Hainan	Southeast China
		DQ496808.1	Wild boar	Hainan	Southeast China
		DQ496890.1	Wild boar	Zhejiang	Southeast China
		DQ496891.1	Wild boar	Zhejiang	Southeast China
36	1	DQ496803.1	Wild boar	Hainan	Southeast China
37	1	DQ496835.1	Wild boar	Shaanxi	North China
38	3	DQ496843.1	Wild boar	Sichuan	Southwest China
		DQ496852.1	Wild boar	Yunnan	Southwest China
		EF545568.1	Wild boar	Yunnan	Southwest China
39	1	DQ496851.1	Wild boar	Yunnan	Southwest China
40	2	DQ496860.1	Wild boar	Yunnan	Southwest China
		EF545586.1	Wild boar	Jiangxi	Southeast China
41	1	DQ496876.1	Wild boar	Yunnan	Southwest China
42	3	DQ496877.1	Wild boar	Yunnan	Southwest China
		DQ496885.1	Wild boar	Yunnan	Southwest China
		EF545585.1	Wild boar	Jiangxi	Southeast China
43	2	DQ496892.1	Wild boar	Zhejiang	Southeast China
		DQ496893.1	Wild boar	Zhejiang	Southeast China
44	2	DQ518915.2	Lanyu	Taiwan	Southeast China
		EF375877.3	Lanyu	Taiwan	Southeast China
45	1	DQ779427.1	Wild boar	Taiwan	Southeast China
46	2	DQ779429.1	Wild boar	Taiwan	Southeast China
		DQ779430.1	Wild boar	Taiwan	Southeast China
47	1	EF536856.1	Kele	Guizhou	Southwest China
48	2	EF590155.1	Putian	Fujian	Southeast China
		FJ601480.1	Putian	Fujian	Southeast China
49	1	EF606873.1	Wild boar	Taiwan	Southeast China
50	3	EU008084.1	Wild boar	Taiwan	Southeast China
		GU147934.1	Wild boar	Taiwan	Southeast China
		NC_014692.1	Wild boar	Taiwan	Southeast China
51	1	EU333163.1	Wild boar	Northeast China	Northeast China
52	4	EU532150.1	Hezuo	Gansu	Plateau
		EU532152.1	Hezuo	Gansu	Plateau
		EU532159.1	Hezuo	Gansu	Plateau
		EU532163.1	Hezuo	Gansu	Plateau
53	1	EU532151.1	Hezuo	Gansu	Plateau
54	1	EU532153.1	Hezuo	Gansu	Plateau
55	1	EU532154.1	Hezuo	Gansu	Plateau
56	1	EU532155.1	Hezuo	Gansu	Plateau
57	2	EU532156.1	Hezuo	Gansu	Plateau
		EU532162.1	Hezuo	Gansu	Plateau
58	1	EU532158.1	Hezuo	Gansu	Plateau
59	1	EU532160.1	Hezuo	Gansu	Plateau
60	1	EU532161.1	Hezuo	Gansu	Plateau
61	1	EU660203.1	Yantai Black	Shandong	North China

62	1	FJ601506.1	Luchuan	Guangxi	Southwest China
63	1	GQ141892.1	Mashen	Shanxi	North China
64	1	GQ141900.1	Wild boar	Liaoning	Northeast China
65	4	HQ148316.1	Zang	Tibet	Plateau
		HQ148317.1	Zang	Tibet	Plateau
		JX068193.1	Zang	Tibet	Plateau
		JX068196.1	Zang	Tibet	Plateau
66	5	JX068314.1	Zang	Gansu	Plateau
		JX068319.1	Zang	Gansu	Plateau
		JX068342.1	Zang	Gansu	Plateau
		JX068345.1	Zang	Gansu	Plateau
		JX068348.1	Zang	Gansu	Plateau
67	3	JX068395.1	Pengzhou Mountain	Sichuan	Southwest China
		JX068396.1	Pengzhou Mountain	Sichuan	Southwest China
		JX068439.1	Pengzhou Mountain	Sichuan	Southwest China
68	2	JX068483.1	Wujin	Sichuan	Southwest China
		JX068484.1	Wujin	Sichuan	Southwest China
69	1	JX068485.1	Wujin	Sichuan	Southwest China
70	2	JX068515.1	Yanan	Sichuan	Southwest China
		dWD1	ancient pig	Henan	North China
71	1	dDSQ2	ancient pig	Inner mongolia (East)	Northeast China
72	1	dGCJ1	ancient pig	Henan	North China
73	1	dGCJ2	ancient pig	Henan	North China
